# Spiro[pyrrolidine-3, 3´-oxindole] as potent anti-breast cancer compounds: Their design, synthesis, biological evaluation and cellular target identification

**DOI:** 10.1038/srep32213

**Published:** 2016-08-30

**Authors:** Santanu Hati, Sayantan Tripathy, Pratip Kumar Dutta, Rahul Agarwal, Ramprasad Srinivasan, Ashutosh Singh, Shailja Singh, Subhabrata Sen

**Affiliations:** 1 Department of Chemistry, School of Natural Sciences, Shiv Nadar University, Dadri, Chithera, Gautam Buddha Nagar, 201314, Uttar Pradesh, India; 2 Department of Life Sciences, School of Natural Sciences, Shiv Nadar University, Dadri, Chithera, Gautam Buddha Nagar, 201314, Uttar Pradesh, India; 3 Shantani Proteome Analytics Pvt. Ltd. 100 NCL Innovation Park, Dr. HomiBhabha Road, Pune - 411 008, India

## Abstract

The spiro[pyrrolidine-3, 3´-oxindole] moiety is present as a core in number of alkaloids with substantial biological activities. Here in we report design and synthesis of a library of compounds bearing spiro[pyrrolidine-3, 3´-oxindole] motifs that demonstrated exceptional inhibitory activity against the proliferation of MCF-7 breast cancer cells. The synthesis involved a one pot Pictet Spengler-Oxidative ring contraction of tryptamine to the desired scaffolds and occurred in 1:1 THF and water with catalytic trifluoroacetic acid and stoichiometric N-bromosuccinimide as an oxidant. Phenotypic profiling indicated that these molecules induce apoptotic cell death in MCF-7 cells. Target deconvolution with most potent compound **5l** from the library, using chemical proteomics indicated histone deacetylase 2 (HDAC2) and prohibitin 2 as the potential cellular binding partners. Molecular docking of **5l** with HDAC2 provided insights pertinent to putative binding interactions.

Globally, breast cancer is one of the most common cause of fatalities in women. Nearly 12% of the world wide women population is affected by this debilitating disease. In 2012, 25% of cancer diagnosed women suffered from breast cancer. In 2008, it inflicted fatalities to nearly 0.5 million people all over the world^1^. Treatments for breast cancer involved mono and combination drug therapies, surgical and radiation techniques, novel targeted therapies and cancer vaccines^2^,^3^,^4^. Variety of breast cancer drugs such as Tamoxifen™, Letrozole™, Docetaxyl™ and etc. are being prescribed to the patients as preventive and curative treatments^5^,^6^,^7^. However undue toxicity and side effects in these medicines spoil their efficacy^8^,^9^,^10^. Consequently discovering novel,puissant small molecules as potential anti breast cancer drugs and with better safety profile is the need of the hour. Interestingly natural products provide a healthy source for such compounds. Among variety of natural product scaffolds the spiro[pyrrolidine-3, 3´-oxindole] scaffold forms the basic architectural motif in a plethora of natural as well as non-natural compounds that demonstrates pronounced anticancer activities. It belonged to a family of natural products that were first isolated from plants *Apocynaceae* and *Rubiacae* family^11^. The essential elements of these class of compounds is a pyrrolidine ring spiro fused at the 3^rd^ position of the oxindole moiety with diverse substitution on both the pyrrolidine and oxindole rings. For example Spirotryptostatin A and B,**1** and **2** inhibits tubulin polymerization and induces cell cycle inhibition of cancer cells at G2/M phase and spirooxindole MI-5, **3**, demonstrated novel type of inhibition of p53-MDM2 protein-protein interaction that is critical for modulating tumor suppressing ability of the p53-proteins (Fig. 1)^12^,^13^,^14^. These interesting therapeutic attributes of spiro[pyrrolidine-3, 3´-oxindole] scaffolds, make them attractive synthetic targets. There has been quite a few elegant chiral and achiral synthesis for this class of compound^15^. For example intramolecular Mannich reaction of tryptamine or a tryptophan-derived oxindole and an aldehyde, oxidative ring contraction of tetrahydro-β-carbolines, dipolar cycloaddition reactions of azomethineylides with oxindolylidene 3-ylidene acetate and intramolecular Heck reaction followed by trapping of an η^3^-allylpalladium species by a tethered nitrogen nucleophile^16^,^17^,^18^,^19^. There are also few one-pot synthesis to access this class of compounds, involving cycloaddition strategies^20^,^21^.

Here we report design of a library of spiro[pyrrolidine-3, 3´-oxindole] as potential anti breast cancer molecule that are synthesized *via* one pot Pictet Spengler-Oxidative ring contraction strategy of tryptamines mediated by N-bromosuccinimide (NBS) (Fig. 2). The compounds exhibited substantial inhibition in MCF-7 breast cancer cells where we could identify quite a few analogs with low micromolar EC_50_ values. Phenotypic profiling indicated that these compounds induce cell death through apoptosis (Fig. 2). A unique polymer technology based proteomics strategy divulged histone deacetylase 2 (HDAC2) and prohibitin 2 as the potential cellular binding partners (Fig. 2). Molecular docking of the most active compound in the library with HDAC2 revealed putative binding interaction that could be harnessed to achieve more potent molecules through target based approach.

## Results and Discussion

### Design

In the recent past, we had reported diversity oriented synthesis and phenotypic screening of spiropyrrolindole based potent antibreast cancer compounds **4**, which exhibited inhibition of proliferation of MCF-7 cells with an IC_50_ of ~24–500 nM (Fig. 3)^22^. Preliminary structure activity relationship (SAR) studies indicated that the aromatic or heteroaromatic functionality on the pyrrole moiety played a crucial role in imparting the inhibitory activity. Next we scanned the literature and could identify several indole derivatives such as Chaetochosins G, Vinorelbine (NVB), Vincristine and Vinblastine to name a few that have exhibited potential activity against breast cancer (Fig. 3)^23^,^24^,^25^,^26^. These findings highlighted the importance of spiropyrroloindole framework in breast cancer. Subsequently we conceived a library of spiro[pyrrolidine-3, 3´-oxindole] **5**. Taking a cue from the preliminary SAR of **4**, the design focused on diversifying the aromatic functionality of the pyrrole domain of **5** (Fig. 3).

### Chemistry

After close scrutiny of the existing strategies we realized that the oxidative ring contraction would be most amenable for our purpose as with modification it can provide the compounds in a combinatorial fashion. The original sequence involved Pictet Spengler reaction of tryptamine with appropriate aldehydes to afford tetrahydro-β-carboline, which subsequently undergo oxidative ring contraction in presence of water, appropriate acid and an oxidant to generate the spiro[pyrrolidine-3, 3´-oxindole]^27^,^28^,^29^,^30^. We envisioned a telescoping synthesis and subsequently remolded the sequence to make it a one pot procedure, where the intermediate tetrahydro-β-carboline need not be isolated. Tryptamine and p-tolualdehyde were used as model substrates. Variety of solvents such as tetrahydrofuran (THF), acetonitrile (ACN), 1, 4-dioxane, toluene, dichloromethane (DCM), ethylene glycol were used (Table 1, entry 1–7). All the reactions were started at 0 °C and were warmed to various temperatures ranging from rt → 100 °C. In most of the reactions the tetrahydro-β-carboline intermediate **4a** was isolated along with the desired compound **5a**. Finally the optimized protocolthat included one equivalent of tryptamine, one equivalent of p-tolualdehyde and 1.1 equivalent of N-bromosuccinimide in 1:1 THF and water with catalytic trifluoroacetic acid at 0 °C → rt afforded the desired compound **5a** exclusively in 81% yield (Table 1).

With the optimized procedure in hand, next we explored the scope of the reaction with variety of aromatic aldehydes and tryptamine. The reactions were conducted in parallel in a custom made 20 mL, glass carousal and the compounds were purified using Flash Master Parallel (10 channel) Automated Flash Chromatography. The reaction condition was amenable to both electron rich and electron poor aromatic aldehydes, affording the desired compounds **5b**-**5m** in 45–94% yield (Fig. 4). The electron poor aldehydes afforded the desired compounds **5b, d**-**e** and **5m** in relatively lower yields (45–60%) compared to the electron rich variant **5a**/**c** and **5f**-**l** (73–94%). Interestingly, during the reaction of 2, 5-trifluoromethyl benzaldehyde, the final product **5m** was obtained along with the corresponding tetrahydro-β-carboline intermediate in ~1:1 ratio. This, indicated that during oxidative ring contraction of the corresponding tetrahydro-β-carboline intermediate, the C_2_-C_7_ bond migration depended on the nature of the substituent on the aromatic ring adjacent to the migrated bond and that electron withdrawing functionalities deter the migration (Fig. 4). Finally the versatility of the protocol was further emphasized by the synthesis of the heteroaromatic analogs **5n** and **o** in 59 and 64% yield respectively.

### *In vitro* screening and phenotypic profiling

To assess anti breast cancer potential of our library of compounds we screened them at 50 μM concentration against MCF-7 breast cancer cells using MTT colorimetric cell viability assay (Table 2). Etoposide, the standard breast cancer drug was used as a positive control^31^. From the compounds screened several displayed significant inhibition of the proliferation of MCF-7 cells, with EC_50_ values in low micromolar range (Table 2). Preliminary structure-activity relationships demonstrate that electron withdrawing groups are tolerated at *meta* and electron donating groups at *ortho* and *para* positions. The monosubstituted analogs are more potent than multiple substituted aromatic ring. The 2, 5-bistrifluoromethyl- and 3, 4-bismethoxy-aryl analogs retained very weak activity (25–32% inhibition) whilst the unsubstituted analog **5l** exhibited best potency with an EC_50_ of 3.5 μM. It was interesting to observe that three of the most potent compounds **5e**, **i** and **l** from our library did not inhibit the proliferation of healthy mammalian COS-7 and MCF10A cell lines.

**Figure 1:**
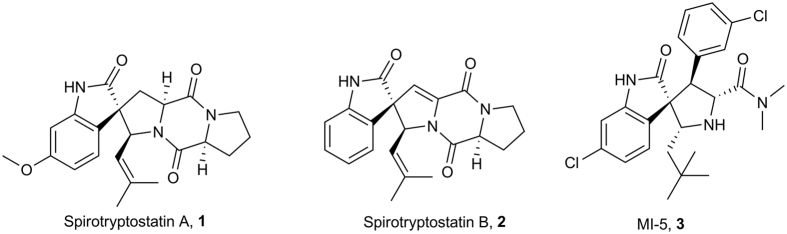
Representative spiro[pyrrolidine-3, 3´-oxindole] natural and non-natural bioactive compounds, Spirotryptostatin A (**1**) (inhibits tubulin polymerization), Spirotryptostatin B (**2**) (inhibits cancer cells at G2/M phase of the cell cycle) and MI-5 (**3**) (inhibits p53-MDM2 protein-protein interaction).

**Figure 2:**
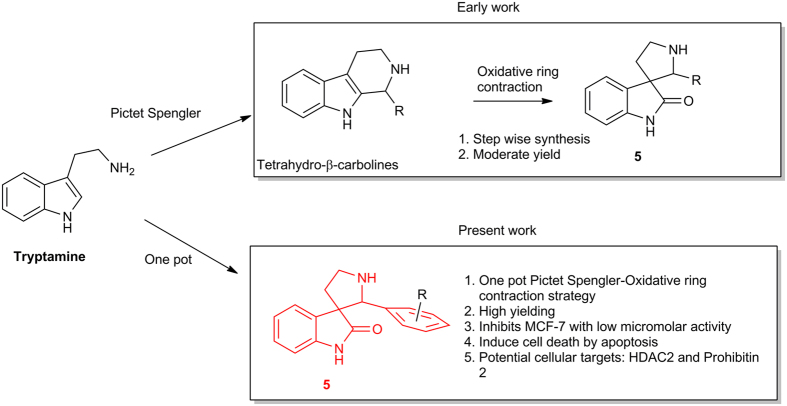
It depicts a comparison of our one pot strategy against step wise linear sequence of Pictet-Spengler and oxidative ring contraction reaction of tryptamine with appropriate aldehydes. One of the resulting compounds **5**, inhibits proliferation of MCF-7 cells and the putative binding targets are HDAC2 and prohibitin 2.

**Figure 3:**
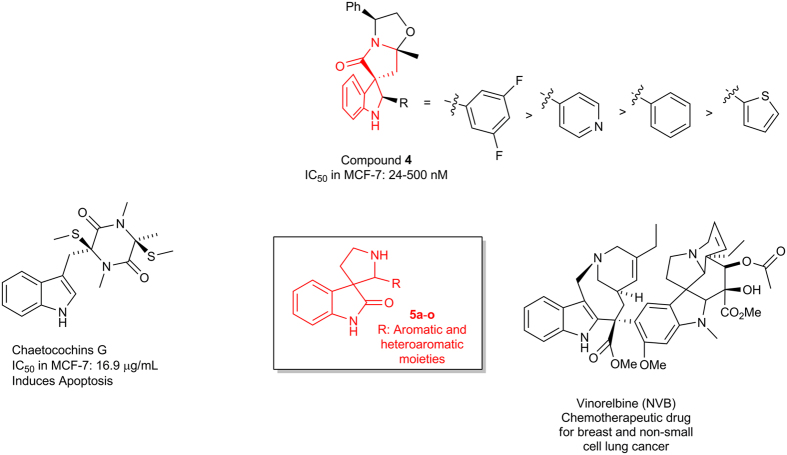
The new spiro[pyrrolidine-3, 3´-oxindole] scaffold based compounds, **5a**–**o** and other analogs were designed by drawing inspiration from natural products such as Chaetocochins G, compound **4** and Vinorelbine.

**Figure 4:**
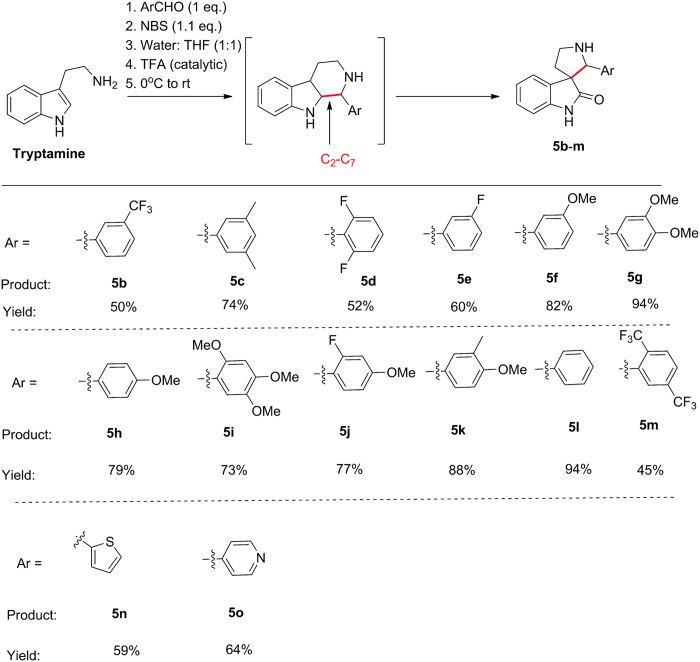
Synthesis of a library of spiro[pyrrolidine-3, 3´-oxindole] compounds 5**b**–**o** *via* one pot Pictet Spengler-oxidative ring contraction of tryptamine at room temperature with variety of aromatic aldehydes were executed.

Epithelial cell migration is an important characteristic of cancer cells. In a bid to evaluate if compound **5i** and **l**, inhibit cells migration, MCF-7 cells were treated with it. The cells were photographed after 24 hours and the migration distance was measured. The data indicated that both the compounds, indeed inhibit cell migration of MCF-7 cells compared to the untreated cells. Again etoposide was used as positive control (refer supplementary information-page 75). Further, to determine whether apoptosis (or programmed cell death) was the mechanism of cell death, DNA-ladder assay was used. During apoptosis, fragmentation of nuclear proteins and DNA molecule result in chromatin condensation and formation of apoptotic bodies which is manifested as typical ladder fragmentation pattern when analyzed by gel electrophoresis^32^. However, at times during this experiment instead of ladder fragmentation pattern, smearing is also observed, which indicates necrosis or killing of cells by autolysis^33^. Experiments with **5i** and **5l** resulted in smearing of DNA fragments thereby indicating that both the compounds induce necrosis to MCF-7 cells. No DNA internucleosomal fragmentation was observed in cells treated with 0.2% DMSO (vehicle control) (Figure 1, supplementary information). Additionally necrosis was also confirmed by Annexin-PI FACS based assay (Figure 2–11, supplementary information).

### Target deconvolution

**Figure 5:**
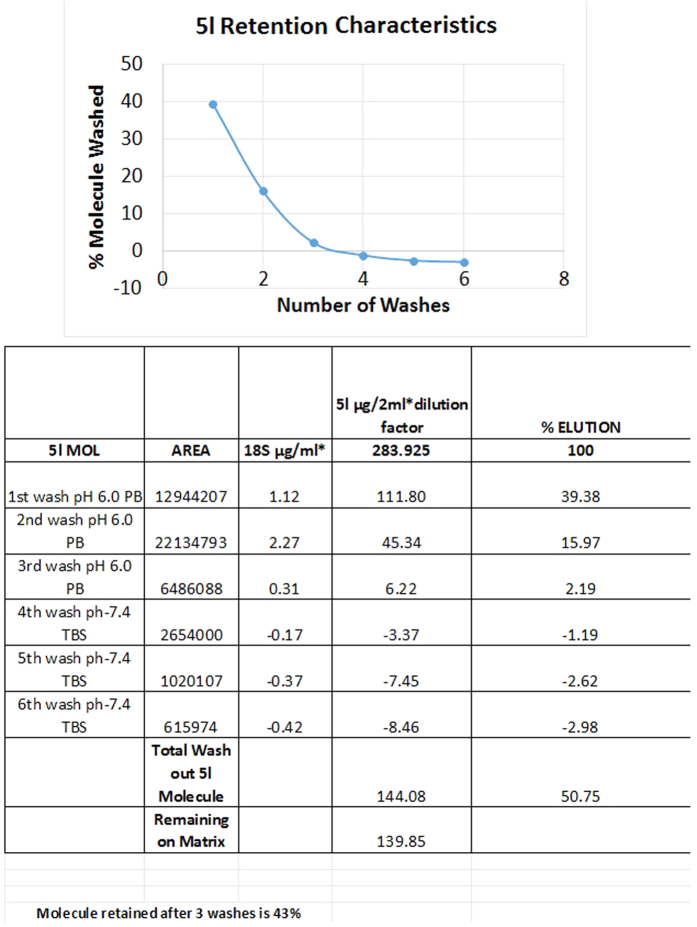
The retention characteristics of **5l** on the nitrocellulose matrix is shown above. HPLC-UV based method indicated 43% of **5l** got immobilized on the matrix.

Identifying cellular binding partners from a phenotypic screening is an onerous yet essential task. Recently proteomic tools are harnessed to achieve this. Herein we have identified cellular binding partners of **5l** (the most active compound in our library against MCF-7) by employing a chemical proteomics method that involved our unique polymer technology^34^. During the process **5l** was immobilized on a polymer (based on nitrocellulose) in aqueous buffer condition and was washed several times with 1X TBS, phosphate buffer. HPLC-UV based quantification of each buffer wash(refer supplementary information) indicated that 43% of **5l** got retained in the matrix (Fig. 5).

Next, polymer immobilized **5l** was incubated with MCF-7 cell lysate to facilitate drug-target interaction. After washing with 1X TBS, the **5l**-polymer complex bound proteins were recovered by acetone precipitation, dissolved in 1X sample buffer, separated on a 4–12% gradient gel and the bands were digested by standard “*in-gel*” trypsin digestion method^35^. The target capture experiments involved mass spectrometry analysis of the gel slices in an Agilent 1260 infinity HPLC-Chip/MS system and ran in triplicates where a total of 1838 protein in control and 2184 proteins in **5l** group were identified. The differential proteins (that are present in **5l** but not in control group) which were common in all the three experiments were identified as the putative targets and are enlisted in the table below (Table 3).

Of these seven proteins, Histone deacetylase 2 (HDAC2) and prohibitin2 (Table 3, entry 1 and 2) have been well demonstrated in scientific literature on their role in cancer. HDAC2 is a histone deacetylase enzyme that is over-expressed in various types of cancer including breast cancer^36^. Vorinostat, an HDAC inhibitor is an US-FDA approved drug for the treatment of lymphomas^37^. It is possible that **5l** exerts its anti-cancer activity by inhibiting HDAC2. Recent studies highlight that prohibitin is involved in estrogen dependent breast cancer^38^. It is a mitochondrial protein and is considered essential for structural integrity of mitochondrial membrane and is also involved in pro-survival mechanisms. Compounds that bind to Prohibitin-2 has been shown to induce apoptosis^39^. Several other mitochondrial proteins such as NAD(P) transhydrogenase and Alanine-tRNA ligase (Table 3, entry 5 and 6) could have possibly co-eluted along with Prohibitin. However, this needs careful interpretation of not losing sight that these proteins have been observed in every experiment as a differential protein and thus could be **5l** target on its own. Targets such as reticulon-4, Ribosome-binding protein 1, *t-*RNA (cytosine(34)-C(5))-methyltransferase (Table 3, entry 3, 4 and 7) needs further analysis as there is only a limited literature available on their role in any form of cancer or cellular toxicity. Hence HDAC2 and Prohibitin 2 are considered top priority targets for **5l** (Table 3).

### Molecular docking

**Figure 6:**
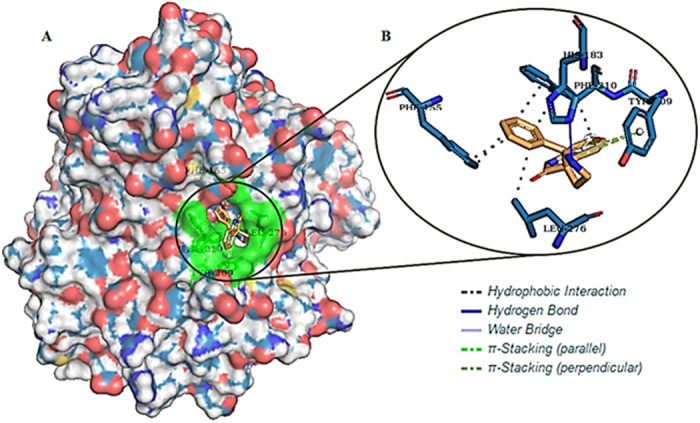
(**A**) 3D surface representation of HDAC2 is depicted, green color represents the active site residues with which our compound is binding. (**B**) Stick model represents the binding of **5l** with active site residues.

Next, we conducted molecular docking of **5l** with HDAC2 (Protein Data Bank [PDB] ID of 4LY1, this crystal structure is chosen as it has the best resolution (1.57 Å) among all HDAC2 crystal structures in PDB) to understand their putative binding interaction. Interestingly, it revealed several bonding and non-bonding interactions that could rationalize HDAC2 as the deconvoluted target for **5l** in MCF-7. There is a hydrogen bond between HIS183 residue and **5l** at a distance of 2.23Å. Several hydrophobic interactions were observed between **5l** and amino acids PHE155, PHE210 and LEU276. Finally two pi-stacking between **5l** and HDAC2 further strengthens their binding. The overall binding energy of **5l**-HDAC2 complex is -6.35 kcal/mol (Fig. 6).

## Discussion

Herein we discussed design of a library of spiro[pyrrolidine-3, 3´-oxindole] inspired from natural and non-natural products which are efficacious against breast cancer. The library synthesis involved a one pot Pictet Spengler-oxidative ring contraction strategy. In general it is a two-step procedure involving Pictet Spengler reaction between tryptamine and appropriate aldehydes to afford β-carbolines followed by NBS-mediated oxidative ring contraction. After a thorough exploration of reaction conditions we were enabled to telescope these two steps into one, which provided access to these compounds in moderate to excellent yields. Interestingly no aromatic ring brominated product was isolated as the byproduct during the reaction. This further highlights the efficiency of this process. Phenotypic screening against MCF-7 revealed several active compounds with EC_50_ in low μmolar range (**5e**, **i** and **l**). Etoposide was used as the +ve control and the activity of the most potent compounds, were comparable to it. Further phenotypic profiling indicated that these compounds induce necrotic cell death to MCF-7. Identifying cellular targets from phenotypes is an essential yet extremely tedious effort. Various strategies such as affinity chromatography, activity based protein profiling, label-free techniques, expression cloning techniques and *in silico* approaches have been harnessed to achieve this^40^,^41^,^42^,^43^,^44^. Our target deconvolution strategy with the most active compound **5l** included a unique polymer technology *via* nitrocellulose based polymer that deciphered HDAC2 and Prohibitin 2 as the potential cellular binding targets in MCF-7. Molecular modelling with HDAC2 and **5l** divulged putative binding interaction. This effort exhibited an efficient early drug discovery process involving phenotypic screening.

## Conclusion

In summary a novel compound **5l** has been discovered *via* a facile one pot Pictet Spengler-oxidative ring contraction strategy that exhibited substantial inhibition against MCF-7 cells with low μmolar EC_50_. It belonged to a library of compounds based on spiro[pyrrolidine-3, 3´-oxindole] motif. The library design was inspired from natural and synthetic products with similar architecture that displayed efficacy against breast cancer cells. Phenotypic profiling indicated that it induces cell death by necrosis pathway. A novel proteomics based target deconvolution strategy divulged HDAC2 and prohibit 2 as putative targets. Interestingly compounds **5e**, **i** and **l** selectively presented deleterious effects on MCF-7 and presented little or no effect on the corresponding primary cell line MCF10A. This is not surprising as these two cells are genetically different. Additionally it can also be hypothesized that HDAC2 which is the potential target for **5l**, is over expressed in MCF-7 and not in MCF10A. Presently SAR studies involving **5l** and related analogs, with HDAC2 as drug target (for breast cancer) is ongoing in our lab. The results will be reported in due course.

## Methods

### Chemical synthesis

All reactions were carried out in flame-dried tubes with magnetic stirring. Unless otherwise noted, all experiments were performed under argon atmosphere. All reagents were purchased from Sigma Aldrich, Acros or Alfa Aesar. Solvents were treated with 4 Å molecular sieves or sodium and distilled prior to use. ^1^H NMR and ^13^C NMR spectra were recorded with tetramethylsilane (TMS) as internal standard at ambient temperature unless otherwise indicated Bruker 500 MHz for 1 H NMR and 125 MHz for ^13^C NMR. Chemical shifts are reported in parts per million (ppm) and coupling constants are reported as Hertz (Hz). Splitting patterns are designated as singlet (s), broad singlet (bs), doublet (d), triplet (t). Splitting patterns that could not be interpreted or easily visualized are designated as multiple (m). The Mass Spectrometry analysis was done on the 6540 UHD Accurate-Mass Q-TOF LC/MS system (Agilent Technologies) equipped with Agilent 1290 LC system obtained by the Dept. of Chemistry, School of Natural Sciences, Shiv Nadar University, Uttar Pradesh 201314, India.

MCF10A was obtained from American Tissue Type Culture Collection (ATCC, Bethesda, MD). All cells were grown in a humidified atmosphere at 37 °C, with 5% CO_2_ in air.

### Cell culture conditions

MCF-7 cells were maintained as monolayer in 25 cm^2^ culture flasks (T-25) at 37 °C in DMEM medium (GIBCO, St. Louis, USA) supplemented with high glucose, L-glutamine, pyridoxine hydrochloride, 110 mg/L sodium pyruvate, 3.7 g/L NaHCO_3_ and antibiotics (Penicillin-10,000 units/mL, Streptomycin −10,000 μg/mL, GIBCO, USA). Growth medium (pH 7.4) was prepared by adding 5% heat inactivated FBS (Fetal Bovine Serum, GIBCO, USA) and stored at 4 °C. The medium was changed 10–18 hrs prior to experiment, and cells were confluent at the time of experimentation. Cells were dislodged from flasks by gentle trypsinization containing 0.25% Trypsin. MCF-7 cells were regularly sub-cultured thrice a week in a seeding density of 40,000–50,000 cells/cm^2^area. Cell confluency = 70%

### Cell Viability Assay (MTT Assay)

The MTT colorimetric viability assay was performed to evaluate the anticancer effect of compounds. 30,000 cells/100 μl of media were seeded per well and allowed to stretch and adhere overnight at 37 °C. Following day media was removed and 100 μl fresh media was added to the grown cells. Adhered cells were then treated with the compounds **5a**-**m** dissolved in DMSO in triplicates at a standard concentration of 50 μM for 48 hours. There was one positive and one negative control along with 13 compounds. The results were measured all in triplicate in 45 wells of 96 well plate at each time point. Cytotoxic effect was evaluated using ability of live cells to cleave MTT ((3-[4, 5- dimethylthiazol-2-yl]-2, 5-diphenyl tetrazolium bromide)) (Sigma-Aldrich, St Louis, MO, USA), into formazan crystals. Post 48 hours of treatment, 10 μl of MTT (5 mg/ml in PBS) was added to the cells and incubated for 4 hours in dark at 37 °C. The violet colored crystals formed were then dissolved in 100 μl DMSO solvent (Dimethyl Sulfoxide, Sigma-Aldrich, St. Louis, MO, USA). The colorimetric assay was read by a MultiMode Plate Reader (Bio-Rad) at 595 nm. Percentage of Inhibition of drug was calculated by following formula:


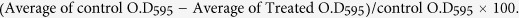


### Cell Migration assays

MCF-7 cells were grown to confluence in six-well tissue culture plastic dishes to a density of ∼0.2 million cells/well. After 24 hrs of seeding, cells were treated with chemical compounds. The medium was removed and the cells were scratched by 200 μL pipette micro tip through the center of the plate. The cultures were washed to remove debris and re-cultured with DMEM containing 10% FBS for cell migration. The cells were imaged at 24 hrs and the migrated distance was measured. Migration rate was calculated by counting five to seven fields per image and the results presented as the average of three independent experiments performed over multiple days.

### DNA fragmentation assay

The DMSO (dimethyl sulphoxide)–SDS (sodium dodecyl sulphate)–TE (Tris-EDTA) method: 2 million MCF-7cells were taken as starting material for DNA isolation. Cells were dislodged with Trypsin-EDTA and washed with 1 X PBS. DMSO was added directly to the cell pellet (100 μl) and mixed well by pipetting. TE buffer (pH 7.4) with 2% SDS was added into the mixture with same volume, followed by pipetting. The solution was centrifuged at 3000 rpm at 4 °C and 30 μL of the supernatant was loaded on agarose gel to check the effect of drugs on DNA molecule.

### Annexin V/propidium iodide staining

The assay is based on the ability of the protein annexin V to bind to phosphatidylserine (PS) exposed on the outer membrane leaflet in apoptotic cells (PS also appears on the necrotic cell surface). The annexin V assay was performed following the manufacturer’s instructions (Annexin V-FITC kit,Alexa Fluor). Briefly, the cells were washed with PBS and suspended in pre-diluted binding buffer. The cell density was adjusted to 2 million and 195 μl of cell suspension were mixed with 5 μl Annexin V-FITC and incubated for 10 min in the dark at room temperature. The cells were washed with binding buffer, re-suspended in 195 μl binding buffer, counterstained with 10 μl of the 20 μg/ml propidium iodide stock (final concentration 1 μg/ml) and the data were acquired by BD FACS diva 7.0 at 6 h, 12 h and 24 h.

### Protein Deconvolution

#### Immobilization of 5l

**5l** was provided as a lyophilized powder. A 100 mM stock of **5l** was made in DMSO. The stock solution was diluted to 215 μM in 1 mM of phosphate buffer in pH 6.0 and incubated with the matrix for 16 hrs at room temperature. After incubation, the matrix was washed five times with Phosphate buffer. The amount of molecule in the phosphate buffer wash was quantified by HPLC-UV based method and amount of molecule retained on the matrix was calculated.

#### Affinity enrichment of target proteins on 5l immobilized matrix

Cell lysates were prepared in RIPA buffer and protein concentration was determined by Bradford method. Cell lysate was diluted with 1X TBS to a concentration of 1 mg/ml. The **5l** immobilized matrix was incubated with 2 ml of cell lysate for 2 min with mild shaking. The matrix was washed with 1X TBS to remove unbound proteins and to reduce non-specific interactions. The molecule bound proteins were then recovered by acetone precipitation and was dissolved in 1X sample buffer. Proteins were separated on a 4–12% gradient gel and bands were digested by standard *“in-gel”* trypsin digestion methods.

### Mass spectrometry analysis of proteins

#### Protein Reduction, Alkylation and Digestion

Clearly identified gel-bands were cut in typical size of 1 mm band from the gel and placed in 1.5 ml tubes. Gel slices were washed once with distil water and then twice, with 100 μL of 100 mM ABC/50% acetonitrile (ACN) solution by placing the bands in each respective solution at room-temperature for 30 minutes. After discarding the last wash gel-slices were incubated with 50 μL of 10 mM DTT at 56 °C for 10 minutes in a dry bath. Tubes were then cooled and then gel-slices were incubated with 50 μL of 100 mM IAA at 37 °C for 30 minutes. IAA solution was removed and gel slices were rinsed by incubating it with 100 mM ABC/50% ACN at 37 °C for 15 minutes. Rinse solution was removed and gel-slices were dehydrated by incubating them with 100 μl of 100% ACN for 10 minutes. ACN was removed and gel-slices were dried using a Speed Vac for 45–60 minutes at room temperature. Gel-slices were then incubated with 20–30 μl of 20 μg/ml trypsin solution for an hour at room temperature. 50 μl of 50 mM ABC was added to the gel-slices and completely covered tubes were placed at 37 °C for 12 hours for maximum trypsin digestion of the protein. Gel-slices were then incubated with 150 μl of HPLC water for 10 minutes. Solution was frequently mixed using vortex mixer. Supernatant was removed and saved after appropriate labelling. Gel-slices were then incubated with 100 μl of 50%ACN/5% TFA at 37 °C for 60 minutes to extract the peptides from gel-slices. Supernatant was collected and pooled with earlier collected supernatant. Supernatant was then concentrated at room temperature until moderate dryness. Dried peptides were dissolved in 5 μl of 0.1%TFA/HPLC Water solution and set-aside for mass-spectrometry analysis.

#### HPLC-CHIP-MS

Refer supplementary information.

#### Spectra Analysis and Protein Database Searches

Agilent Mass Hunter software was used for data acquisition and analysis of Total ion chromatograms. Protein searches were carried out using Morpheus software tools using the Human proteome database. Auto-validation protocol having <1% False Detection Rate was used in confirming the identity of protein. The peptide sequence was also analysed for variable modifications such as methionine oxidation, acetylation, cysteine sulfoxide, deamidation and pyro glutamic acid modification.

#### Molecular docking

Molecular docking was performed using Molecular Operating Environment (MOE) version 2014.0901, Chemical Computing Group Inc., Montreal, Canada^45^. The X-ray crystal structure of the Histone deacetylase 2 (HDAC2) was obtained from Protein Data bank (PDB). The PDB ID of the protein is 4LY1, this crystal structure is chosen as this is having the best resolution (1.57 Å) among all HDAC2 crystal structures. The structure was prepared using LigX module of the MOE. This preparation involved addition of hydrogen atoms and addition of any missing residues or atoms. Energy minimization is done using AMBER 10: EHT force fields. The 3D structure of the compound was constructed using the builder interface of the MOE program. Docking of the compound was done using MOE-DOCK module in MOE. The active site of the target protein is defined by the inhibitor present in the crystal structure. Triangle matcher is used as the placement methodology and London dG is used as a scoring methodology. The best pose was selected on the basis of the best score.

## Additional Information

**How to cite this article**: Hati, S. *et al.* Spiro[pyrrolidine-3, 3´-oxindole] as potent anti-breast cancer compounds: Their design, synthesis, biological evaluation and cellular target identification. *Sci. Rep.* **6**, 32213; doi: 10.1038/srep32213 (2016).

## Supplementary Material

Supplementary Information

## Figures and Tables

**Table 1:**
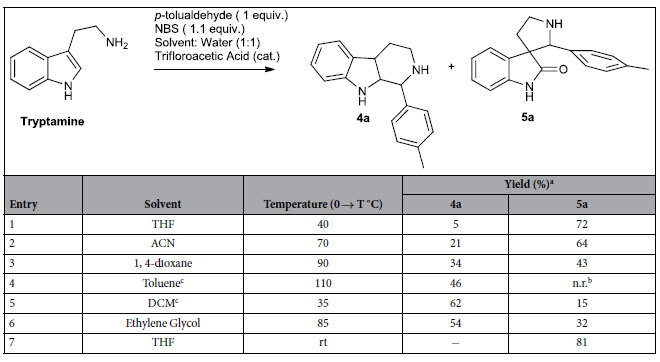
Reaction optimization of one pot Pictet Spengler-Oxidative ring contraction of tryptamine to furnish spiro[pyrrolidine-3, 3´-oxindole].

^a^Isolated yield.

^b^n. r.: no reaction.

^c^Tetrabutyl ammonium bromide (TBAB).

**Table 2:** *In vitro* phenotypic activity of the library of spiro[pyrrolidine-3, 3´-oxindole] against MCF-7 and COS-7 cells.

Entry	Compound	Percentage inhibition^a^			EC_50_(μM)^b^
		MCF-7	COS-7	MCF10A	MCF-7
1	**5a**	35.06 ± 0.09	11.73 ± 0.09	−	−
2	**5b**	33.10 ± 0.12	20.73 ± 0.11	−	−
3	**5c**	34.46 ± 0.11	28.80 ± 0.13	−	−
4	**5d**	44.25 ± 0.17	9.22 ± 0.12	−	−
5	**5e**	54.67 ± 0.08*	3.56 ± 0.19*	4.48	6.00*
6	**5f**	38.48 ± 0.18	34.87 ± 0.17	−	−
7	**5g**	25.29 ± 0.24	2.39 ± 0.23	−	−
8	**5h**	50.62 ± 0.24	25.20 ± 0.06	−	−
9	**5i**	53.66 ± 0.06*	15.60 ± 0.09*	0.8	4.01*
10	**5j**	38.72 ± 0.15	7.52 ± 0.15	−	−
11	**5k**	44.40 ± 0.09	7.14 ± 0.19	−	−
12	**5l**	60.96 ± 0.09*	15.42 ± 0.18*	0.08	3.53*
13	**5m**	31.81 ± 0.11	−0.51 ± 0.29	−	−
14	**Etoposide**	71.95 ± 0.11	77.65 ± 0.10	−	−

^a^All data is the mean of one experiment conducted in triplicates.

^b^EC_50_ of **5e**, **5i** and **5l** is the mean ± SD of three experiments.

^*^Unless otherwise indicated, the differences were considered to be statistically significant at P < 0.05. The analyses were performed using GraphPad Prism Software version 5.02 (GraphPad Inc., La Jolla, CA, USA).

**Table 3:** List of putative targets after target deconvolution.

Entry	UniProt ID	Protein Description	Protein Class
1	Q92769	Histone deacetylase 2 (HDAC2)	Nuclear Protein/Enzyme
2	Q99623	Prohibitin 2	Structural
3	Q9P2E9	Ribosome-binding protein 1	Receptor
4	Q08J23	tRNA (cytosine (34)-C(5))-methyltransferase	Enzyme
5	Q5JTZ9	Alanine—tRNA ligase, mitochondrial	Enzyme
6	Q13423	NAD(P) transhydrogenase, mitochondrial	Enzyme
7	Q9NQC3	Reticulon-4	Growth Factor
