# Supplementary material for: Spiro[pyrrolidine-3, 3´-oxindole] as potent anti-breast cancer compounds: Their design, synthesis, biological evaluation and cellular target identification
Source: Sci Rep. 2016 Aug 30;6:32213. doi: 10.1038/srep32213 (PMC5004205; doi:10.1038/srep32213)
Supplement: Supplementary Information [file srep32213-s1.pdf]

Spiro[pyrrolidine-3, 3´-oxindole] as potent anti-breast cancer compounds: Their design, synthesis, biological evaluation and cellular target identification

Santanu Hati,[1] Sayantan Tripathy,[2] Pratip Kumar Dutta,[1] Rahul Agarwal,[2] Ramprasad Srinivasan,[3] Ashutosh Singh,[2] Shailja Singh,[2] and Subhabrata Sen[1,] *

1. Department of Chemistry, School of Natural Sciences, Shiv Nadar University, Dadri, Chithera, Gautam Buddha Nagar, 201314, Uttar Pradesh, India. Email: Subhabrata.sen@snu.edu.in
2. Department of Life Sciences, School of Natural Sciences, Shiv Nadar University, Dadri, Chithera, Gautam Buddha Nagar, 201314, Uttar Pradesh, India.
3. Shantani Proteome Analytics Pvt. Ltd. 100 NCL Innovation Park, Dr. Homi Bhabha Road, Pune-411008, India

1. Chemistry
2. Biology
3. NMR spectra
4. HPLC spectra
5. HPLC-MS-Chip information for target deconvolution
6. Cell migration studies
7. Target validation for unique polymer technology (UPT) for target deconvolution

Chemistry

All reactions were carried out in flame-dried tubes with magnetic stirring. Unless otherwise noted, all experiments were performed under argon atmosphere. All reagents were purchased from Sigma Aldrich, Acros or Alfa Aesar. Solvents were treated with 4 Å molecular sieves or sodium and distilled prior to use. Purifications of reaction products were carried out by Flash chromatography using Biotage's 10 channel chromatographic system . ^{1}H NMR and ^{13}C NMR spectra were recorded with tetramethylsilane (TMS) as internal standard at ambient temperature unless otherwise indicated Bruker 500 MHz for ^{1}H NMR and 120 MHz for ^{13}C NMR. Chemical shifts are reported in parts per million (ppm) and coupling constants are reported as Hertz (Hz). Splitting patterns are designated as singlet (s), broad singlet (bs), doublet (d), triplet (t). Splitting patterns that could not be interpreted or easily visualized are designated as multiple (m). The Mass Spectrometry analysis was done on the 6540 UHD Accurate-Mass Q-TOF LC/MS system (Agilent Technologies) equipped with Agilent 1290 LC system obtained by the Dept. of Chemistry, School of Natural Sciences, Shiv Nadar University, Uttar Pradesh 203207, India.

General procedure for the synthesis of 3, 3´-spiropyrrolooxoindole

To a solution of the tryptamine (1.0 eq.) in 1:1 THF/water (20 mL), was added appropriate benzaldehydes (1 eq.), catalytic trifluoroacetic acid (TFA)and the reaction mixture was cooled to 0°C, followed by portion wise addition of N-bromosuccinimide (NBS) (1.1 eq.).The resulting solution was stirred at 0°C for 6h after which it was gradually warmed to room temperature (rt). Once TLC confirms the total consumption of the starting imine, the reaction was quenched with saturated sodium carbonate solution. The aqueous layer was extracted twice with dichloromethane. The organic extracts were combined and washed with brine, dried over anhydrous magnesium sulphate and was evaporated to provide the crude compound. It was purified by flash column chromatography using a mixture of ethyl acetate and hexane as eluent to afford the desired 3, 3´-spiropyrroloxoindole **5a-m**.

2'-(p-tolyl)spiro[indoline-3,3'-pyrrolidin]-2-one (**5a**)

Following the general procedure tryptamine (200 mg, 1.25 mmol), *p*-tolualdehyde (150 mg, 1.25 mmol) 1:1 THF/water (20 mL) with catalytic TFA and NBS (197.5 mg, 1.37 mmol) provided the desired compound **5a** in 260 mg (yield 75%) as viscous oil.^{1}H NMR (500 MHz; DMSO-d_6):

9.91 (s, 1H); 7.41 (d, *J* =10 Hz, 1H); 7.21 (d, *J* = 10 Hz, 1H); 7.18-7.13 (m, 4H); 7.02-6.99 (t, *J* = 10 Hz, 1H); 6.97-6.93 (t, *J* = 10 Hz, 1H); 4.37 (s, 1H); 3.09-3.06 (m, 1H); 2.95-2.92 (m, 1H); 2.76-2.72 (m, 1H); 2.69-2.65 (m, 1H); 2.30 (s, 3H). ^{13}C NMR (125 MHz; DMSO-d_6): 181.1, 140.0, 136.3, 135.9, 135.4, 128.7, 128.4, 126.8, 120.5, 118.2, 111.0, 71.3, 59.5, 45.1, 37.2, 20.7. HRMS (ESI-TOF) m/z: $[M + H]^+$Calcd for $C_{18}H_{19}N_2O$ - 279.1492, Found - 279.1468; IR 3216.67, 2907.60, 1698.53, 1469.84.

2'-(3-(trifluoromethyl)phenyl)spiro[indoline-3,3'-pyrrolidin]-2-one (**5b**)

Following the general procedure tryptamine (200 mg, 1.25 mmol),3-trifluoromethylbenzaldehyde (217.6 mg, 1.25 mmol), 1:1 THF/water (20 mL),catalytic TFA and NBS (197.5 mg, 1.37mmol) provided the desired compound **5b** in 289 mg (yield 70%) as gummy solid. ^{1}H NMR (500 MHz; DMSO-d_6): 10.00 (s, 1H); 7.53-7.49 (m, 2H); 7.37-7.34 (t,*J* = 10 Hz, 1H); 7.21 (s, 1H); 7.17-7.16 (t, *J* = 5 Hz, 1H); 7.11 (d, *J* = 10 Hz, 1H); 7.07-7.04 (t, *J* = 5 Hz, 1H); 6.67 (d, *J* = 5 Hz, 1H);4.46 (s, 1H); 3.53-3.49 (m, 1H); 3.47-3.43 (m, 1H); 3.17-3.11 (m, 1H); 3.06-3.01 (m, 1H) . ^{13}C NMR (125 MHz; DMSO-d_6): 179.5, 142.0, 139.2, 131.8, 130.1, 128.6, 127.9, 123.9, 123.9, 122.9, 122.8, 122.8, 121.8, 109.0, 72.2, 59.0, 45.9, 37.5.HRMS (ESI-TOF) m/z: $[M + H]^+$Calcd for $C_{18}H_{16}F_3N_2O$ - 333.1209, Found - 333.1180; IR 3252.51, 3006.19, 1674.57, 1440.16.

2'-(3,5-dimethylphenyl)spiro[indoline-3,3'-pyrrolidin]-2-one (**5c**)

Following the general procedure tryptamine (200 mg, 1.25 mmol), 3, 5-dimethylbenzaldehyde (167.7 mg, 1.25 mmol), 1:1 THF/water (20 mL)with catalytic TFA and NBS (197.5 mg, 1.37 mmol) provided the desired compound **5c** in 271 mg (yield 74%) as yellow liquid. ^{1}H NMR (500 MHz; DMSO-d_6): 9.98 (s, 1H); 7.54 (d, *J* =10 Hz, 1H); 7.32 (d, *J* = 5 Hz, 1H); 7.14-7.11 (m, 2H); 7.07-7.04 (t, *J* = 10 Hz, 1H); 7.02 (s, 2H); 4.24 (s, 1H); 3.49-3.41 (m, 2H); 3.15-3.11 (m, 1H); 3.04-3.00 (m, 1H); 2.29 (s, 6H). ^{13}C NMR (125 MHz; DMSO-d_6):179.9, 138.1, 136.6, 134.7, 131.2, 128.6, 127.5, 125.7, 121.9, 119.1, 111.7, 71.0, 54.9, 44.7, 37.7, 20.9. HRMS (ESI-TOF) m/z: $[M + H]^+$Calcd for $C_{19}H_{21}N_2O$ - 293.1648, Found - 293.1623; IR 3230.46, 2923.48, 1673.98, 1470.75.

2'-(2,6-difluorophenyl)spiro[indoline-3,3'-pyrrolidin]-2-one (**5d**)

Following the general procedure tryptamine (200 mg, 1.25 mmol), 2, 6-difluorobenzaldehyde (177.5 mg, 1.25 mmol), 1:1 THF/water (20 mL) with catalytic TFA and NBS (197.5 mg, 1.37 mmol) provided the desired compound **5d** in 291 mg (yield 78%) as colorless liquid. ^{1}H NMR (500 MHz; DMSO-d$_6$): 10.34 (s, 1H); 7.66-7.63 (m, 1H); 7.55-7.51 (m, 1H); 7.39-7.37 (m, 1H); 7.29-7.23 (m, 2H); 7.12-7.10 (m, 1H); 7.06-7.04 (m, 1H); 4.91 (s, 1H); 3.62-3.60 (m, 1H); 3.51-3.49 (m, 1H); 3.12-3.09 (m, 1H), 3.03-3.01 (m, 1H). ^{13}C NMR (125 MHz; DMSO-d$_6$):181.9, 162.9, 160.9, 138.7, 136.9, 128.5, 126.5, 122.4, 119.7, 118.8, 113.2, 113.1, 112.1, 66.3, 48.2, 29.9. HRMS (ESI-TOF) m/z: $[M + H]^+$Calcd for $C_{17}H_{15}F_2N_2O$ - 301.114, Found - 301.1116; IR 3253.06, 2962.24, 1667.18, 1473.61.

2'-(3-fluorophenyl)spiro[indoline-3,3'-pyrrolidin]-2-one (**5e**)

Following the general procedure tryptamine (200 mg, 1.25 mmol), 3-fluorobenzaldehyde (155 mg, 1.25 mmol) 1:1 THF/water (20 mL) with catalytic TFA and NBS (197.5 mg, 1.37 mmol) provided the desired compound **5e** in 310 mg (yield 88%) as light yellow liquid. ^{1}H NMR (500 MHz; DMSO-d$_6$): 10.25 (s, 1H); 7.63-7.60 (m, 2H); 7.58-7.55 (m, 1H); 7.52 (d, J = 10 Hz, 1H); 7.44 (d, J = 10 Hz, 1H); 7.40-7.36 (t, J = 10 Hz, 1H); 7.24-7.20 (t, J = 10 Hz, 1H); 7.10-7.07 (t, J = 10 Hz, 1H); 4.64 (s, 1H); 3.60-3.57 (m, 1H); 3.50-3.46 (m, 1H); 3.14-3.06 (m, 1H), 3.05-2.96 (m, 1H).^{13}C NMR (125 MHz; DMSO-d$_6$):181.3, 161.2, 139.8, 137.0, 130.6, 127.2, 124.8, 124.2, 123.9, 119.6, 116.7, 114.8, 112.8, 65.8, 47.9, 29.3. HRMS (ESI-TOF) m/z: $[M + H]^+$Calcd for $C_{17}H_{16}FN_2O$-283.1241,Found - 283.1210; IR 3392.30, 2918.94, 2848.13, 1485.07.

2'-(3-methoxyphenyl)spiro[indoline-3,3'-pyrrolidin]-2-one(**5f**)

Following the general procedure tryptamine (200 mg, 1.25 mmol), 3-methoxybenzaldehyde (170.2 mg, 1.25 mmol), 1:1 THF/water (20 mL) with catalytic TFA and NBS (197.5 mg, 1.37 mmol) provided the desired compound with catalytic TFA and NBS (158 mg, 1.1 mmol) in water (10 mL) provided the desired compound **5f** in 301 mg (yield 82%) as light yellow oil. ^{1}H NMR (500 MHz; DMSO-d$_6$): 9.96 (s, 1H); 7.41 (d,J = 10 Hz, 1H); 7.26-7.22 (m, 2H); 7.02-6.99 (t,J = 10 Hz, 1H); 6.96-6.93 (t, , J = 5 Hz, 1H); 6.88-6.85 (t, , J = 5 Hz ,3H); 4.37 (s, 1H); 3.73 (s, 3H), 3.10-3.07 (m, 1H); 2.95-2.92 (m, 1H); 2.75-2.72 (m, 1H), 2.68-2.63 (m, 1H). ^{13}C NMR (125 MHz; DMSO-d$_6$):180.9, 159.2, 144.6, 135.9, 135.2, 129.1, 126.8, 120.6, 120.5,

117.5, 114.2, 112.5, 111.1, 71.2, 59.8, 56.6, 45.1, 37.2. HRMS (ESI-TOF) m/z: $[M + H]^+$Calcd for $C_{18}H_{19}N_2O_2$ - 295.1441,Found -295.1420; IR 3299.7, 2895.16, 1698.53, 1596.14.

2'-(3,4-dimethoxyphenyl)spiro[indoline-3,3'-pyrrolidin]-2-one(**5g**)

Following the general procedure tryptamine (200 mg, 1.25 mmol), 3, 4-dimethoxybenzaldehyde (207.5 mg, 1.25 mmol), 1:1 THF/water (20 mL) with catalytic TFA and NBS (197.5 mg, 1.37 mmol) provided the desired compound **5g** in 381 mg (yield 94%) as colorless liquid.^{1}H NMR (500 MHz; DMSO-d_6): 9.83 (s, 1H); 7.40 (d, *J* = 10 Hz, 1H); 7.23 (d, *J*= 5 Hz ,1H); 7.01-6.98 (t, *J* = 10 Hz, 1H); 6.96-6.94. (m, 2H); 6.89 (d, *J* = 10 Hz, 1H); 6.76 (d, *J* = 5 Hz,1H); 4.32 (s, 1H); 3.73 (s, 3H), 3.71 (s, 3H); 3.13-3.11 (m, 1H); 2.94-2.92 (m, 1H); 2.76-2.73 (m, 1H), 2.67-2.63 (m, 1H). ^{13}C NMR (125 MHz; DMSO-d_6):181.2, 148.6, 148.1, 135.9, 135.8, 135.6, 126.9, 120.4, 118.1, 117.5, 112.2, 111.4, 111.0, 71.4, 59.9, 55.6, 55.4, 44.9, 37.3. HRMS (ESI-TOF) m/z: $[M + H]^+$Calcd for $C_{19}H_{21}N_2O_3$- 325.1547, Found 325.1550.

2'-(4-methoxyphenyl)spiro[indoline-3,3'-pyrrolidin]-2-one (**5h**)

Following the general procedure tryptamine (200 mg, 1.25 mmol), 3-methoxybenzaldehyde (170.2, 1.25 mmol), 1:1 THF/water (20 mL) with catalytic TFA and NBS (197.5 mg, 1.37 mmol) provided the desired compound**5h** in 291 mg (yield 79%) as colorless liquid. ^{1}H NMR (500 MHz; DMSO-d_6): 9.86 (s, 1H); 7.41 (d,*J* = 10 Hz, 1H); 7.23-7.19 (m, 3H); 7.02-6.99 (t, *J* = 10 Hz, 1H); 6.97-6.93 (t, , *J* = 10 Hz, 1H); 6.90 (d, , *J* = 10 Hz ,2H); 4.38 (s, 1H); 3.74 (s, 3H), 3.10-3.08 (m, 1H); 2.96-2.93 (m, 1H); 2.75-2.72 (m, 1H), 2.69-2.66 (m, 1H). ^{13}C NMR (125 MHz; DMSO-d_6):181.0, 158.6, 135.9, 135.4, 134.7, 129.6, 126.8, 120.5, 117.5, 113.5, 111.0, 70.9, 59.7, 56.0, 44.9, 37.3. HRMS (ESI-TOF) m/z: $[M + H]^+$Calcd for $C_{18}H_{19}N_2O_2$ - 295.1441, Found -295.1411; IR 3308.93, 2917.17, 1673.91, 1608.89.

2'-(2,4,5-trimethoxyphenyl)spiro[indoline-3,3'-pyrrolidin]-2-one (**5i**)

Following the general procedure tryptamine (200 mg, 1.25 mmol), 2, 4, 5-trimethoxybenzaldehyde (245 mg, mmol), 1:1 THF/water (20 mL) with catalytic TFA and NBS (197.5 mg, 1.37 mmol) provided the desired compound **5i** in 325 mg (yield 73%) as white semi solid. ^{1}H NMR (500 MHz; DMSO-d_6): 9.82 (s, 1H); 7.56 (d, *J* = 5 Hz, 1H); 7.36 (d, *J*= 10 Hz,1H); 7.16-7.13 (t, *J* = 10 Hz, 1H); 7.04-7.01 (t, , *J* = 10 Hz, 1H); 6.93 (s, 1H); 6.82 (s, 1H),4.34 (s, 1H); 3.73 (s, 6H), 3.66 (s, 3H), 3.20-3.18 (m, 1H); 2.95-2.93 (m, 1H); 2.79-2.76(m,

1H), 2.67-2.63 (m, 1H). ^{13}C NMR (125 MHz; DMSO-d_6):181.2, 157.9, 151.6, 150.5, 142.6, 136.6, 129.0, 124.7, 123.2, 119.3, 119.1, 113.7, 112.4, 71.4, 59.5, 56.1, 56.0, 55.8, 44.8, 37.0. HRMS (ESI-TOF) m/z: $[M + H]^+$Calcd for $C_{20}H_{23}N_2O_4$ - 355.1652, Found-355.1656; IR 3312.03, 1719.32, 1458.50, 1215.77.

2'-(2-fluoro-4-methoxyphenyl)spiro[indoline-3,3'-pyrrolidin]-2-one (**5j**)

Following the general procedure tryptamine (200 mg, 1.25 mmol), 2-fluoro-4-methoxybenzaldehyde (192.5 mg, 1.25 mmol), 1:1 THF/water (20 mL) with catalytic TFA and NBS (197.5 mg, 1.37 mmol) provided the desired compound **5j** in 301 mg (yield 77%) as yellow liquid. ^{1}H NMR (500 MHz; DMSO-d_6): 10.18 (s, 1H); 7.42 (d, *J* = 10 Hz, 1H); 7.22 (d, *J* = 10 Hz,1H); 7.03-7.00 (t, *J* = 10 Hz, 1H); 6.97-6.94. (t, , *J* = 5 Hz, 1H); 6.89-6.85 (m, 2H); 6.68 (d, *J* = 10 Hz ,1H), 4.69 (s, 1H); 3.75 (s, 3H), 3.03-3.00 (m, 1H); 2.94-2.92 (m, 1H); 2.75-2.72 (m, 1H), 2.68-2.64 (m, 1H). ^{13}C NMR (125 MHz; DMSO-d_6): 181.6, 161.9, 160.0, 135.9, 134.4, 130.6, 126.8, 121.8, 120.6, 118.2, 117.5, 111.0, 108.9, 64.5, 55.6, 46.4, 37.7, 29.7. HRMS (ESI-TOF) m/z: $[M + H]^+$Calcd for $C_{18}H_{18}FN_2O_2$ - 313.1347, Found - 313.1317; IR 3217.99, 2923.44, 1673.98, 1503.47.

2'-(4-methoxy-3-methylphenyl)spiro[indoline-3,3'-pyrrolidin]-2-one (**5k**)

Following the general procedure tryptamine (200 mg, 1.25 mmol), 4-methoxy-3-methylbenzaldehyde (187.5 mg, 1.25 mmol), with catalytic TFA and NBS (158 mg, 1.1 mmol) in water (10 mL) provided the desired compound **5k** in 342 mg (yield 88%) as white semi solid. ^{1}H NMR (500 MHz; DMSO-d_6): 9.83, (s, 1H); 7.46 (d, *J* = 5 Hz, 1H); 7.25 (d, *J*= 10 Hz,1H); 7.14-7.12 (m, 2H); 7.06-7.03. (t, *J* = 5 Hz, 1H); 7.00 (d, *J* = 5 Hz, 1H); 6.97-6.94 (m, 1H), 4.37 (s, 1H); 3.79 (s, 3H), 3.26-3.24 (m, 1H); 3.14-3.12 (m, 1H); 2.91-2.90 (m, 1H), 2.83-2.79 (m, 1H), 2.13 (s, 3H). ^{13}C NMR (125 MHz; DMSO-d_6): 181.2, 157.4, 136.2, 132.7, 131.0, 127.9, 126.4, 125.4, 121.1, 118.5, 117.8, 111.3, 110.1, 71.2, 59.7, 55.6, 44.9, 37.2, 20.4. HRMS (ESI-TOF) m/z: $[M + H]^+$Calcd for $C_{19}H_{21}N_2O_2$ – 309.1598, Found -309.1608.

2'-phenylspiro[indoline-3,3'-pyrrolidin]-2-one (**5l**)

Following the general procedure tryptamine (200 mg, 1.25 mmol), benzaldehyde (132.5 mg, 1.25 mmol) 1:1 THF/water (20 mL) with catalytic TFA and NBS (197.5 mg, 1.37 mmol) provided the desired compound **5l** in 311 mg (yield 94%) as orange liquid. ^{1}H NMR (500 MHz; DMSO-d_6):

9.99 (s, 1H); 7.50 (d, *J* = 10 Hz,1H); 7.44-7.36 (m, 5H); 7.31 (d, *J* = 5 Hz, 1H); 7.11-7.07 (t, *J* = 10 Hz, 1H); 7.04-7.01 (t, *J* = 5 Hz, 1H); 4.48 (s, 1H); 3.29-3.26 (m, 1H); 3.22-3.18 (m, 1H); 2.98-2.93 (m, 1H), 2.90-2.85 (m, 1H). ^{13}C NMR (125 MHz; DMSO-d$_6$): 181.3, 139.0, 136.3, 131.9, 129.2, 128.5, 128.1, 126.3, 121.3, 118.7, 111.4, 71.0, 59.4, 45.1, 37.1. HRMS (ESI-TOF) m/z: [M + H]$^+$Calcd for $C_{17}H_{17}N_2O$- 265.1335,Found - 265.1312;IR 2993.23, 2559.81, 1667.05, 1433.91.

2'-(2,5-bis(trifluoromethyl)phenyl)spiro[indoline-3,3'-pyrrolidin]-2-one (**5m**)

Following the general procedure tryptamine (200 mg, 1.25 mmol), 2, 5-bistrifluoromethylbenzaldehyde (302.5 mg, 1.25 mmol) with catalytic TFA and NBS (158 mg, 1.1 mmol) in water (10 mL) provided the desired compound **5m** in 225 mg (yield 45%) as viscous liquid.^{1}H NMR (500 MHz; DMSO-d$_6$): 10.28 (s, 1H); 8.06 (s, 1H); 7.98 (s, 2H); 7.45 (d, *J* = 10 Hz, 1H); 7.26 (d, *J* = 5 Hz, 1H); 7.06-7.03 (t, *J* = 10 Hz, 1H); 7.00-6.97 (t, *J* = 10 Hz, 1H); 4.67 (s, 1H); 3.09-3.06 (m, 1H); 3.01-2.97 (m, 1H); 2.81-2.77 (m, 1H), 2.71-2.68 (m, 1H). ^{13}C NMR (125 MHz; DMSO-d$_6$): 177.2, 146.6, 136.1, 133.8, 129.9, 129.2, 126.7, 124.5, 122.4, 120.9, 118.5, 117.8, 111.1, 69.9, 56.8, 43.7, 35.2. HRMS (ESI-TOF) m/z: [M + H]$^+$Calcd for $C_{19}H_{15}F_6N_2O$ - 401.1087, Found -401.1084; IR 3168.91, 2932.39, 1673.80, 1452.21.

1-(p-tolyl)-2,3,4,4a,9,9a-hexahydro-1H-pyrido[3,4-b]indole (**4a**)

Following the general procedure tryptamine (50 mg, 0.31 mmol), *p*-tolualdehyde (38 mg, 0.31 mmol) 1:1 THF/water (7 mL) with catalytic TFA and NBS (50 mg, 0.34 mmol) provided the desired compound **4a** in 31 mg (yield 39%) as colorless solid in 4 hours time.^{1}H NMR (500 MHz; DMSO-d$_6$): 10.42 (s, 1H); 7.44 (d, *J* =8 Hz, 1H); 7.26 (d, *J* = 10 Hz, 1H); 7.21-7.13 (m, 4H); 7.04-7.03 (m, 1H); 7.01-6.96 (m, 1H); 5.09 (s, 1H); 3.11-3.08 (m, 1H); 2.98-2.93 (m, 1H); 2.76-2.75 (m, 1H); 2.71-2.69 (m, 1H); 2.32 (s, 3H). ^{13}C NMR (125 MHz; DMSO-d$_6$): 140.6, 136.8, 136.5, 135.9, 129.2, 128.9, 127.4, 120.9, 118.7, 118.01, 111.6, 108.6, 56.9, 41.8, 22.7, 21.3. HRMS (ESI-TOF) m/z: [M + H]$^+$Calcd for $C_{18}H_{19}N_2$ – 263.2543, Found - 263.1531.

1. Screening of compounds 5a-m against MCF-7 cells (48h)

	1	2	3	4	5	6	7	8	9	10	11	12
A	1.413	1.521	1.712	0.037	1.433	1.062	0.976	0.036	0.033	0.037	0.036	0.038
B	0.479	0.312	0.512	0.037	0.991	1.108	0.759	0.036	0.036	0.039	0.036	0.042
C	0.904	1.084	1.12	0.046	0.982	0.51	0.802	0.036	0.037	0.037	0.037	0.039
D	1.123	0.912	1.01	0.037	0.712	0.782	0.659	0.042	0.037	0.036	0.037	0.038
E	1.102	0.924	0.991	0.037	0.787	0.97	1.09	0.037	0.037	0.037	0.038	0.039
F	0.987	0.936	0.667	0.037	0.784	0.84	0.964	0.037	0.035	0.038	0.038	0.037
G	0.613	0.712	0.781	0.038	0.512	0.604	0.698	0.037	0.037	0.037	0.038	0.039
H	1.173	1.042	0.953	0.036	0.037	0.037	0.037	0.037	0.039	0.036	0.04	0.038

	O.D1	O.D2	O.D3	AVG	CTRL-TST	CTRL-TST	*100	stdev
control	1.413	1.521	1.712	1.548667	0	0	0	0.151408
eto	0.479	0.312	0.512	0.434333	1.114333	0.719544	71.95437	0.107221
**5b**	0.904	1.084	1.12	1.036	0.512667	0.331037	33.10375	0.115724
**5c**	1.123	0.912	1.01	1.015	0.533667	0.344598	34.45975	0.105589
**5a**	1.102	0.924	0.991	1.005667	0.543	0.350624	35.06242	0.089902
**5d**	0.987	0.936	0.667	0.863333	0.685333	0.442531	44.25312	0.171931
**5e**	0.613	0.712	0.781	0.702	0.846667	0.546707	54.67068	0.084445
**5m**	1.173	1.042	0.953	1.056	0.492667	0.318123	31.81231	0.110666
**5g**	1.433	1.062	0.976	1.157	0.391667	0.252906	25.29057	0.24286
**5f**	0.991	1.108	0.759	0.952667	0.596	0.384847	38.48472	0.17763
**5h**	0.982	0.51	0.802	0.764667	0.784	0.506242	50.62419	0.238204
**5i**	0.712	0.782	0.659	0.717667	0.831	0.536591	53.65906	0.061695
**5j**	0.787	0.97	1.09	0.949	0.599667	0.387215	38.72148	0.152588
**5k**	0.784	0.84	0.964	0.862667	0.686	0.442962	44.29617	0.092116
**5l**	0.512	0.604	0.698	0.604667	0.944	0.609557	60.95566	0.093002

2. Screening of compound 5a-m against COS-7 cells (48h)

	1	2	3	4	5	6	7	8	9	10	11	12
A	1.213	1.412	1.312	0.037	1.492	1.312	1.039	0.039	0.038	0.038	0.036	0.036
B	0.212	0.268	0.4	0.039	0.712	0.815	1.037	0.039	0.039	0.04	0.038	0.038
C	1.109	0.912	1.1	0.043	0.925	0.982	1.038	0.043	0.04	0.04	0.04	0.043
D	1.072	0.813	0.918	0.049	1.07	1.21	1.043	0.036	0.039	0.039	0.039	0.04
E	1.21	1.05	1.215	0.038	1.31	1.289	1.042	0.039	0.038	0.037	0.037	0.045
F	1.051	1.271	1.252	0.044	1.201	1.412	1.043	0.037	0.037	0.04	0.044	0.042
G	1.356	1.398	1.043	0.053	1.31	0.981	1.039	0.038	0.038	0.041	0.04	0.043
H	1.091	1.217	1.649	0.042	0.042	0.038	0.036	0.04	0.036	0.041	0.038	0.047

	O.D1	O.D2	O.D3	AVG	CTRL-TES	CTRL-TEST/CTRL	*100	stdev
control	1.213	1.412	1.312	1.312333	0	0	0	0.0995
eto	0.212	0.268	0.4	0.293333	1.019	0.776479553	77.64796	0.096526
**5b**	1.109	0.912	1.1	1.040333	0.272	0.207264415	20.72644	0.111231
**5c**	1.072	0.813	0.918	0.934333	0.378	0.288036576	28.80366	0.13027
**5a**	1.21	1.05	1.215	1.158333	0.154	0.117348235	11.73482	0.093853
**5d**	1.051	1.271	1.252	1.191333	0.121	0.092202184	9.220218	0.121903
**5e**	1.356	1.398	1.043	1.265667	0.046667	0.035560071	3.556007	0.193975
**5n**	1.091	1.217	1.649	1.319	-0.00667	-0.00508001	-0.508	0.29265
**5g**	1.492	1.312	1.039	1.281	0.031333	0.023876048	2.387605	0.228086
**5f**	0.712	0.815	1.037	0.854667	0.457667	0.348742697	34.87427	0.166091
**5h**	0.925	0.982	1.038	0.981667	0.330667	0.251968504	25.19685	0.056501
**5i**	1.07	1.21	1.043	1.107667	0.204667	0.155956312	15.59563	0.089646
**5j**	1.31	1.289	1.042	1.213667	0.098667	0.07518415	7.518415	0.149038
**5k**	1.201	1.412	1.043	1.218667	0.093667	0.071374143	7.137414	0.185133
**5l**	1.31	0.981	1.039	1.11	0.202333	0.154178308	15.41783	0.175616

3. EC_{50} of compounds 5e, 5i and 5l

	1	2	3	4	5	6	7	8	9	10	11	12
A	1.114	1.213	1.413	0.038	1.109	1.019	0.912	0.036	0.036	1.112	1.203	1.187
B	0.312	0.433	0.341	0.038	0.234	0.298	0.274	0.036	0.036	0.341	0.312	0.287
C	0.912	0.905	1.012	0.038	1.012	0.913	1.237	0.036	0.037	1.109	1.112	1.031
D	0.713	0.876	0.9	0.04	0.91	0.897	0.978	0.038	0.037	0.991	0.891	0.912
E	0.7	0.614	0.621	0.041	0.781	0.772	0.764	0.042	0.039	0.712	0.62	0.65
F	0.416	0.452	0.534	0.04	0.678	0.691	0.712	0.037	0.038	0.534	0.598	0.6
G	0.39	0.374	0.471	0.042	0.54	0.413	0.491	0.038	0.04	0.412	0.398	0.375
H	0.036	0.037	0.037	0.036	0.046	0.042	0.04	0.038	0.038	0.039	0.037	0.037

EC_{50} of **5l**

		O.D1	O.D2	O.D3	AVG	CTRL-TST	CTRL-TST	*100	stdev	
ctrl		1.114	1.213	1.413	1.246667	0	0	0	0.152317	
eto		0.312	0.433	0.341	0.362	0.884667	0.709626	70.96257	0.063174	
	10	0.912	0.905	1.012	0.943	0.303667	0.243583	24.35829	0.059858	
	20	0.713	0.876	0.9	0.829667	0.417	0.334492	33.4492	0.101746	EC_{50}=3.53 micromolar
	30	0.7	0.614	0.621	0.645	0.601667	0.48262	48.26203	0.04776	
	40	0.416	0.452	0.534	0.467333	0.779333	0.625134	62.51337	0.060476	
	50	0.39	0.374	0.471	0.411667	0.835	0.669786	66.97861	0.052003	

EC_{50} of **5e**

		O.D1	O.D2	O.D3	AVG	CTRL-TST	CTRL-TST	*100	stdev	
ctrl		1.109	1.019	0.912	1.013333	0	0	0	0.098622	
eto		0.234	0.298	0.274	0.268667	0.744667	0.734868	73.48684	0.032332	
	10	1.012	0.913	1.237	1.054	-0.04067	-0.04013	-4.01316	0.166033	
	20	0.91	0.897	0.978	0.928333	0.085	0.083882	8.388158	0.043501	EC_{50}=6 micromolar
	30	0.781	0.772	0.764	0.772333	0.241	0.237829	23.78289	0.008505	
	40	0.678	0.691	0.712	0.693667	0.319667	0.315461	31.54605	0.017156	
	50	0.54	0.413	0.491	0.481333	0.532	0.525	52.5	0.064049	

EC_{50} of **5i**

		O.D1	O.D2	O.D3	AVG	CTRL-TST	CTRL-TST	*100	stdev	
ctrl		1.112	1.203	1.187	1.167333	0	0	0	0.048583	
eto		0.341	0.312	0.287	0.313333	0.854	0.731582	73.1582	0.027025	
	10	1.109	1.112	1.031	1.084	0.083333	0.071388	7.138778	0.045924	
	20	0.991	0.891	0.912	0.931333	0.236	0.20217	20.21702	0.052729	
	30	0.712	0.62	0.65	0.660667	0.506667	0.434038	43.40377	0.046918	EC_{50}=4.01 micromolar
	40	0.534	0.598	0.6	0.577333	0.59	0.505425	50.54255	0.037541	
	50	0.412	0.398	0.375	0.395	0.772333	0.661622	66.16219	0.018682	

4. **DNA ladder assay of compounds, 5e, 5i and 5l (Figure 1):**

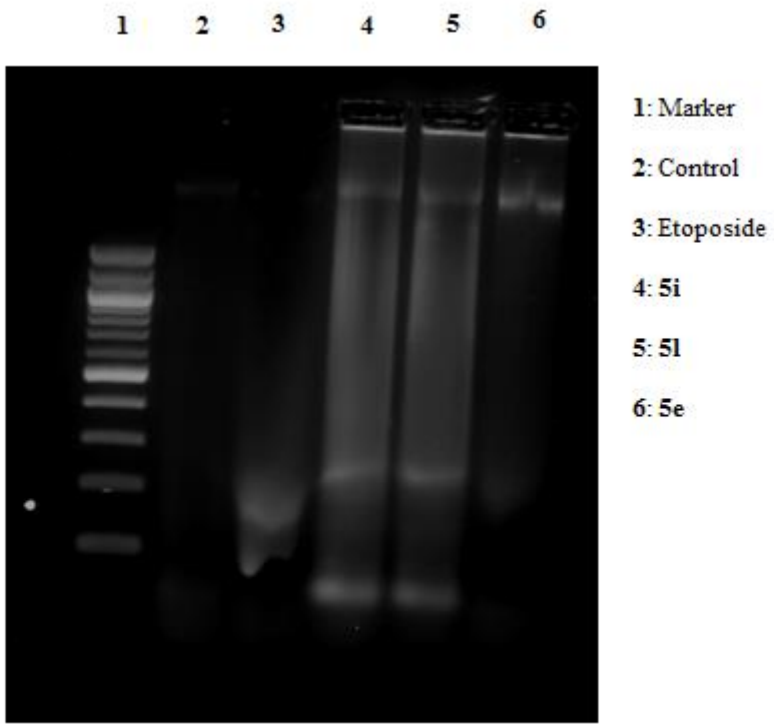

5. **Table 1. Various interactions between 5l and the target protein with the amino acid involve in the binding and the distances between the binding atoms.**

**Interaction Type**	**Residue**	**Amino Acid**	**Distance (Å)**
Hydrogen Bond	183	HIS	2.23
Hydrophobic Interaction	155	PHE	3.21
Hydrophobic Interaction	155	PHE	3.98
Hydrophobic Interaction	210	PHE	3.43
Hydrophobic Interaction	210	PHE	3.30
Hydrophobic Interaction	210	PHE	3.67
Hydrophobic Interaction	276	LEU	3.60
Pi-Stacking	209	TYR	6.70
Pi-Stacking	209	TYR	6.20

6. **Annexin-PI FACS experiments:**

Quadrants Q1: Necrosis (Ann -ve/PI +ve); Q2: Late apoptosis (Ann +ve/ PI +ve); Q3: Ann –ve/ PI –ve; Q4: Early Apoptosis (Ann +ve/ PI –ve).

a. **Figure 2 (Unstained)**

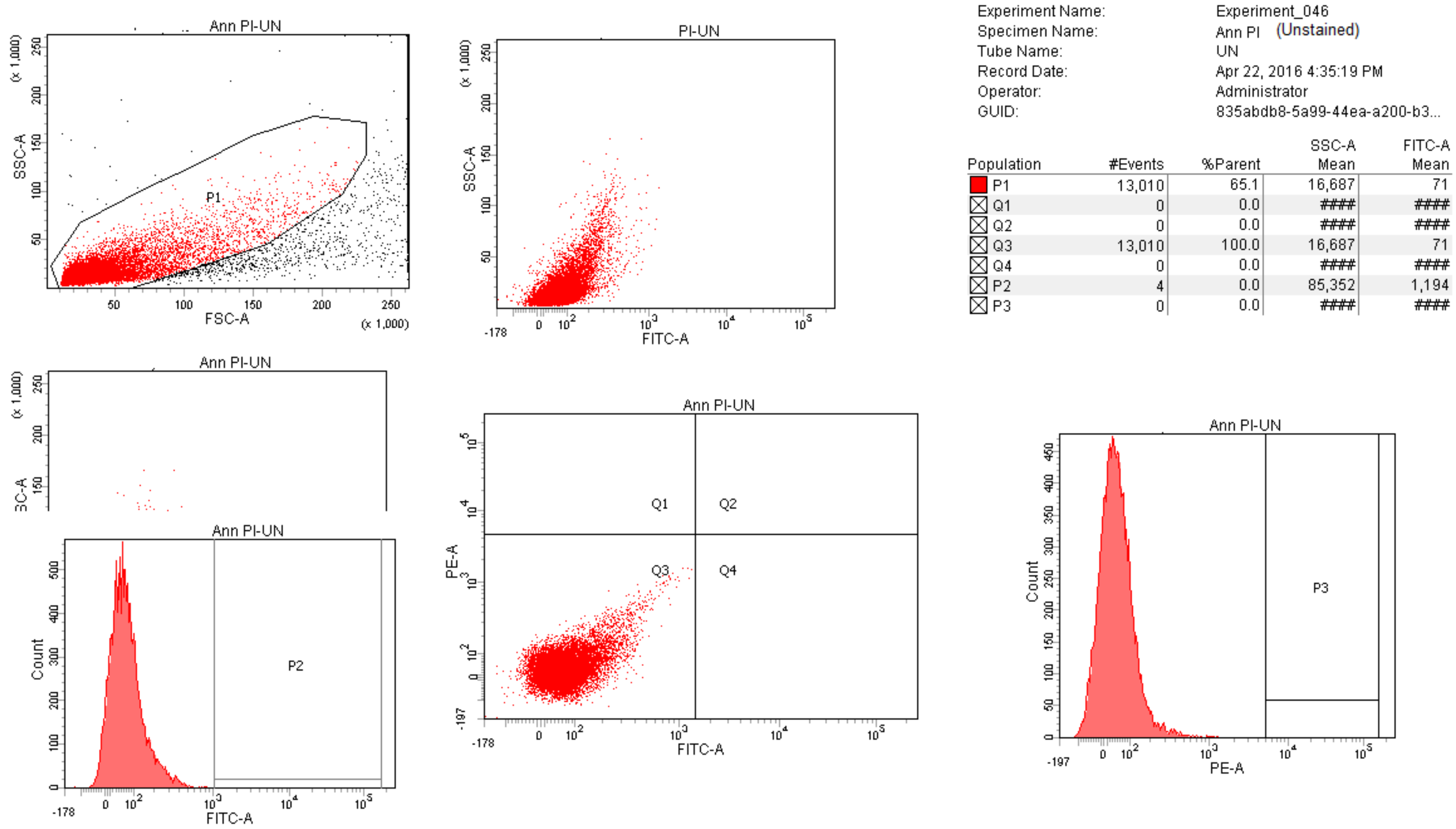

Population	#Events	%Parent	SSC-A Mean	FITC-A Mean
P1	13,010	65.1	16,687	71
Q1	0	0.0	####	####
Q2	0	0.0	####	####
Q3	13,010	100.0	16,687	71
Q4	0	0.0	####	####
P2	4	0.0	85,352	1,194
P3	0	0.0	####	####

b. Figure 3 (control @ 6h)

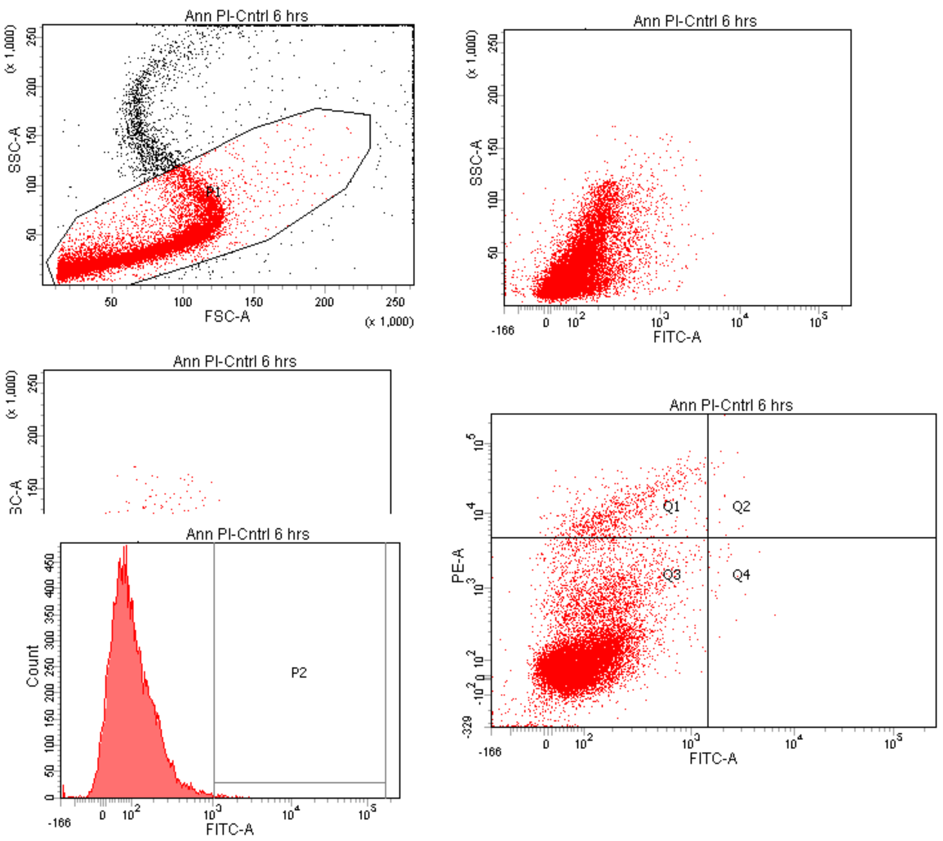

Experiment Name:	Experiment_046		
Specimen Name:	Ann PI		
Tube Name:	Cntrl 6 hrs		
Record Date:	Apr 22, 2016 4:37:07 PM		
Operator:	Administrator		
GUID:	25d43a36-0ade-4fd0-9ab8-95c...		

Population	#Events	%Parent	SSC-A Mean	FITC-A Mean
P1	14,871	74.4	34,965	118
Q1	812	5.5	40,746	296
Q2	16	0.1	96,267	2,109
Q3	14,021	94.3	34,520	102
Q4	22	0.1	61,239	2,289
P2	85	0.6	79,945	1,648
P3	763	5.1	42,853	335

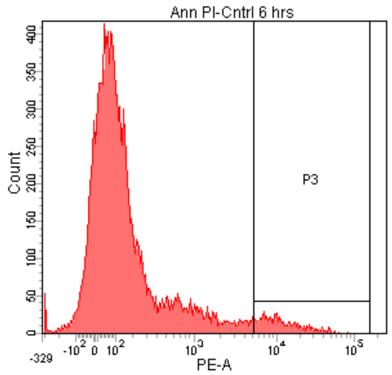

c. Figure 4 (control @ 12h)

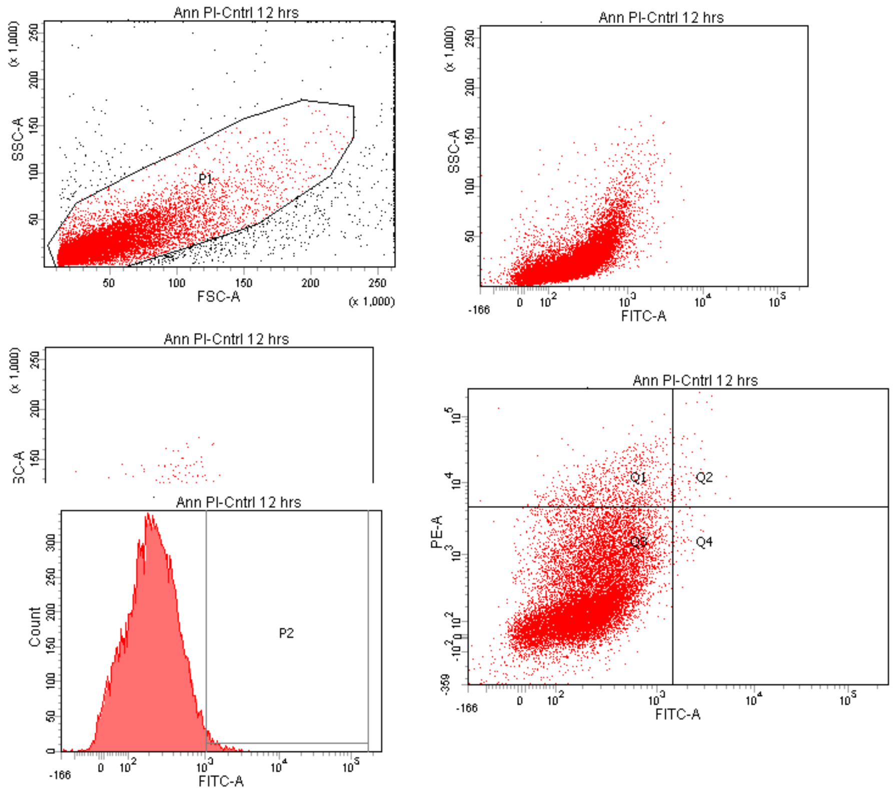

Experiment Name:	Experiment_046
Specimen Name:	Ann PI
Tube Name:	Cntrl 12 hrs
Record Date:	Apr 22, 2016 4:42:03 PM
Operator:	Administrator
GUID:	9816438c-e990-49f1-9fc4-5847...

Population	#Events	%Parent	SSC-A Mean	FITC-A Mean
P1	16,717	83.6	23,805	268
Q1	798	4.8	45,297	460
Q2	58	0.3	96,746	2,188
Q3	15,818	94.6	22,276	247
Q4	43	0.3	89,071	1,852
P2	234	1.4	85,133	1,560
P3	744	4.5	50,772	592

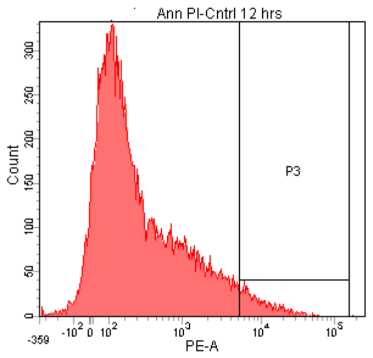

d. Figure 5 (control @ 24h)

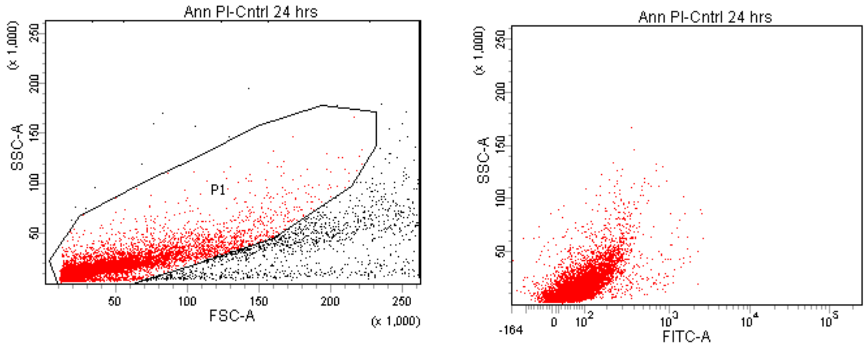

Experiment Name:	Experiment_046
Specimen Name:	Ann PI
Tube Name:	Cntrl 24 hrs
Record Date:	Apr 22, 2016 4:47:34 PM
Operator:	Administrator
GUID:	1416eb37-625d-4bf5-ac6e-015...

Population	#Events	%Parent	SSC-A Mean	FITC-A Mean
P1	8,769	43.8	17,359	87
Q1	1,451	16.5	35,531	138
Q2	7	0.1	57,741	1,867
Q3	7,303	83.3	13,661	74
Q4	8	0.1	61,649	1,809
P2	25	0.3	55,521	1,577
P3	1,221	13.9	36,083	133

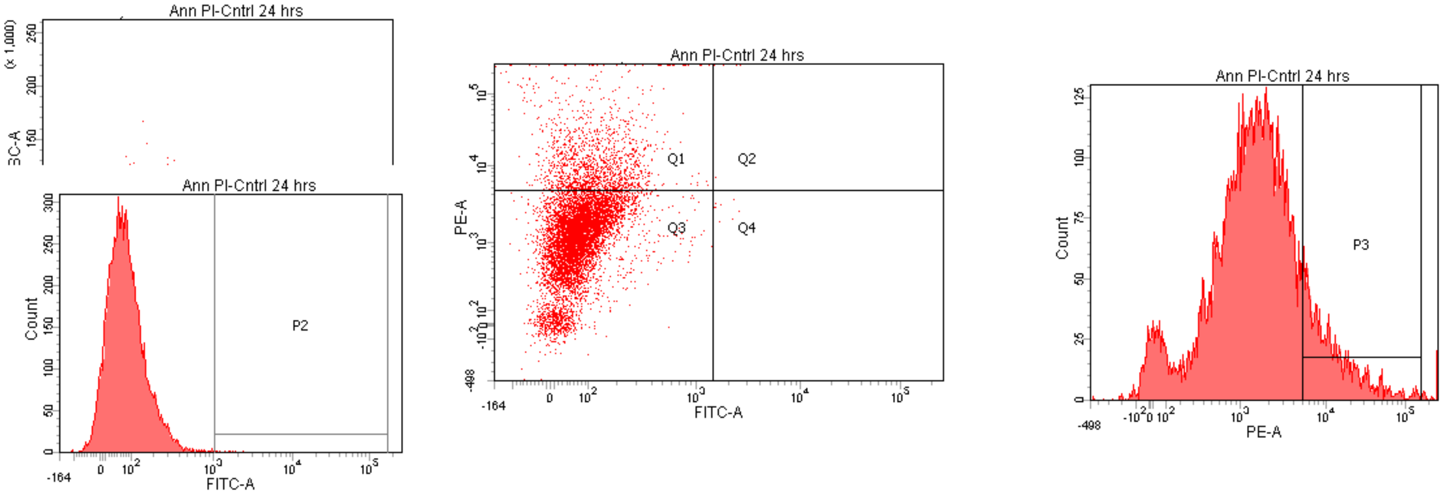

e. Figure 6 (Etoposide @ 6h)

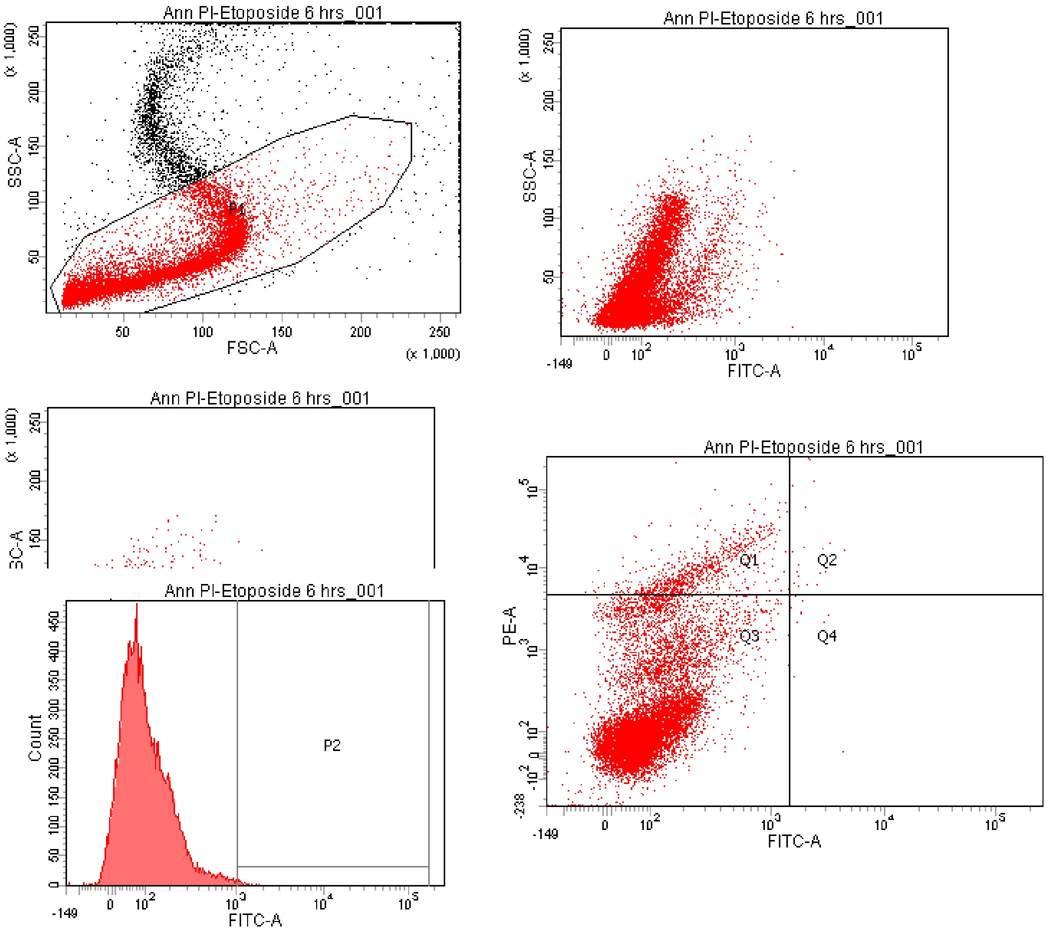

Experiment Name:	Experiment_046
Specimen Name:	Ann PI
Tube Name:	Etoposide 6 hrs_001
Record Date:	Apr 22, 2016 4:39:01 PM
Operator:	Administrator
GUID:	36cb3c5f-267e-4866-ad87-c59...

Population	#Events	%Parent	SSC-A Mean	FITC-A Mean
P1	14,043	70.2	39,121	116
Q1	957	6.8	45,349	311
Q2	19	0.1	86,915	2,222
Q3	13,056	93.0	38,563	96
Q4	11	0.1	77,919	2,126
P2	60	0.4	85,686	1,689
P3	833	5.9	48,986	375

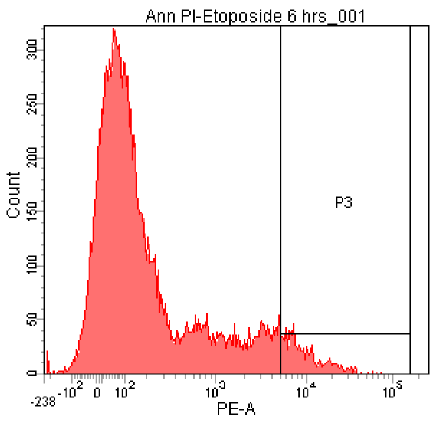

f. Figure 7 (Etoposide @ 12h)

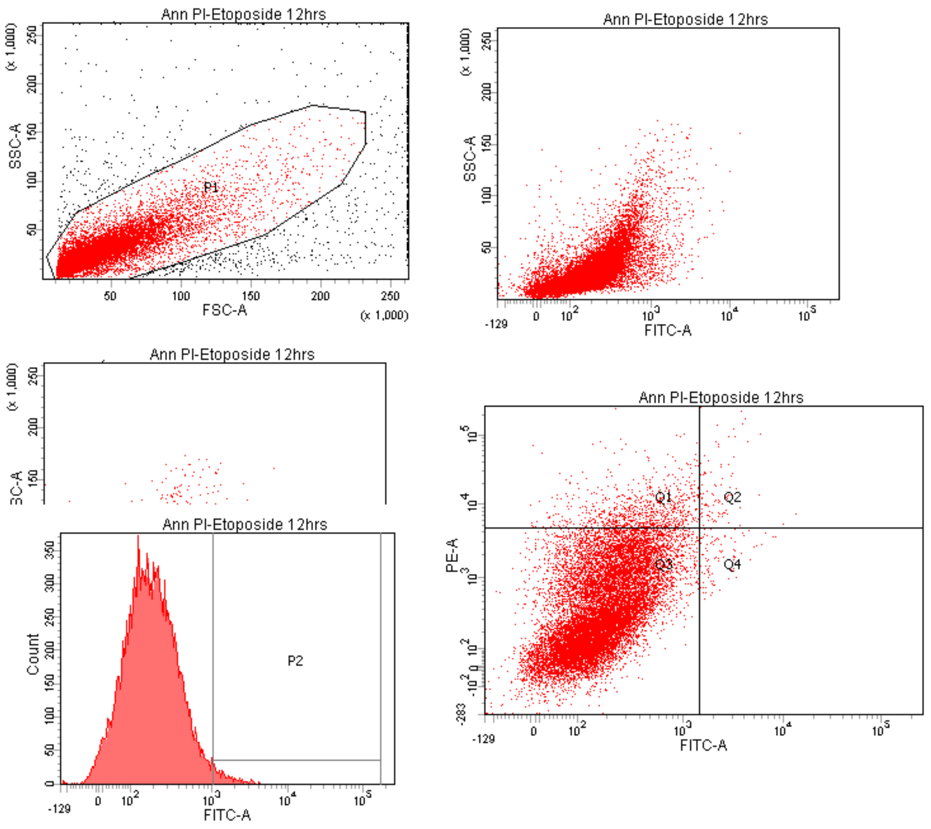

Experiment Name:	Experiment_046
Specimen Name:	Ann PI
Tube Name:	Etoposide 12hrs
Record Date:	Apr 22, 2016 4:43:28 PM
Operator:	Administrator
GUID:	329d8d82-649c-42f5-8814-9dc...

Population	#Events	%Parent	SSC-A Mean	FITC-A Mean
P1	16,597	83.0	27,003	249
Q1	853	5.1	47,501	496
Q2	92	0.6	90,366	2,469
Q3	15,561	93.8	25,283	210
Q4	91	0.5	64,919	2,455
P2	332	2.0	73,147	1,899
P3	776	4.7	52,909	733

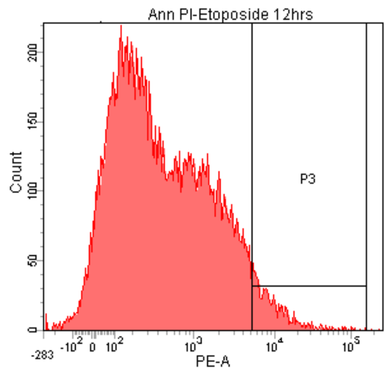

g. Figure 8 (Etoposide @ 24h)

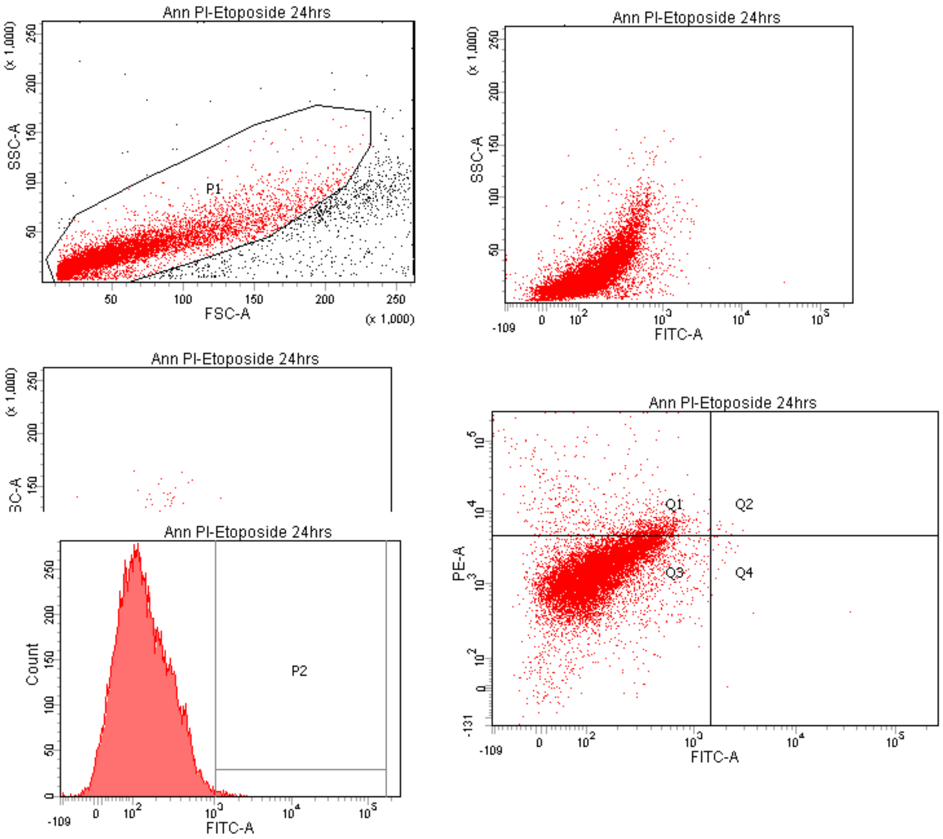

Experiment Name: Experiment_046
Specimen Name: Ann PI
Tube Name: Etoposide 24hrs
Record Date: Apr 22, 2016 4:49:51 PM
Operator: Administrator
GUID: 636aaedb-7c4d-433b-ba9f-0bc...

Population	#Events	%Parent	SSC-A Mean	FITC-A Mean
P1	12,613	63.1	25,875	175
Q1	1,083	8.6	59,358	335
Q2	10	0.1	89,530	1,932
Q3	11,500	91.2	22,608	152
Q4	20	0.2	59,158	3,663
P2	67	0.5	63,521	2,049
P3	787	6.2	58,871	340

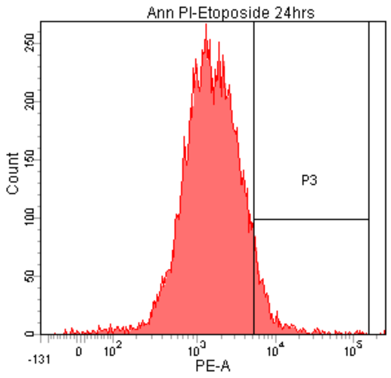

h. Figure 9 (Compound 5l @ 6h)

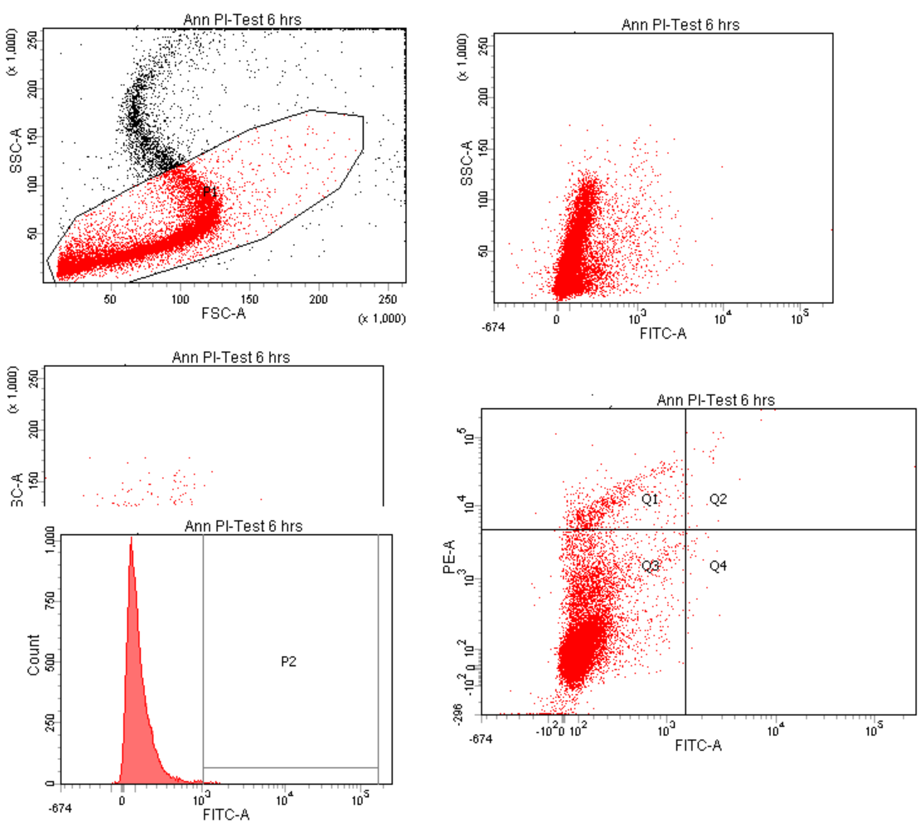

Experiment Name:	Experiment_046
Specimen Name:	Ann PI
Tube Name:	**5l @** 6 hrs
Record Date:	Apr 22, 2016 4:40:52 PM
Operator:	Administrator
GUID:	24413011-f7bd-44bd-be93-87e...

Population	#Events	%Parent	SSC-A Mean	FITC-A Mean
P1	14,763	73.8	39,729	155
Q1	740	5.0	37,427	326
Q2	32	0.2	77,624	10,835
Q3	13,966	94.6	39,697	118
Q4	25	0.2	77,252	1,787
P2	127	0.9	77,530	1,693
P3	705	4.8	39,810	781

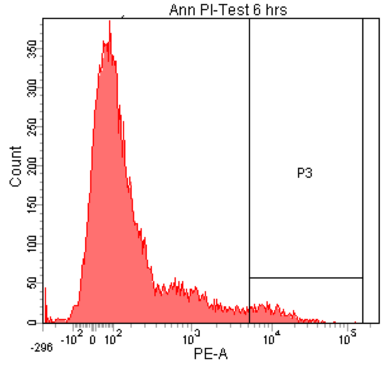

i. Figure 10 (Compound 5l @ 12h)

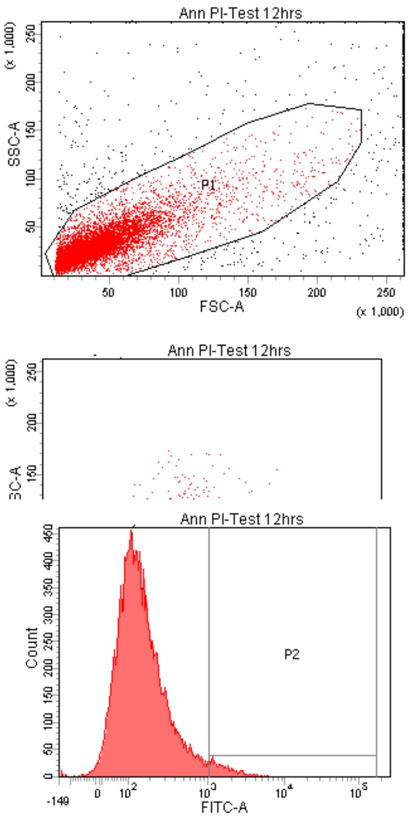

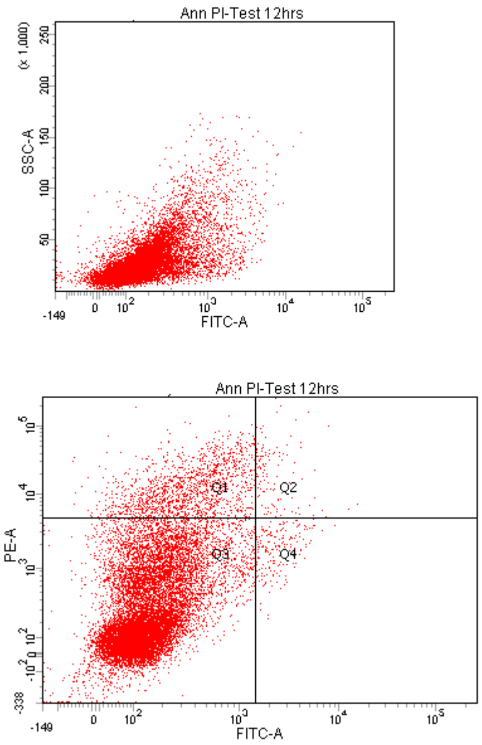

Experiment Name:	Experiment_046
Specimen Name:	Ann PI
Tube Name:	5l @12hrs
Record Date:	Apr 22, 2016 4:45:26 PM
Operator:	Administrator
GUID:	e35cd0d6-b249-40e8-a69b-9ee...

Population	#Events	%Parent	SSC-A Mean	FITC-A Mean
P1	16,634	83.2	25,108	231
Q1	1,476	8.9	40,651	419
Q2	128	0.8	95,647	2,649
Q3	14,835	89.2	22,623	162
Q4	195	1.2	50,198	2,506
P2	541	3.3	64,004	2,015
P3	1,476	8.9	45,637	591

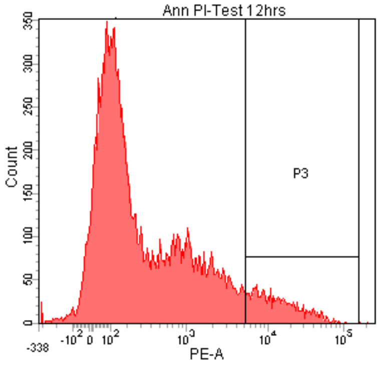

j. Figure 11 (Compound 5l @ 24h)

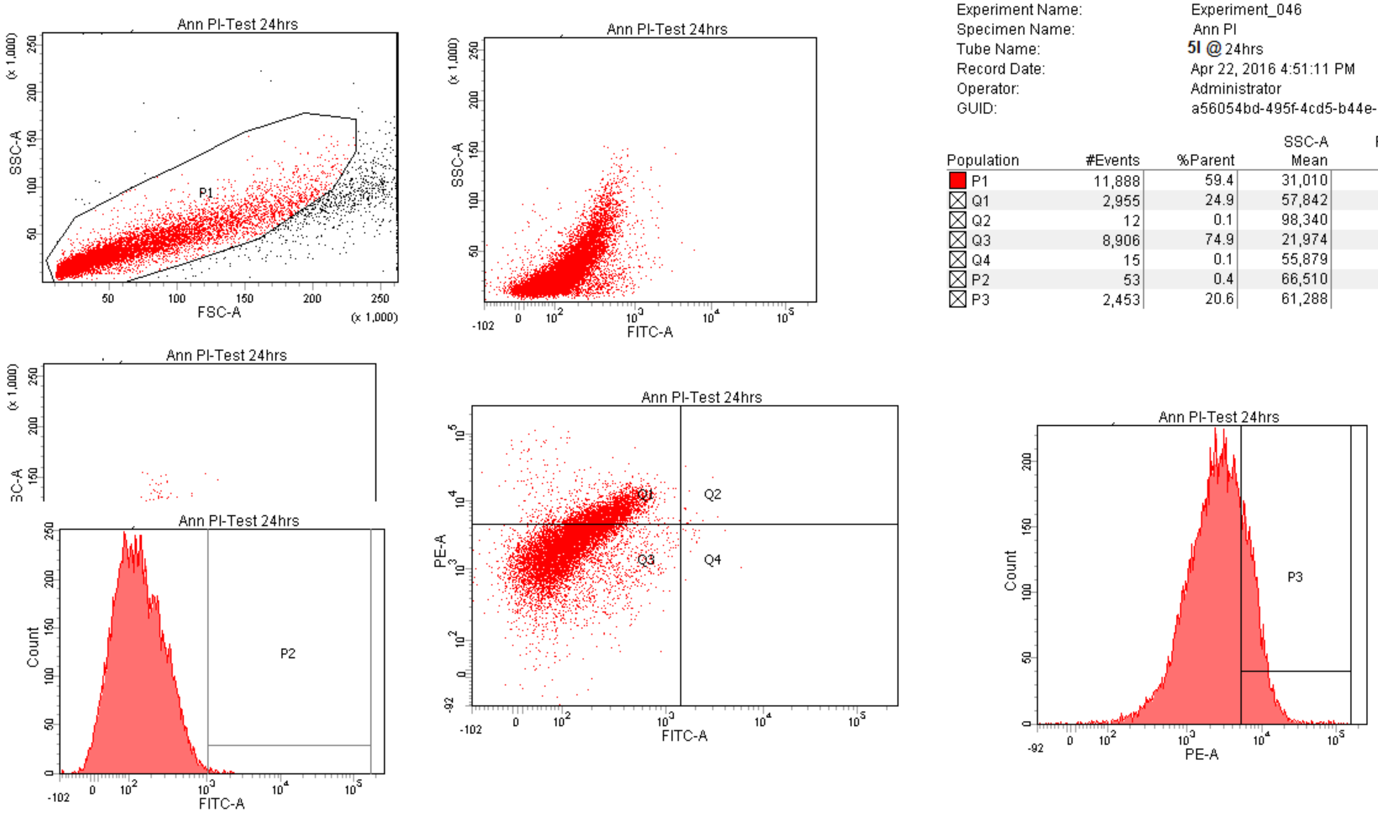

Experiment Name:	Experiment_046
Specimen Name:	Ann PI
Tube Name:	5l @ 24hrs
Record Date:	Apr 22, 2016 4:51:11 PM
Operator:	Administrator
GUID:	a56054bd-495f-4cd5-b44e-62f1...

Population	#Events	%Parent	SSC-A Mean	FITC-A Mean
P1	11,888	59.4	31,010	169
Q1	2,955	24.9	57,842	293
Q2	12	0.1	98,340	1,977
Q3	8,906	74.9	21,974	122
Q4	15	0.1	55,879	2,293
P2	53	0.4	66,510	1,677
P3	2,453	20.6	61,288	316

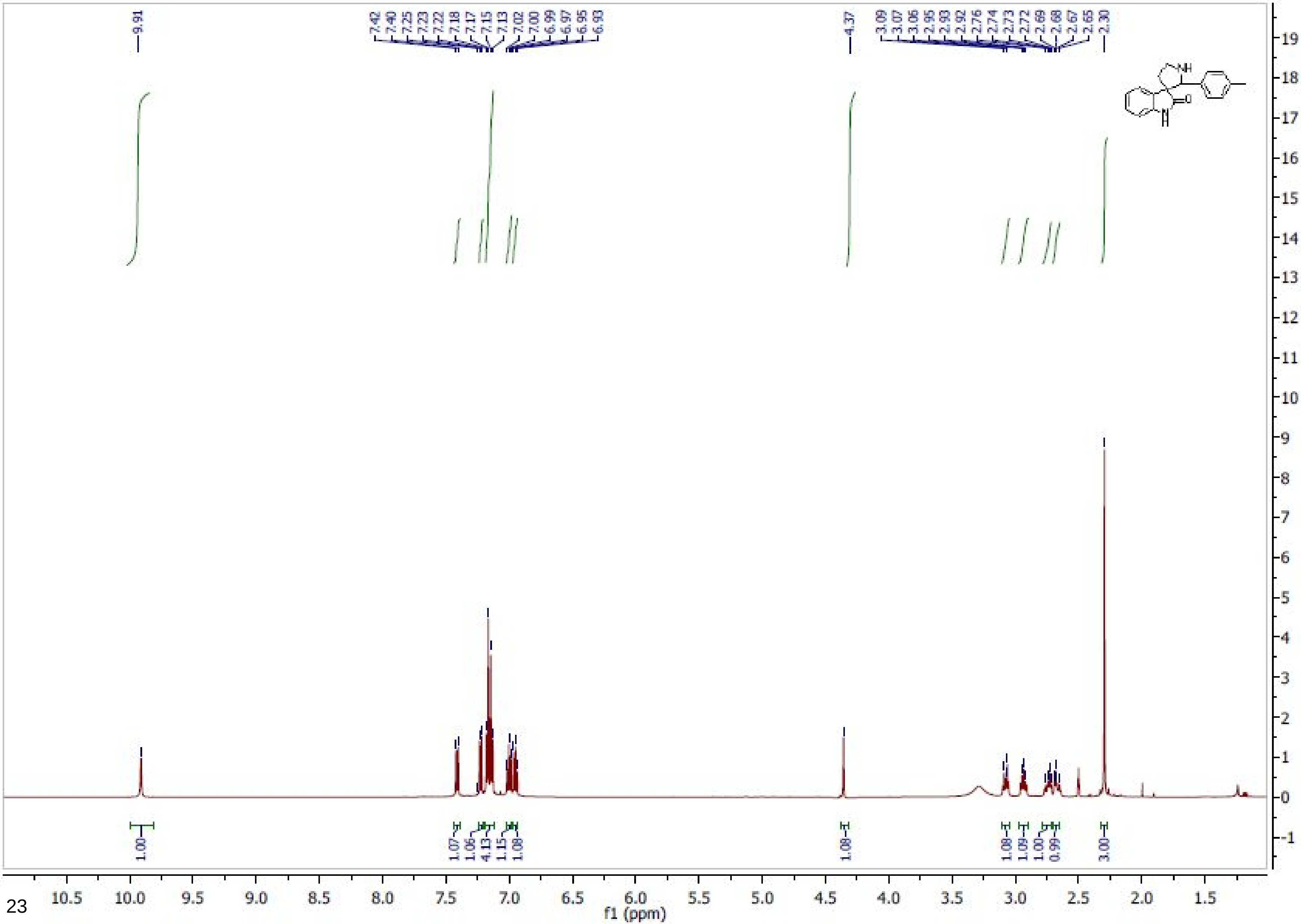

9.91
7.42
7.40
7.25
7.23
7.22
7.18
7.17
7.15
7.13
7.02
7.00
6.99
6.97
6.95
6.93
4.37
3.09
3.07
3.06
2.95
2.93
2.92
2.76
2.74
2.73
2.72
2.69
2.68
2.67
2.65
2.30
NH
O
N
H
1.00
1.07
1.06
4.13
1.15
1.08
1.08
1.08
1.09
1.00
0.99
3.00
f1 (ppm)

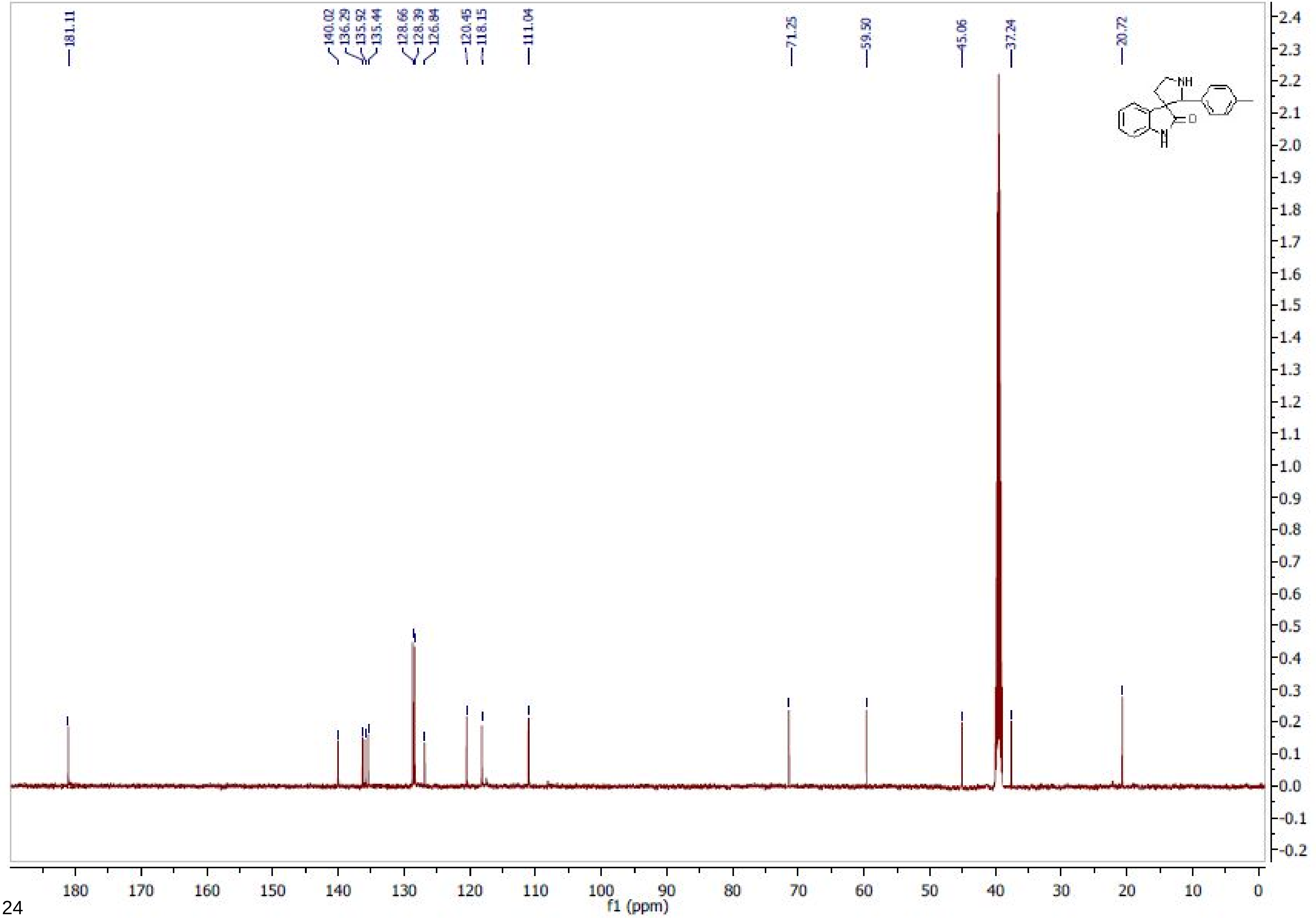

181.11
140.02
136.29
135.92
135.44
128.66
128.39
126.84
120.45
118.15
111.04
71.25
59.50
45.06
37.24
20.72
NH
O
N
H
f1 (ppm)

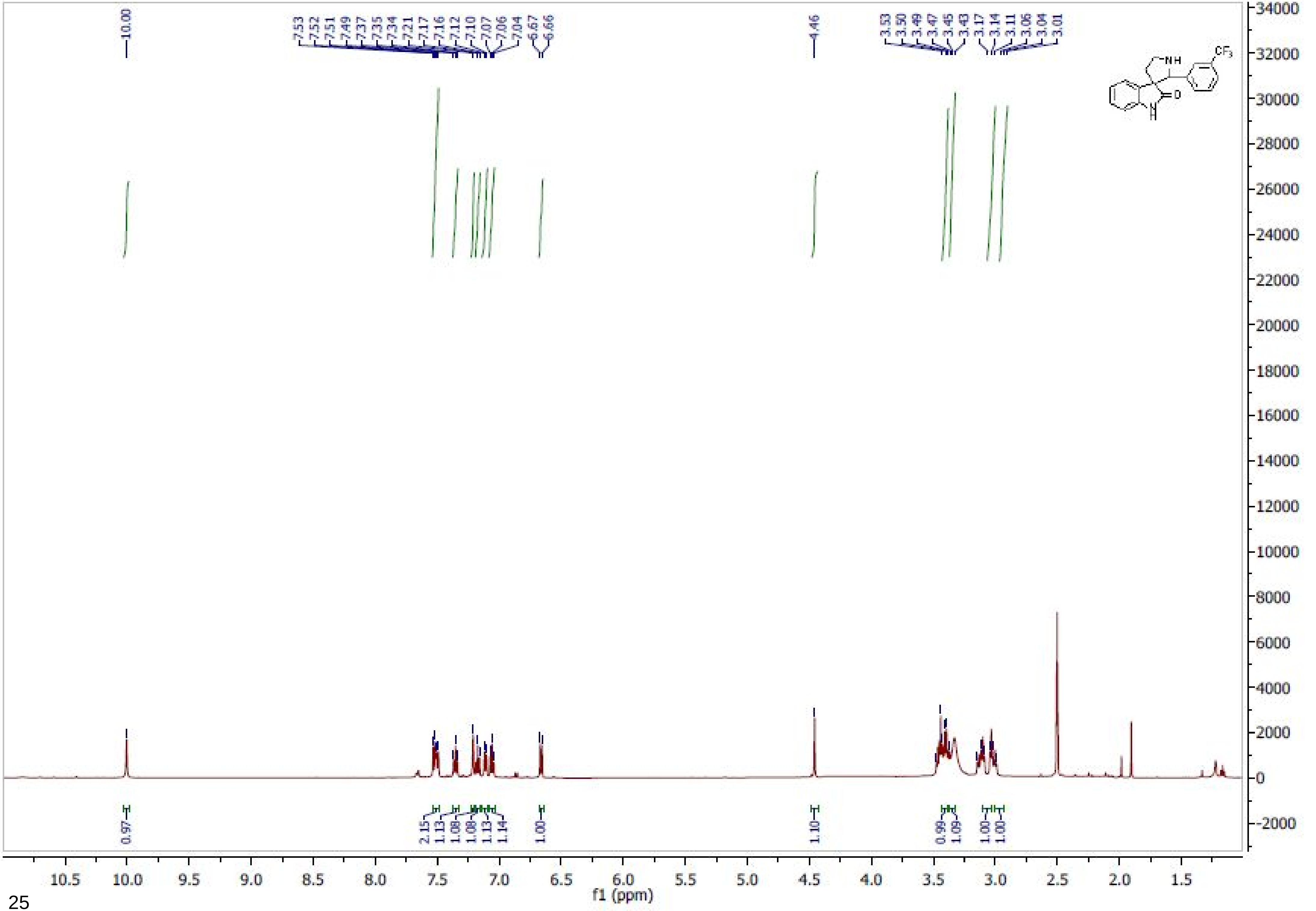

10.00
7.53
7.52
7.51
7.49
7.37
7.35
7.34
7.21
7.17
7.16
7.12
7.10
7.07
7.06
7.04
6.67
6.66
4.46
3.53
3.50
3.49
3.47
3.45
3.43
3.17
3.14
3.11
3.06
3.04
3.01
NH
CF3
O
H
N
0.97
2.15
1.13
1.08
1.08
1.13
1.14
1.00
1.10
0.99
1.09
1.00
1.00
f1 (ppm)

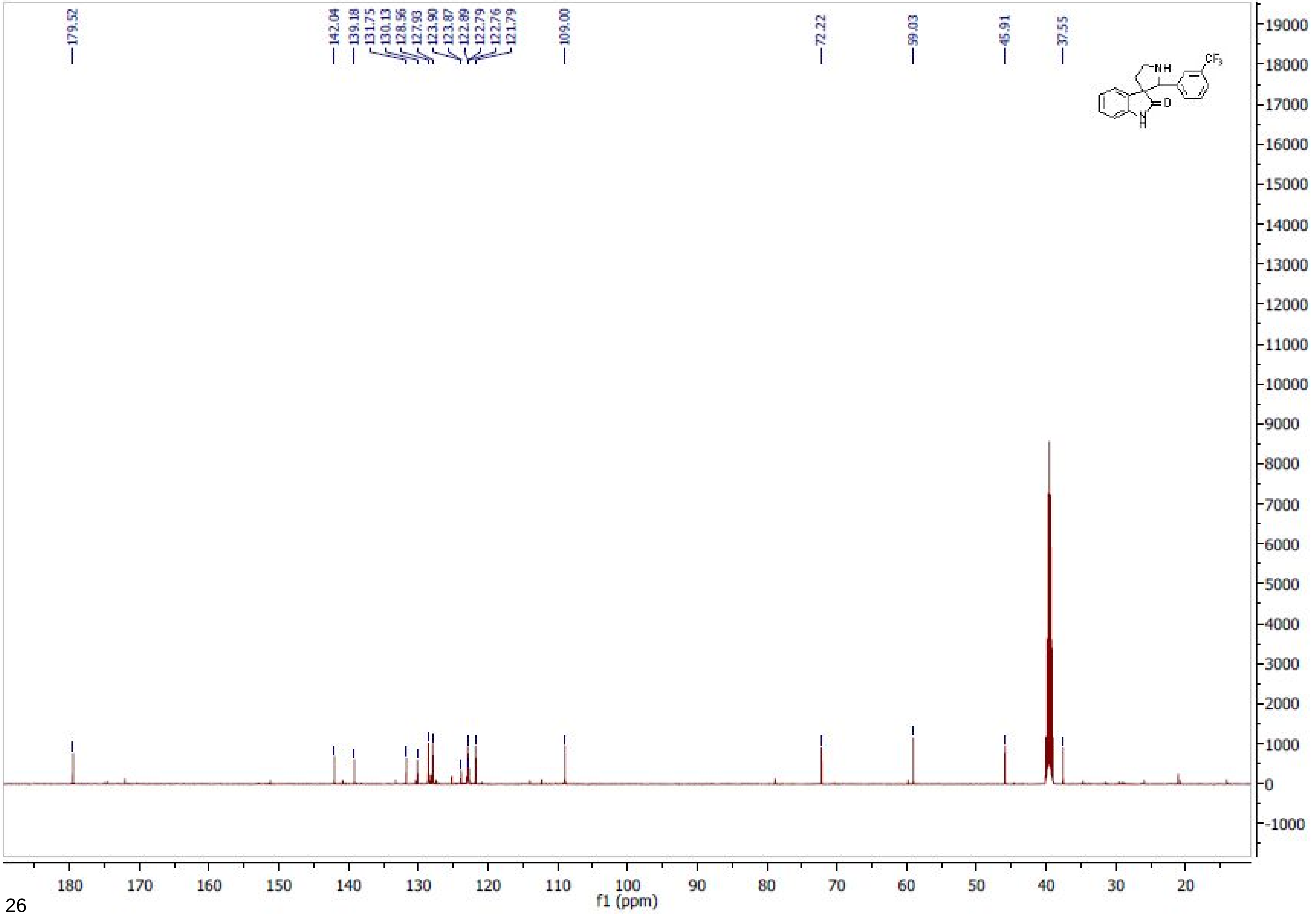
179.52
142.04
139.18
131.75
130.13
128.56
127.93
123.90
123.87
122.89
122.79
122.76
121.79
109.00
72.22
59.03
45.91
37.55
NH
CF3
O
H
f1 (ppm)

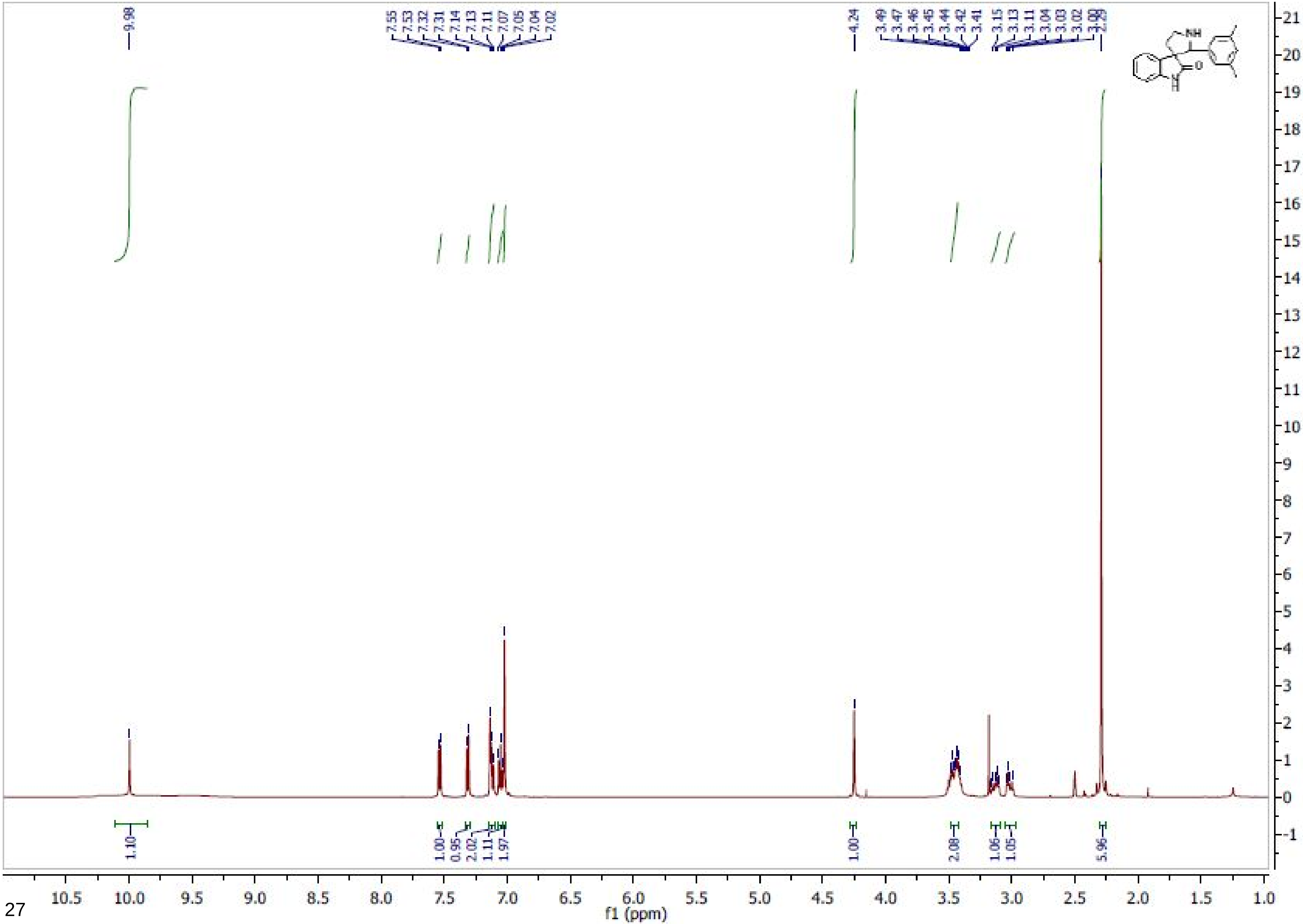

9.98
7.55
7.53
7.32
7.31
7.14
7.13
7.11
7.07
7.05
7.04
7.02
4.24
3.49
3.47
3.46
3.45
3.44
3.42
3.41
3.15
3.13
3.11
3.04
3.03
3.02
2.29
NH
O
N
H
1.10
1.00
0.95
2.02
1.11
1.97
1.00
2.08
1.06
1.05
5.96
f1 (ppm)

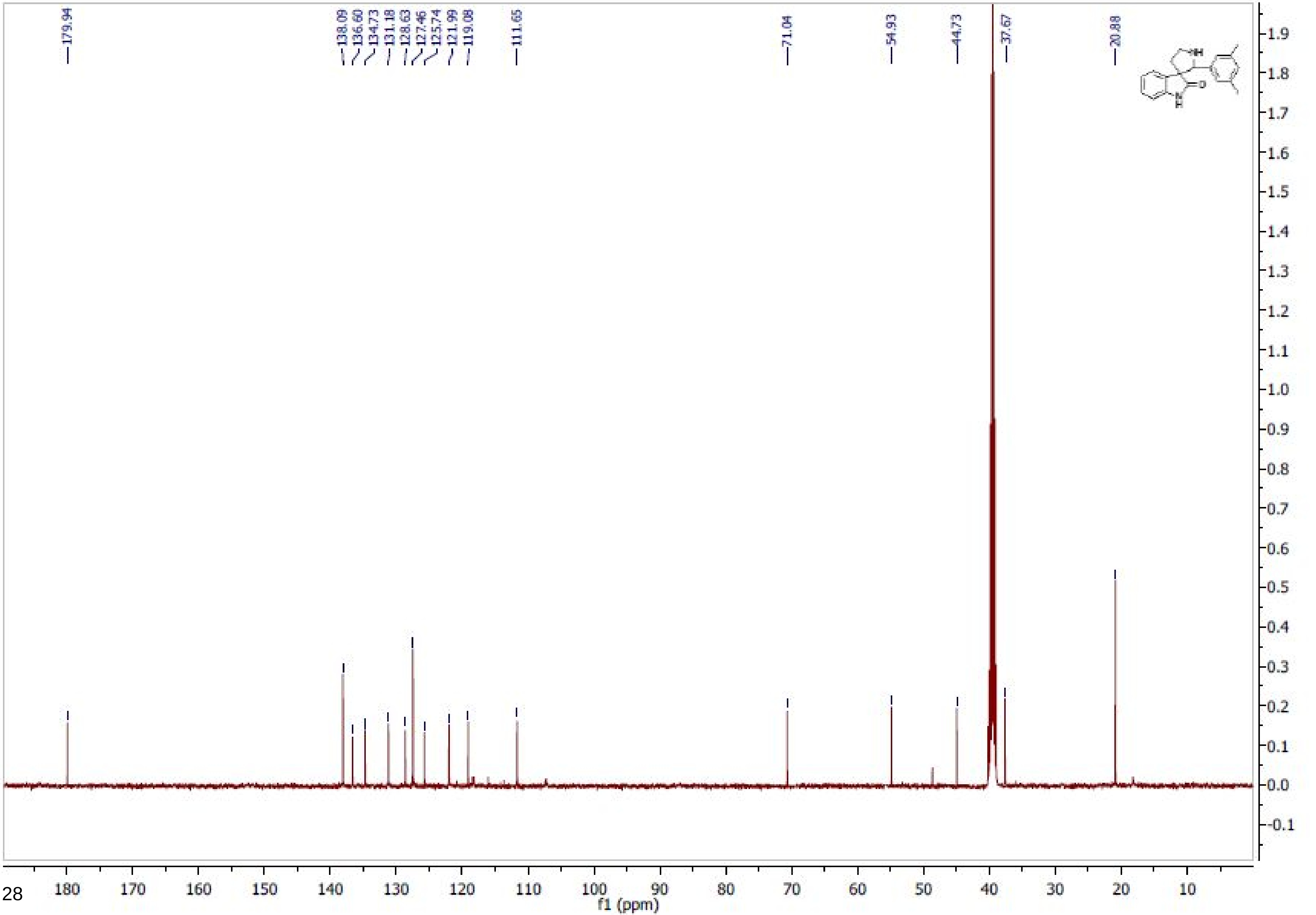
179.94
138.09
136.60
134.73
131.18
128.63
127.46
125.74
121.99
119.08
111.65
71.04
54.93
44.73
37.67
20.88
f1 (ppm)

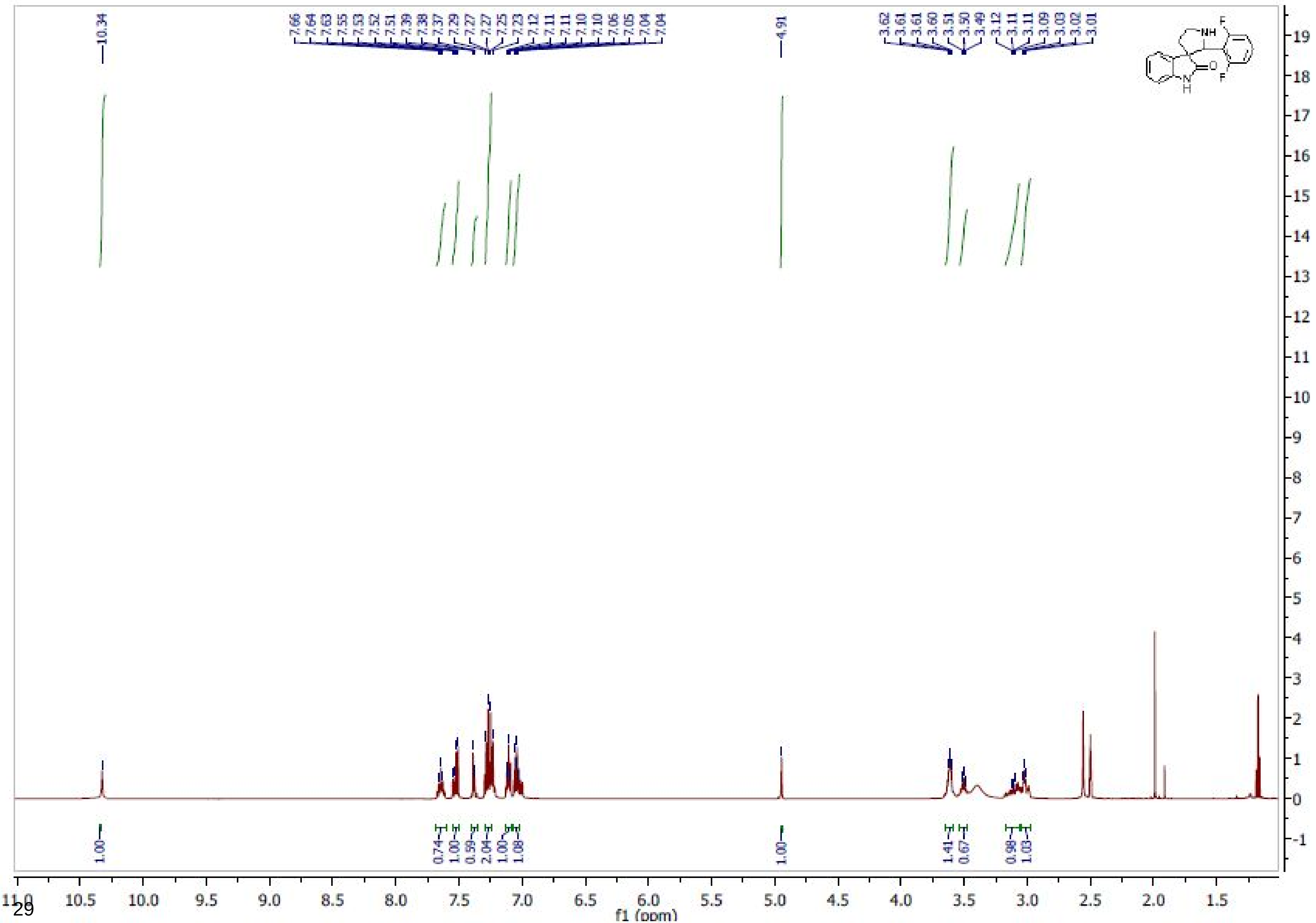

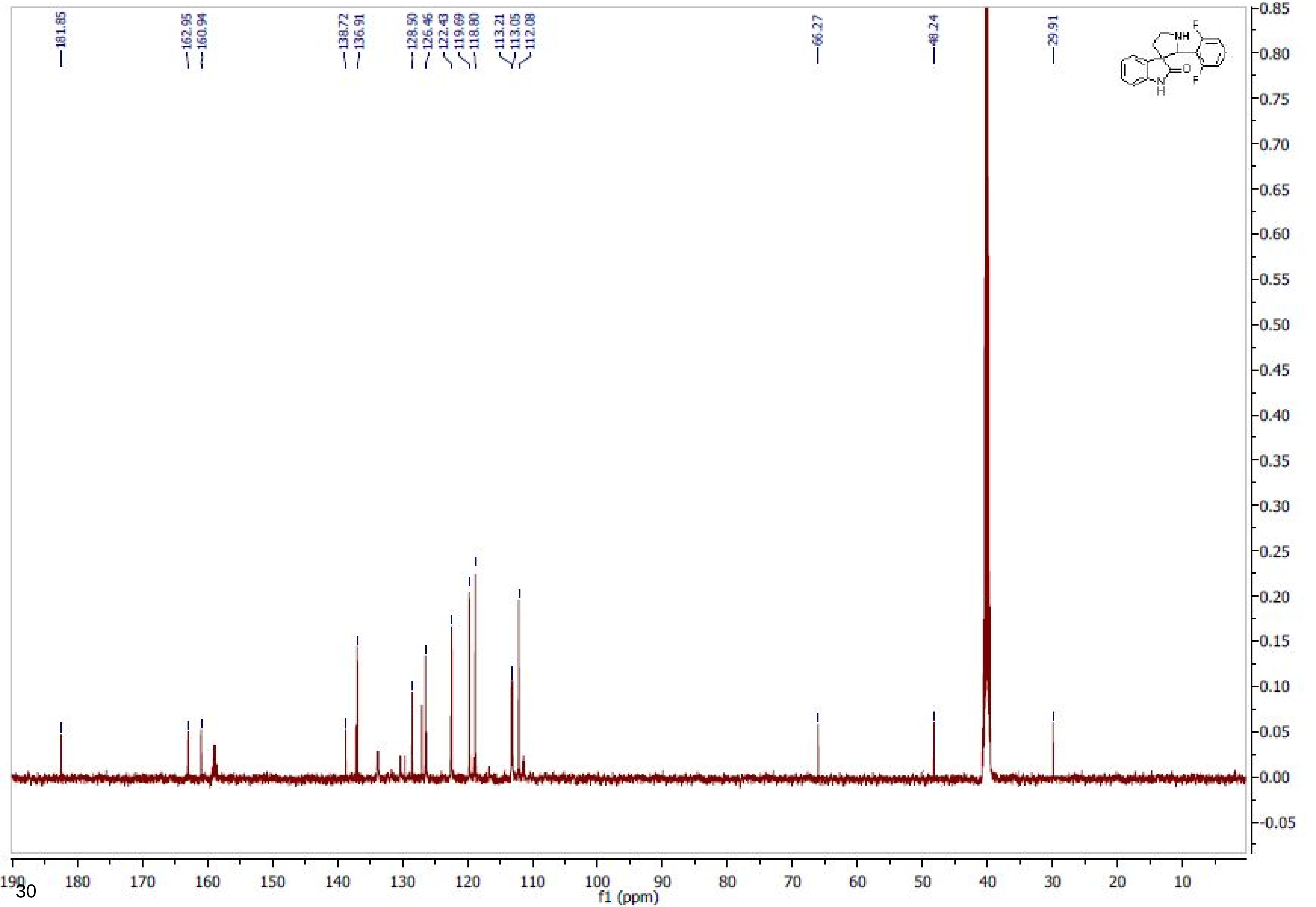
181.85
162.95
160.94
138.72
136.91
128.50
126.46
122.43
119.69
118.80
113.21
113.05
112.08
66.27
48.24
29.91
NH
F
F
O
N
H
f1 (ppm)

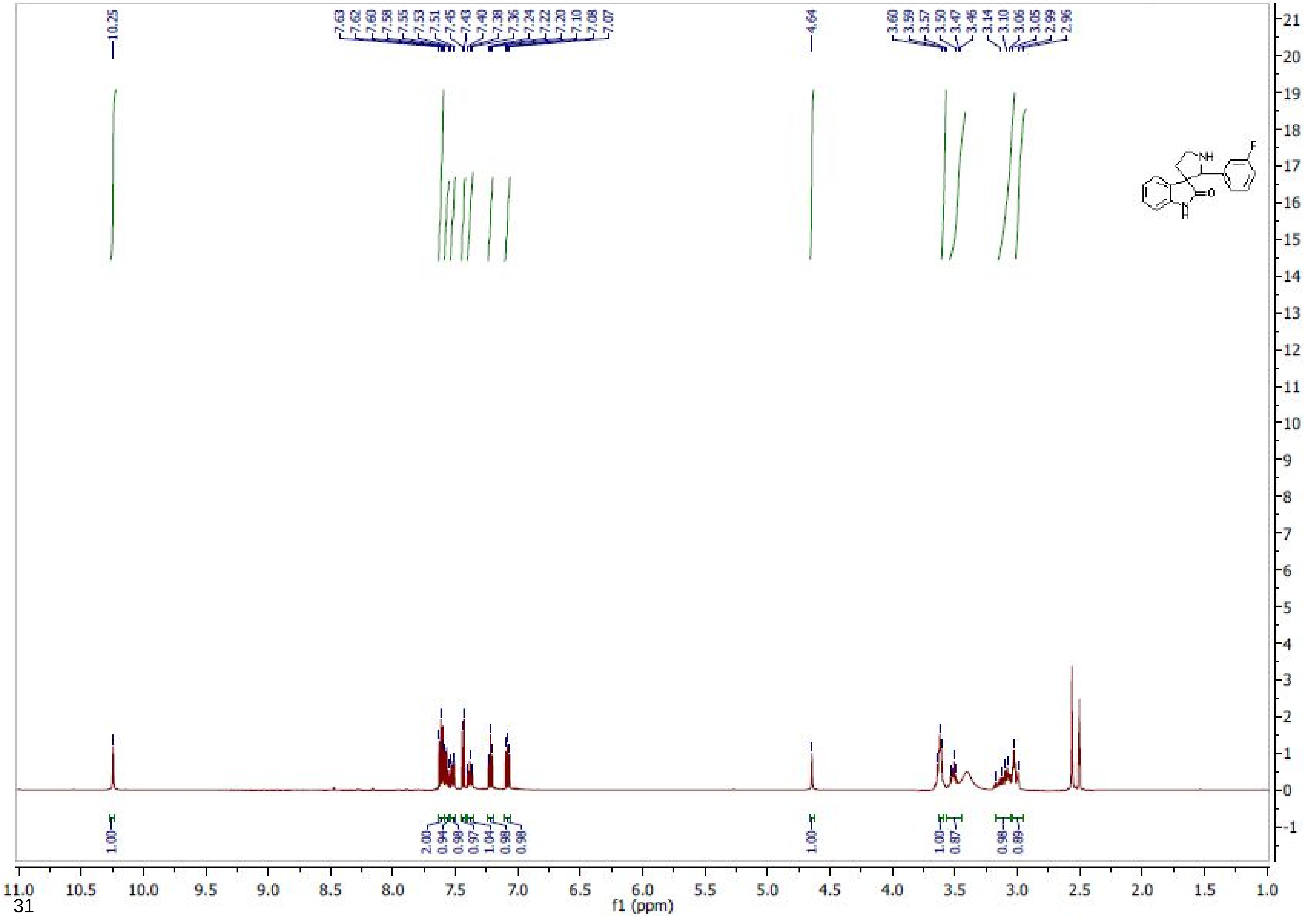
10.25
7.63 7.62 7.60 7.58 7.55 7.53 7.51 7.45 7.43 7.40 7.38 7.36 7.24 7.22 7.20 7.10 7.08 7.07
4.64
3.60 3.59 3.57 3.50 3.47 3.46 3.14 3.10 3.06 3.05 2.99 2.96
1.00
2.00 0.94 0.98 0.97 1.04 0.98 0.98
1.00
1.00 0.87
0.98 0.89
F
NH
O
H
11.0 10.5 10.0 9.5 9.0 8.5 8.0 7.5 7.0 6.5 6.0 5.5 5.0 4.5 4.0 3.5 3.0 2.5 2.0 1.5 1.0
f1 (ppm)

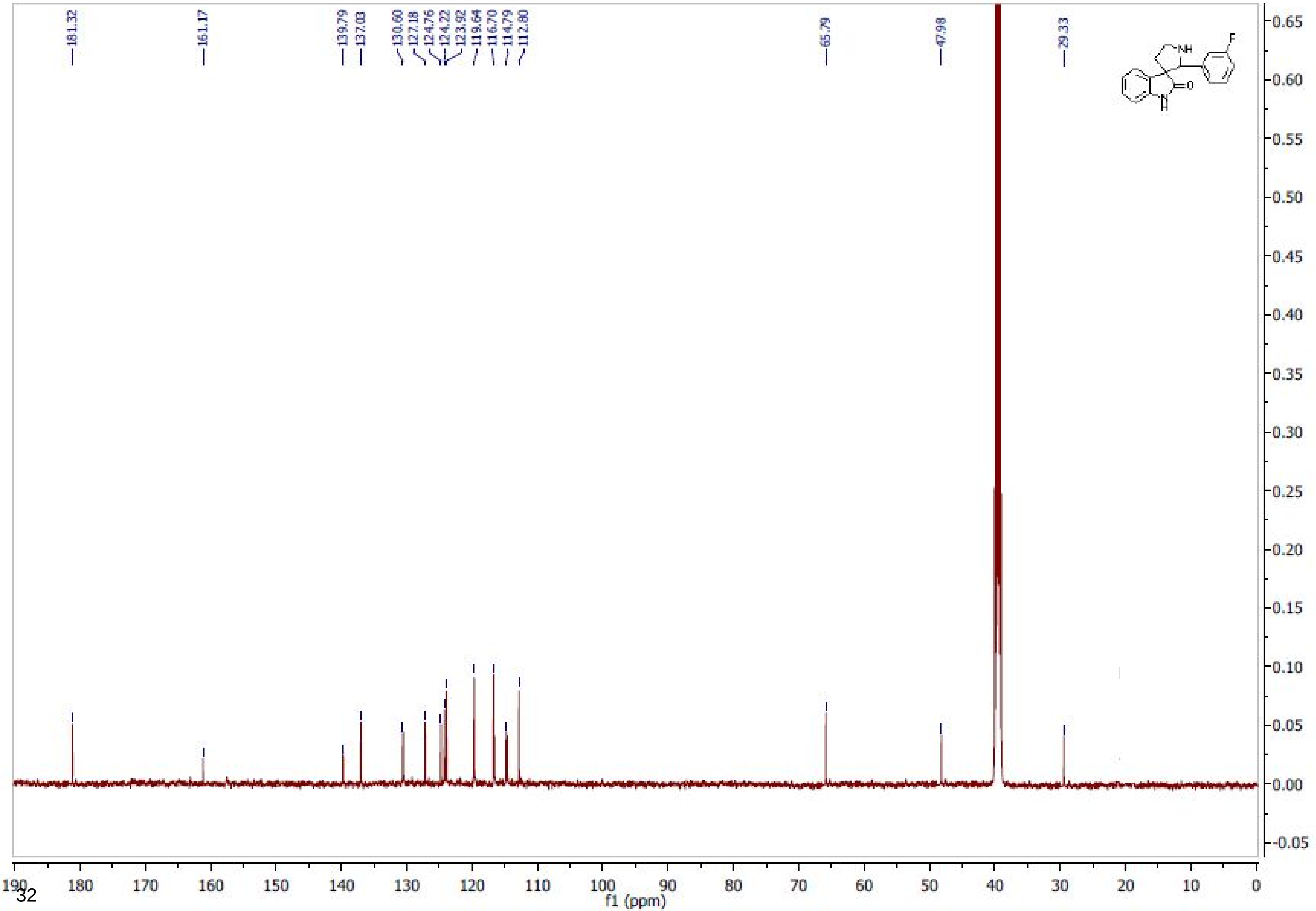
181.32
161.17
139.79
137.03
130.60
127.18
124.76
124.22
123.92
119.64
116.70
114.79
112.80
65.79
47.98
29.33
NH
F
O
H
f1 (ppm)

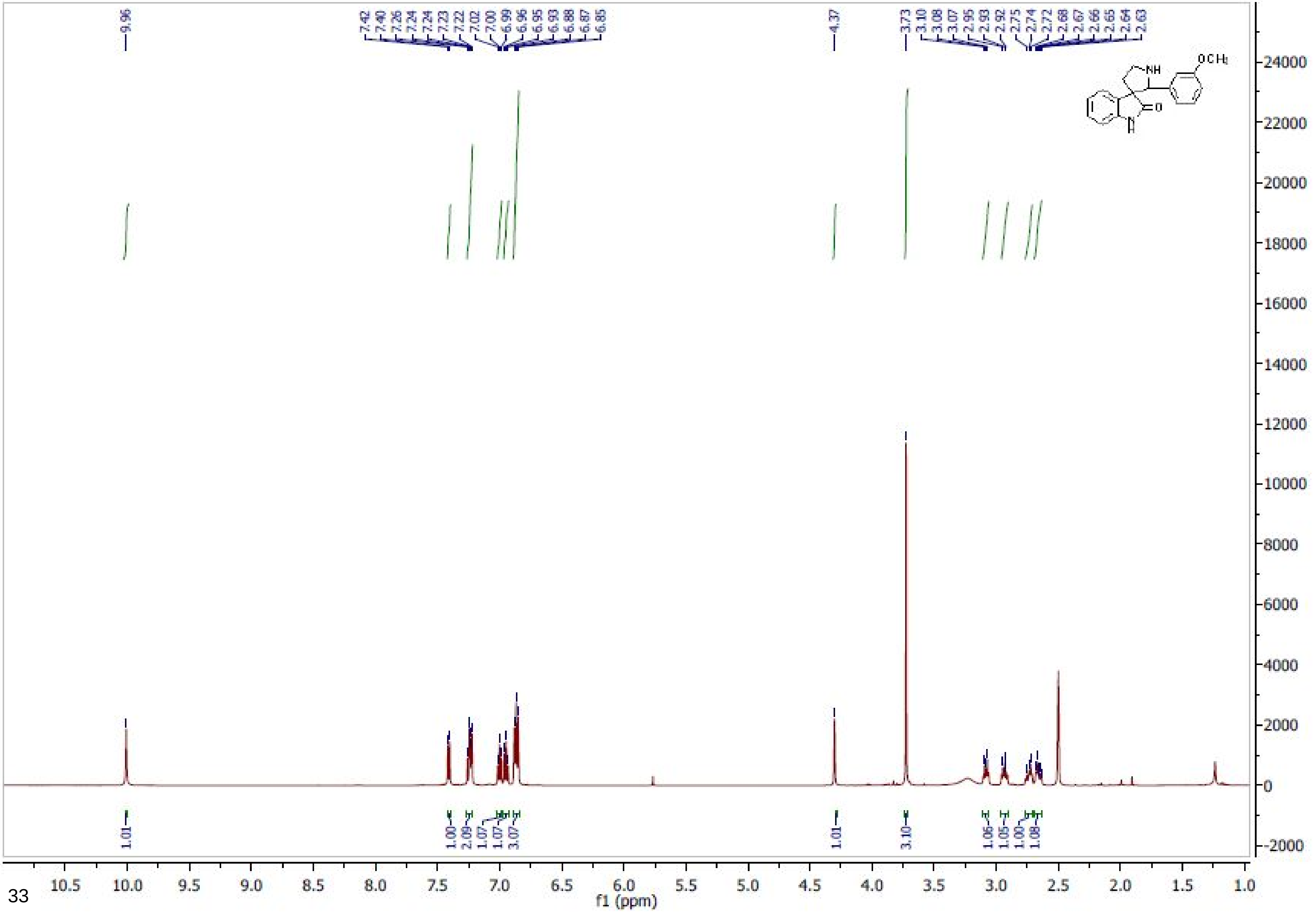
9.96
7.42
7.40
7.26
7.24
7.24
7.23
7.22
7.02
7.00
6.99
6.96
6.95
6.93
6.88
6.87
6.85
4.37
3.73
3.10
3.08
3.07
2.95
2.93
2.92
2.75
2.74
2.72
2.68
2.67
2.66
2.65
2.64
2.63
NH
OCH3
O
H
1.01
1.00
2.09
1.07
1.07
3.07
1.01
3.10
1.06
1.05
1.00
1.08
24000
22000
20000
18000
16000
14000
12000
10000
8000
6000
4000
2000
0
-2000
10.5
10.0
9.5
9.0
8.5
8.0
7.5
7.0
6.5
6.0
5.5
5.0
4.5
4.0
3.5
3.0
2.5
2.0
1.5
1.0
f1 (ppm)

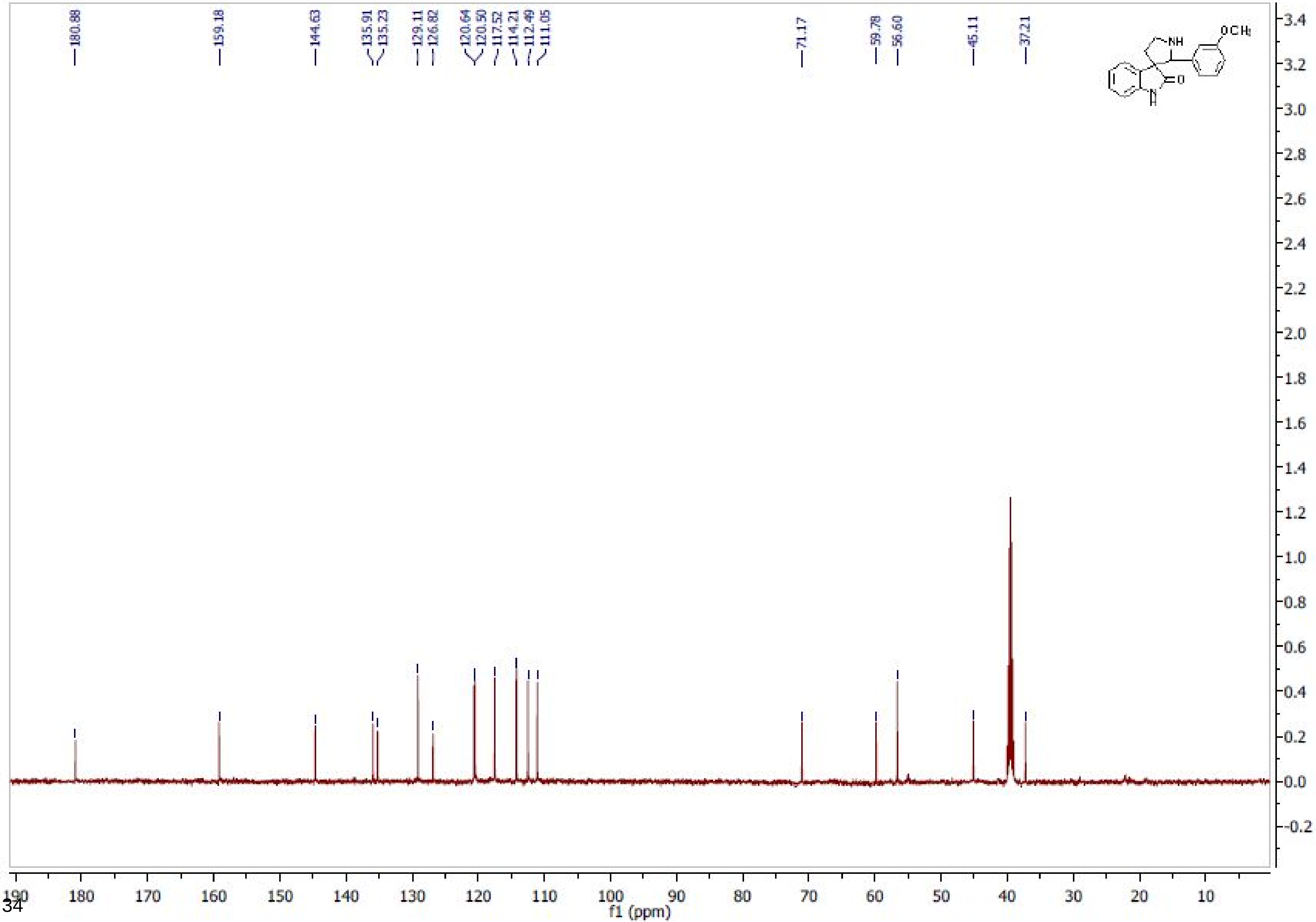
180.88
159.18
144.63
135.91
135.23
129.11
126.82
120.64
120.50
117.52
114.21
112.49
111.05
71.17
59.78
56.60
45.11
37.21
NH
OCH3
O
f1 (ppm)

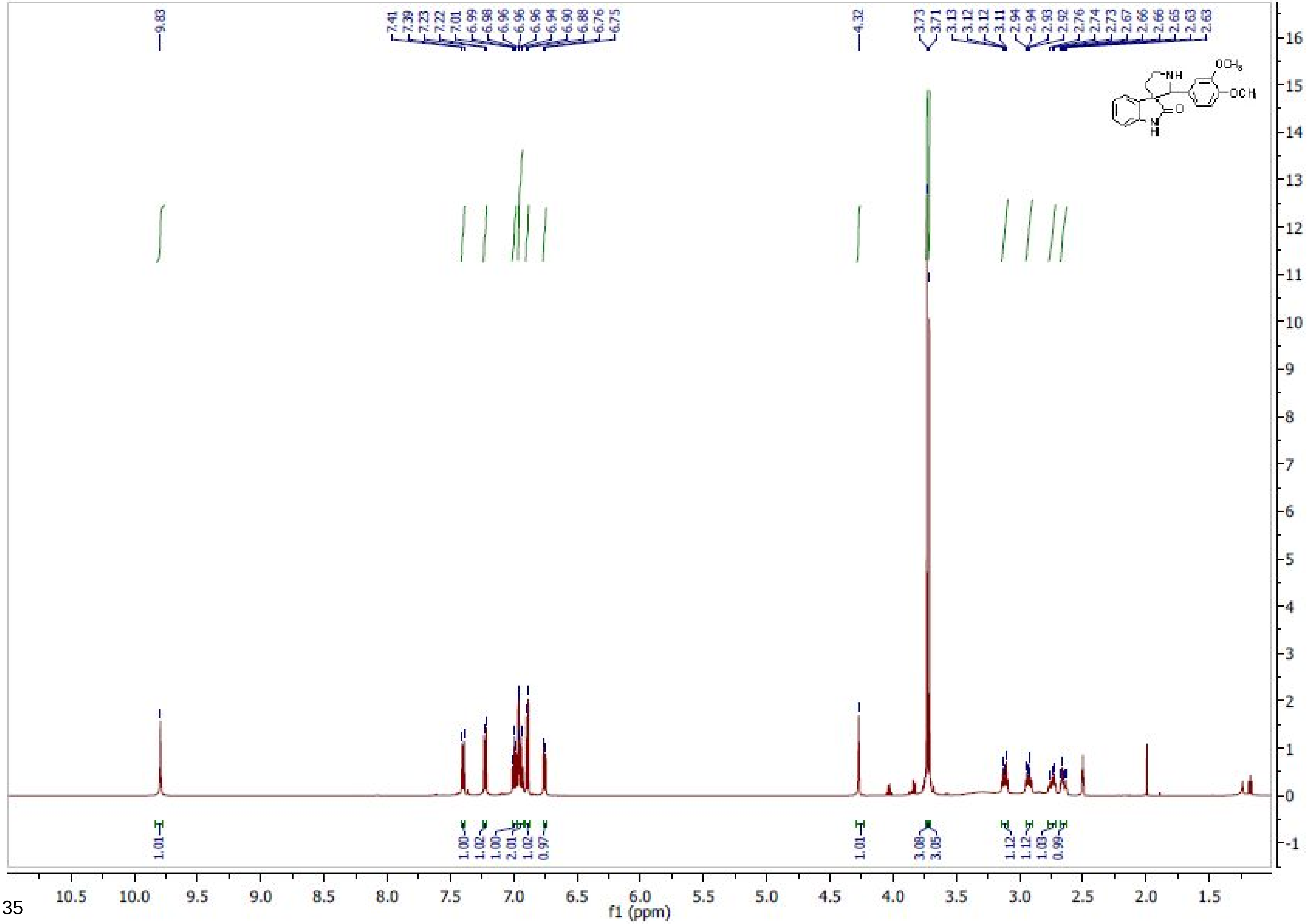
9.83
7.41
7.39
7.23
7.22
7.01
6.99
6.98
6.96
6.96
6.96
6.94
6.90
6.88
6.76
6.75
4.32
3.73
3.71
3.13
3.12
3.12
3.11
2.94
2.94
2.93
2.92
2.76
2.74
2.73
2.67
2.66
2.66
2.65
2.63
2.63
NH
OCH3
OCH3
O
N
H
1.01
1.00
1.02
1.00
2.01
1.02
0.97
1.01
3.08
3.05
1.12
1.12
1.03
0.99
f1 (ppm)

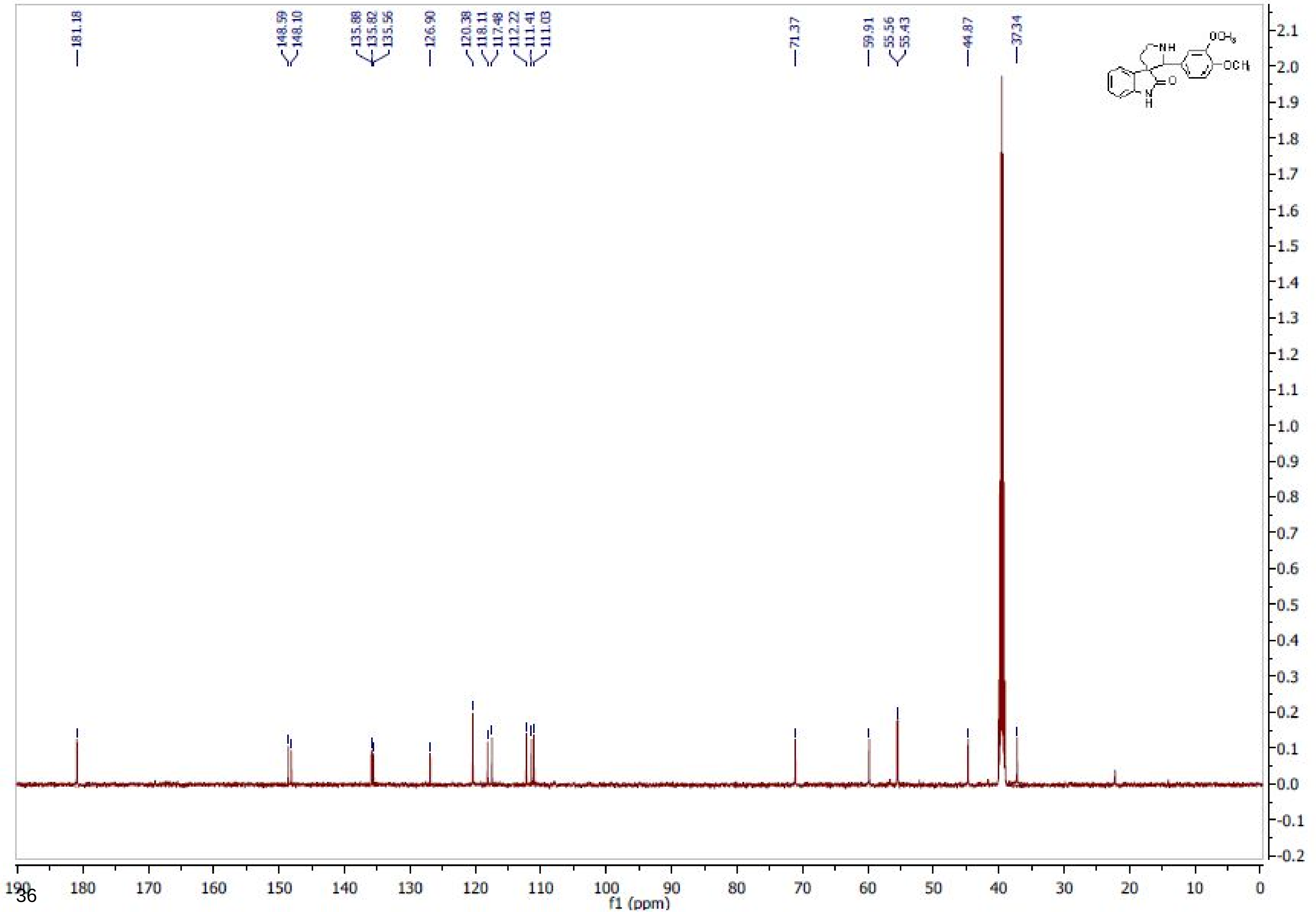
181.18
148.59
148.10
135.88
135.82
135.56
126.90
120.38
118.11
117.48
112.22
111.41
111.03
71.37
59.91
55.56
55.43
44.87
37.34
NH
OCH3
OCH3
O
N
H
f1 (ppm)

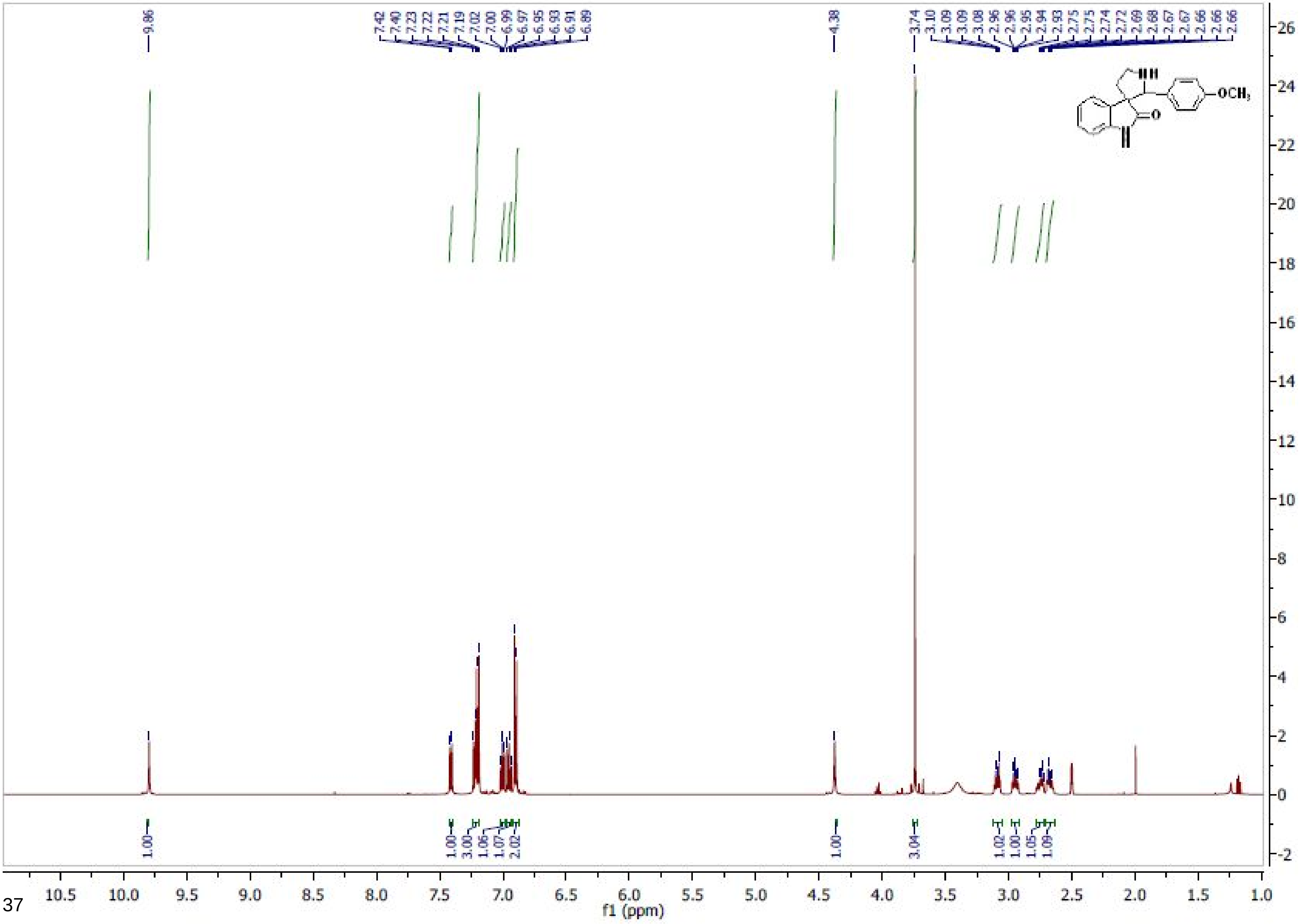

9.86
7.42
7.40
7.23
7.22
7.21
7.19
7.02
7.00
6.99
6.97
6.95
6.93
6.91
6.89
4.38
3.74
3.10
3.09
3.09
3.08
2.96
2.96
2.95
2.94
2.93
2.75
2.75
2.74
2.72
2.69
2.68
2.67
2.67
2.66
2.66
2.66
OCH3
O
1.00
1.00
3.00
1.06
1.07
2.02
1.00
3.04
1.02
1.00
1.05
1.09
f1 (ppm)

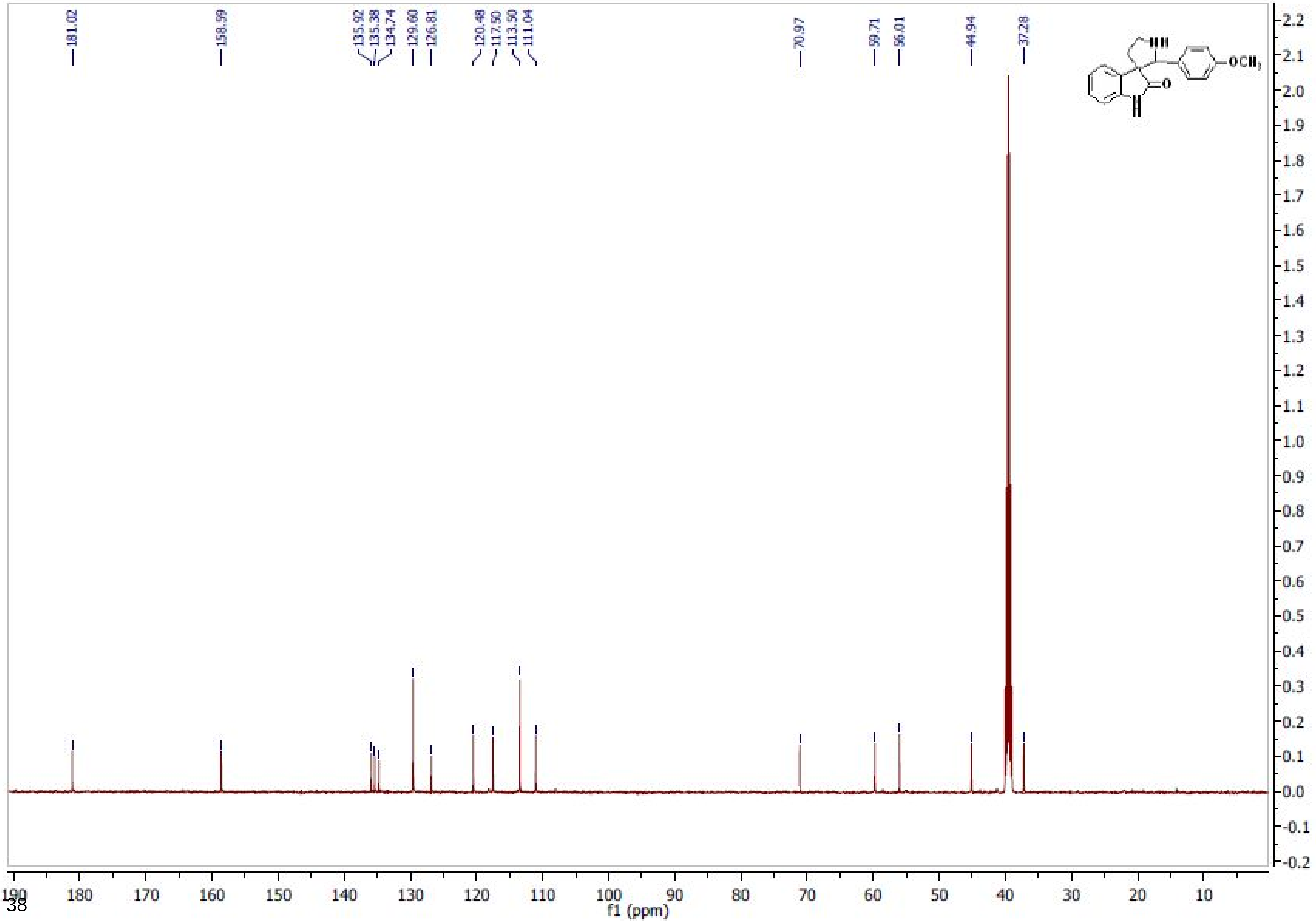
181.02
158.59
135.92
135.38
134.74
129.60
126.81
120.48
117.50
113.50
111.04
70.97
59.71
56.01
44.94
37.28
OCH3
NH
O
H
f1 (ppm)

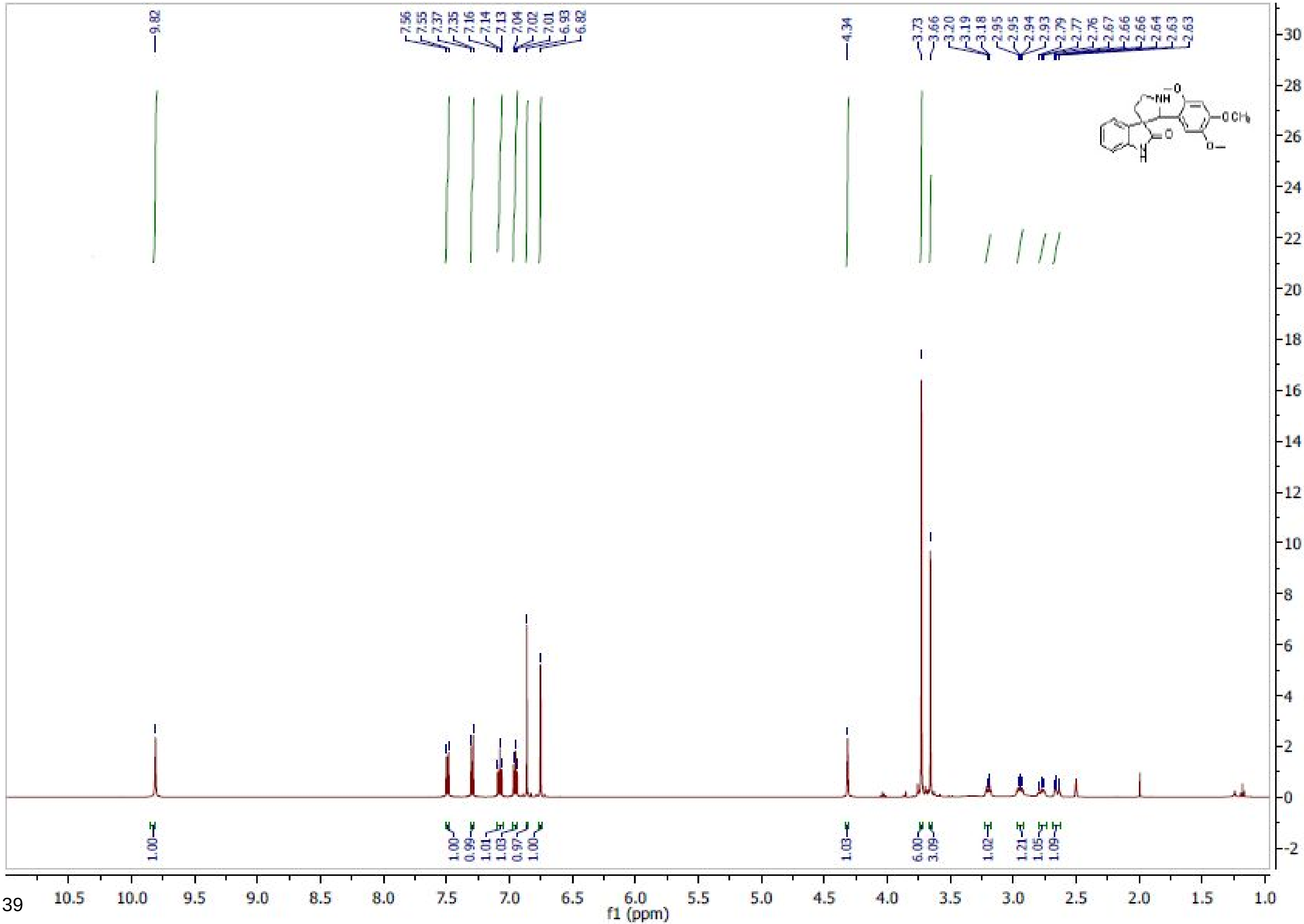

9.82
7.56
7.55
7.37
7.35
7.16
7.14
7.13
7.04
7.02
7.01
6.93
6.82
4.34
3.73
3.66
3.20
3.19
3.18
2.95
2.95
2.94
2.93
2.79
2.77
2.76
2.67
2.66
2.66
2.64
2.63
2.63
NH
O
OCH3
O
N
H
1.00
1.00
0.99
1.01
1.03
0.97
1.00
1.03
6.00
3.09
1.02
1.21
1.05
1.09
f1 (ppm)

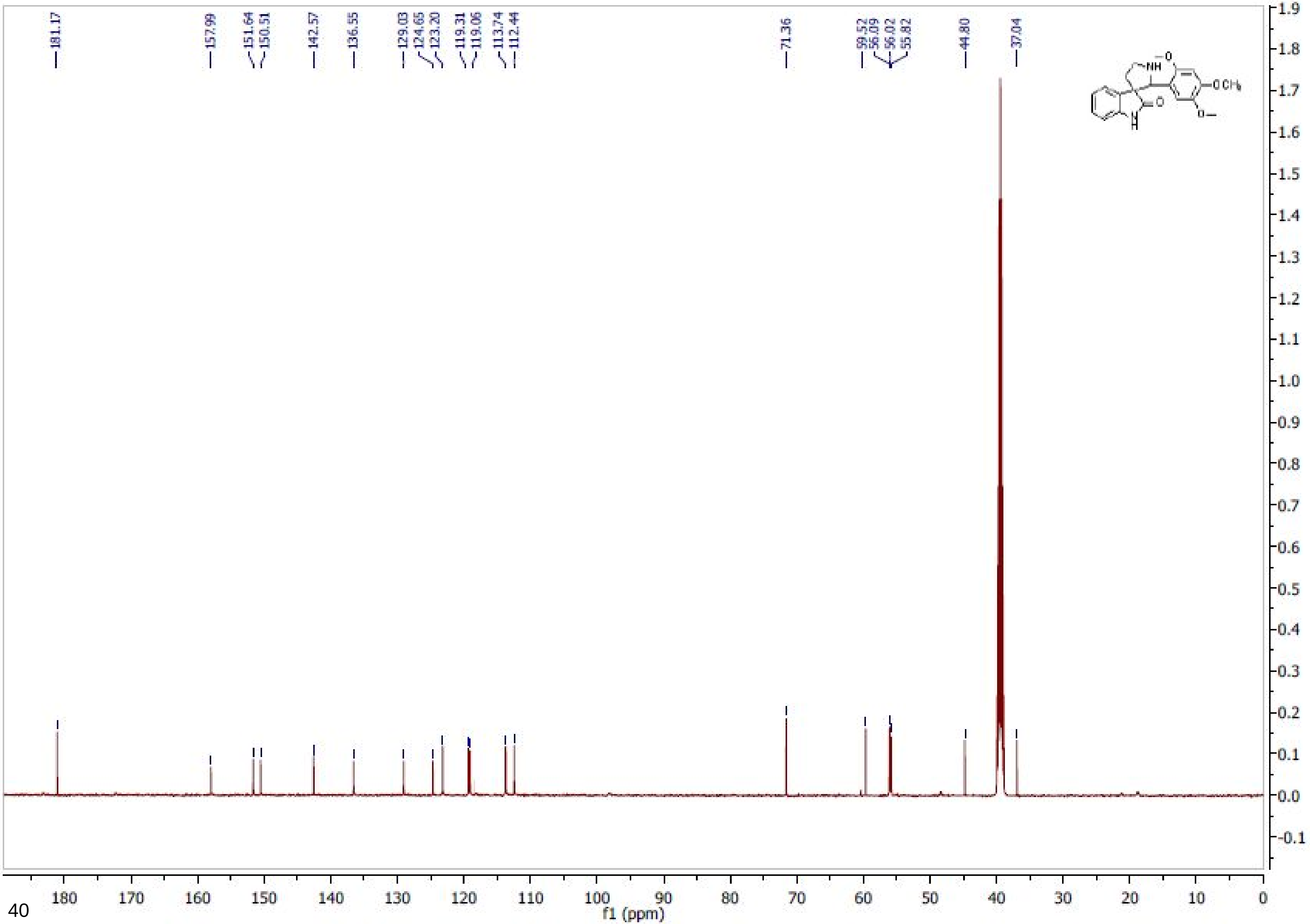

181.17
157.99
151.64
150.51
142.57
136.55
129.03
124.65
123.20
119.31
119.06
113.74
112.44
71.36
59.52
56.09
56.02
55.82
44.80
37.04
f1 (ppm)

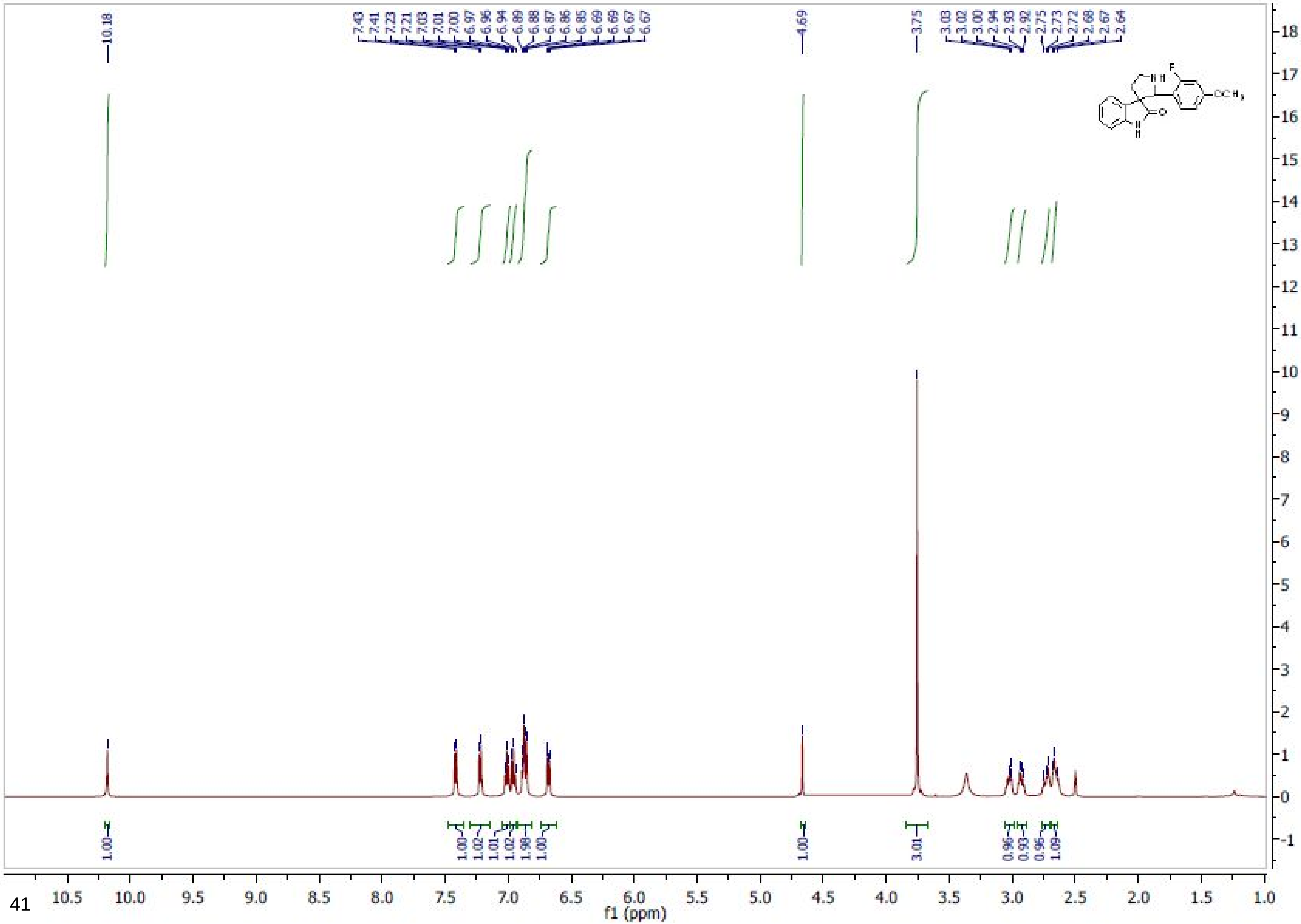

10.18
7.43
7.41
7.23
7.21
7.03
7.01
7.00
6.97
6.96
6.94
6.89
6.88
6.87
6.86
6.85
6.69
6.69
6.67
6.67
4.69
3.75
3.03
3.02
3.00
2.94
2.93
2.92
2.75
2.73
2.72
2.68
2.67
2.64
f1 (ppm)

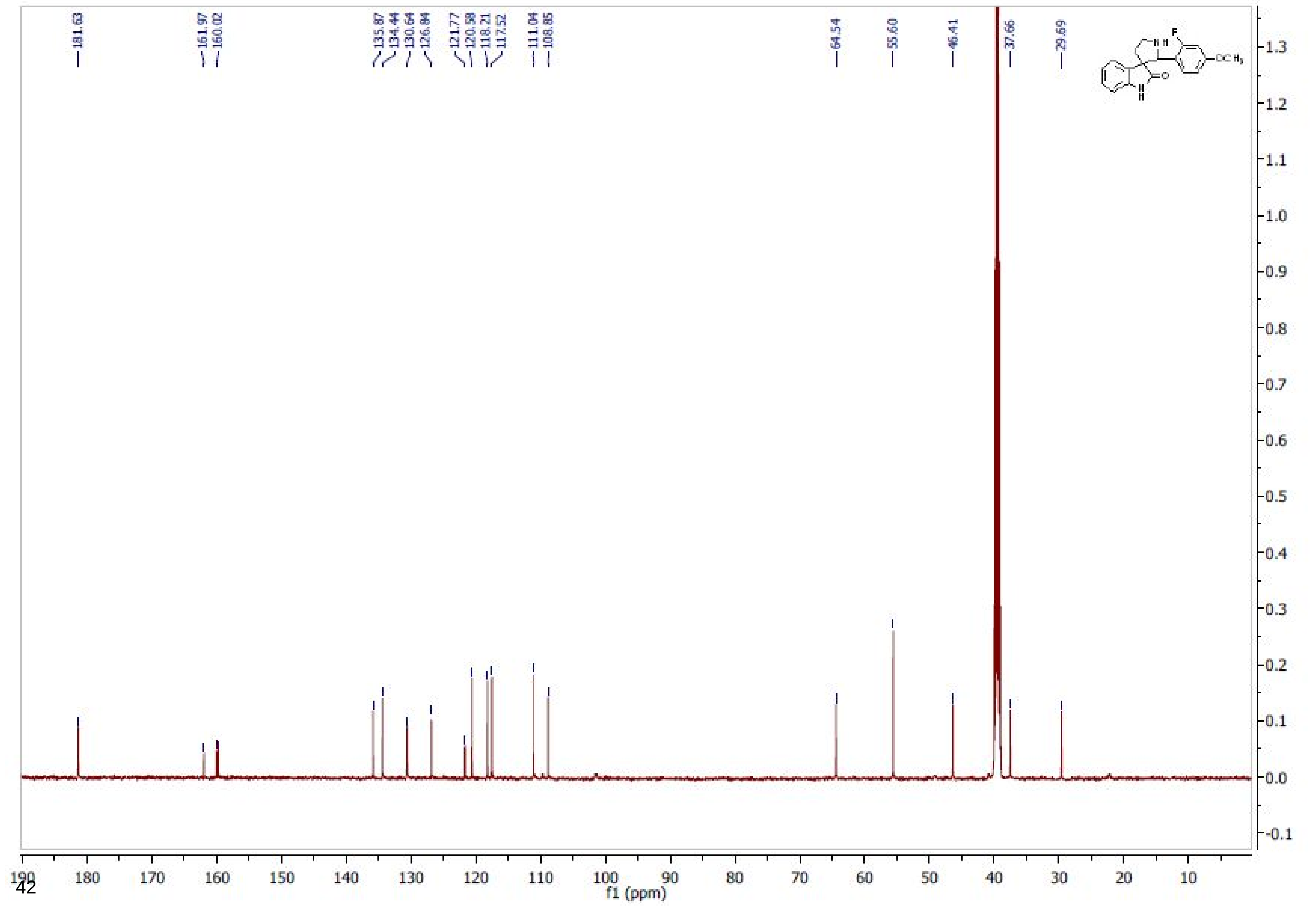

181.63
161.97
160.02
135.87
134.44
130.64
126.84
121.77
120.58
118.21
117.52
111.04
108.85
64.54
55.60
46.41
37.66
29.69
f1 (ppm)

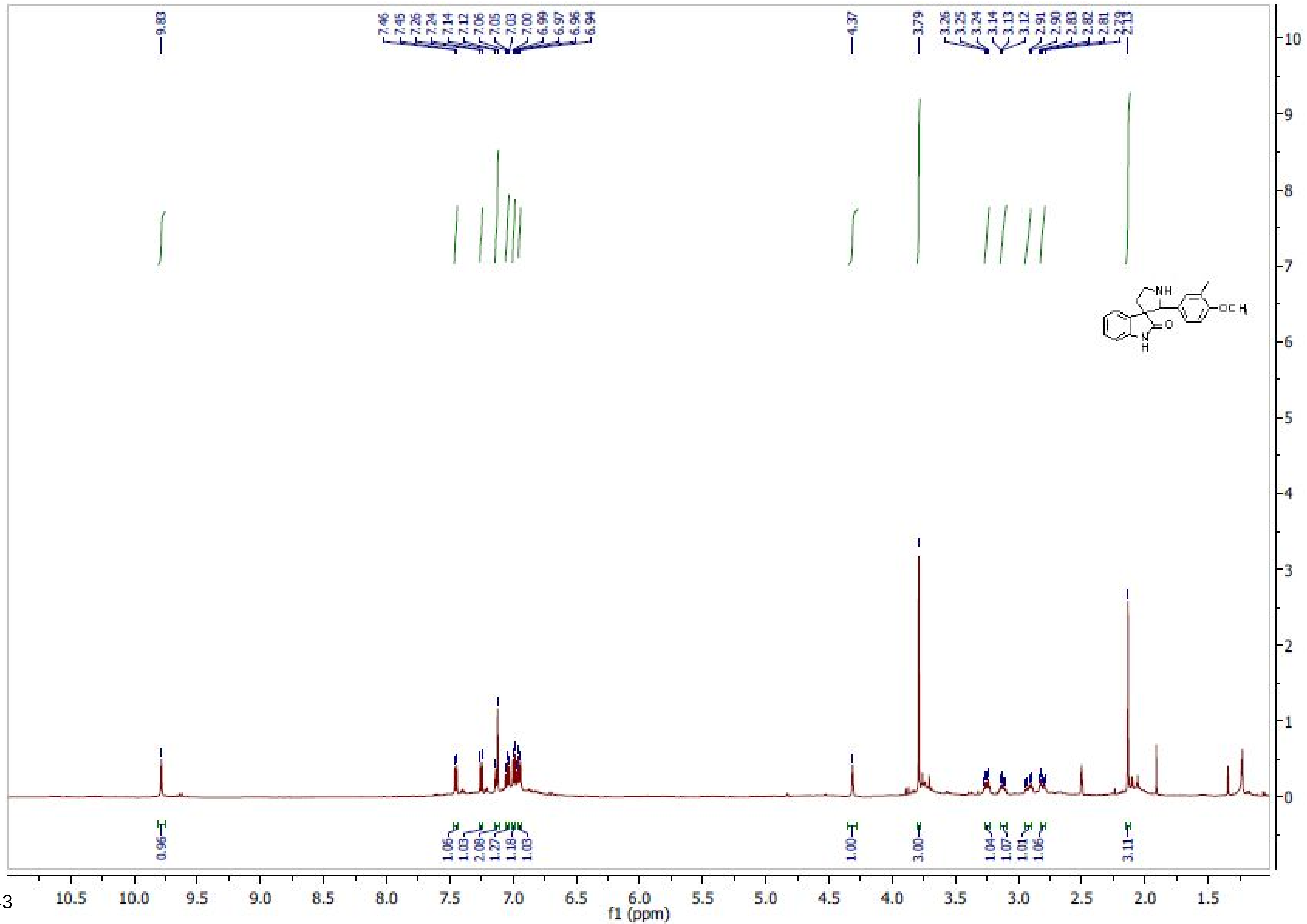
f1 (ppm)

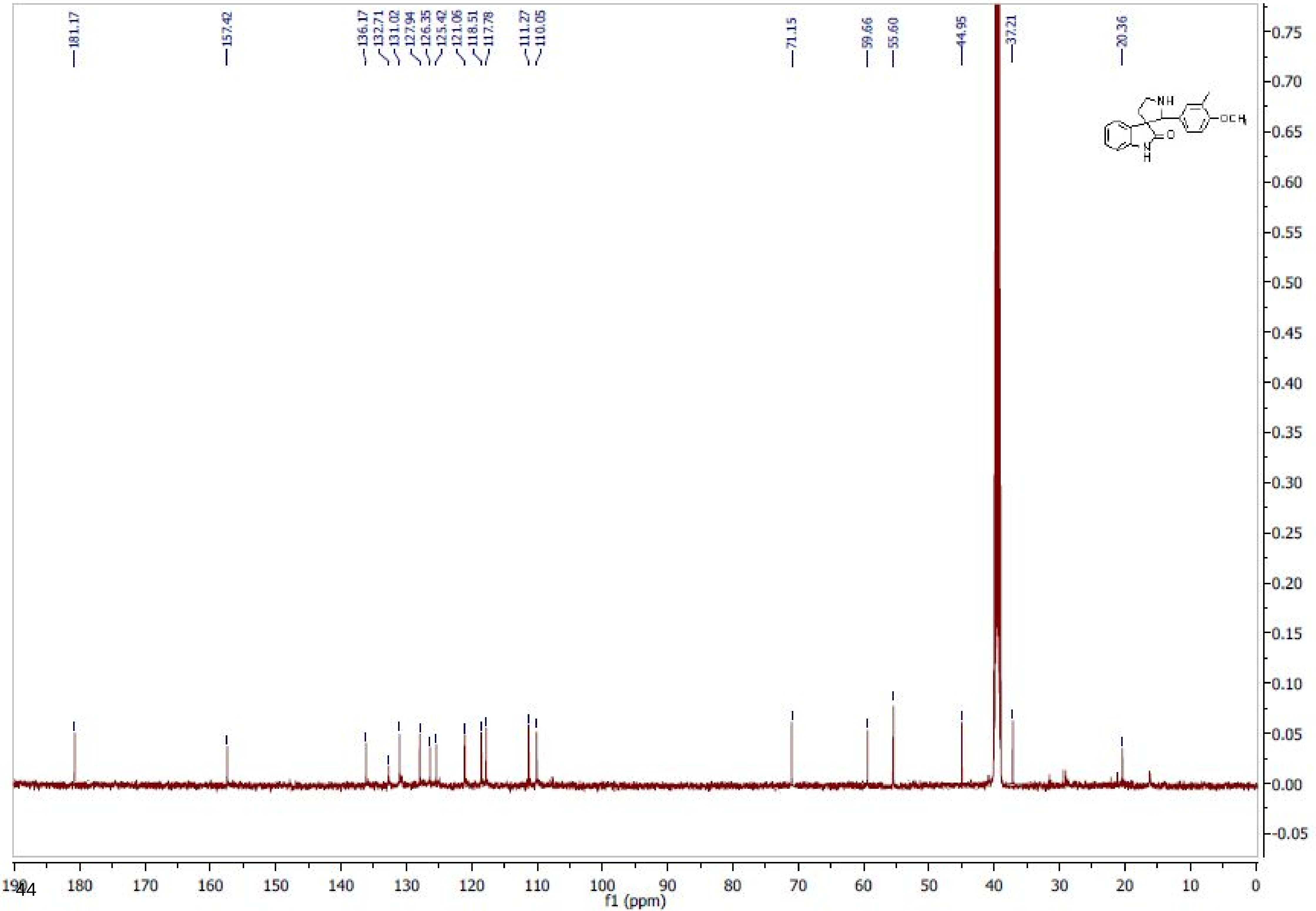
181.17
157.42
136.17
132.71
131.02
127.94
126.35
125.42
121.06
118.51
117.78
111.27
110.05
71.15
59.66
55.60
44.95
37.21
20.36
NH
OCH3
O
N H
f1 (ppm)

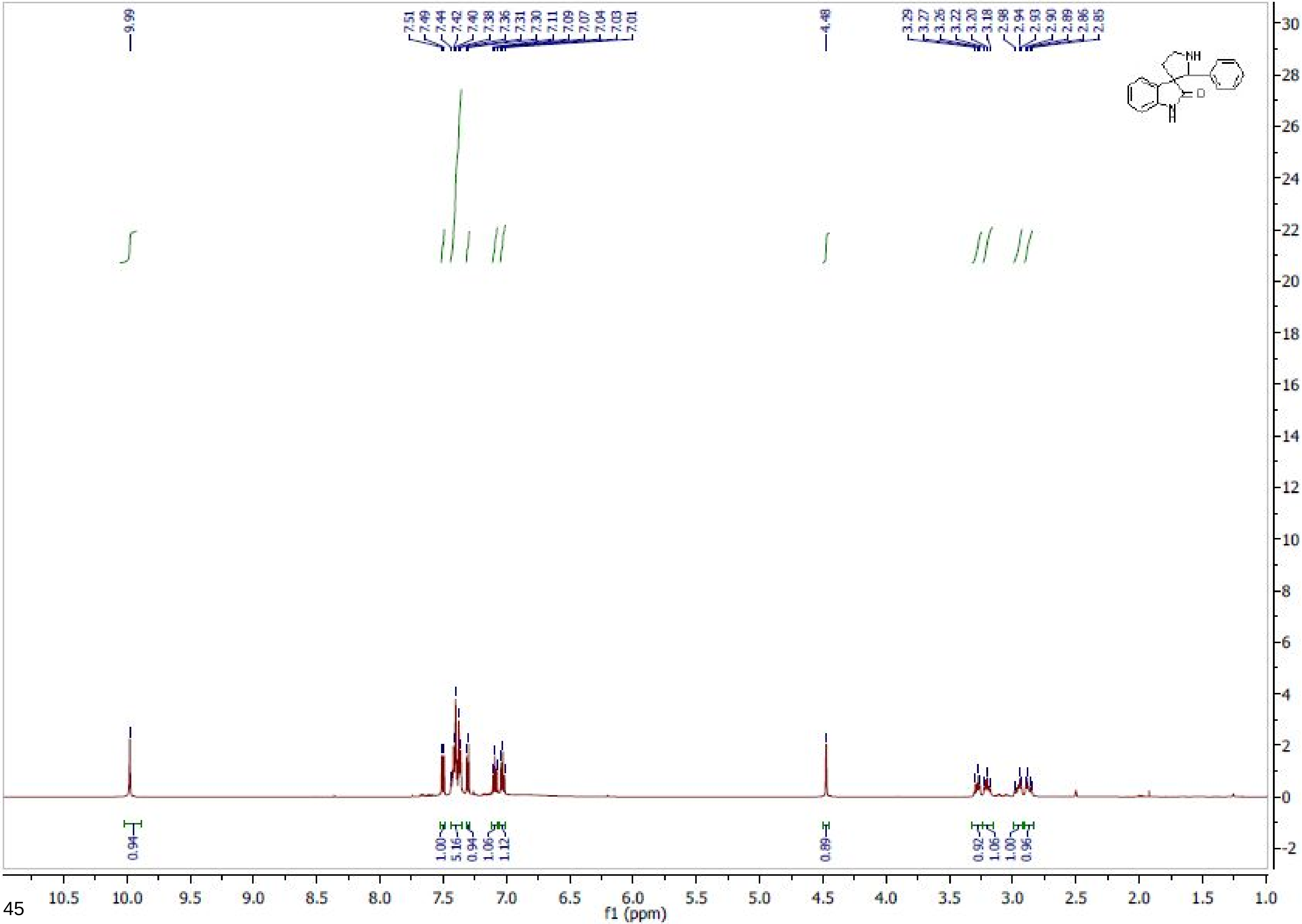
9.99
7.51 7.49 7.44 7.42 7.40 7.38 7.36 7.31 7.30 7.11 7.09 7.07 7.04 7.03 7.01
4.48
3.29 3.27 3.26 3.22 3.20 3.18 2.98 2.94 2.93 2.90 2.89 2.86 2.85
NH
O
N
H
0.94
1.00 5.16 0.94 1.06 1.12
0.89
0.92 1.06 1.00 0.96
f1 (ppm)

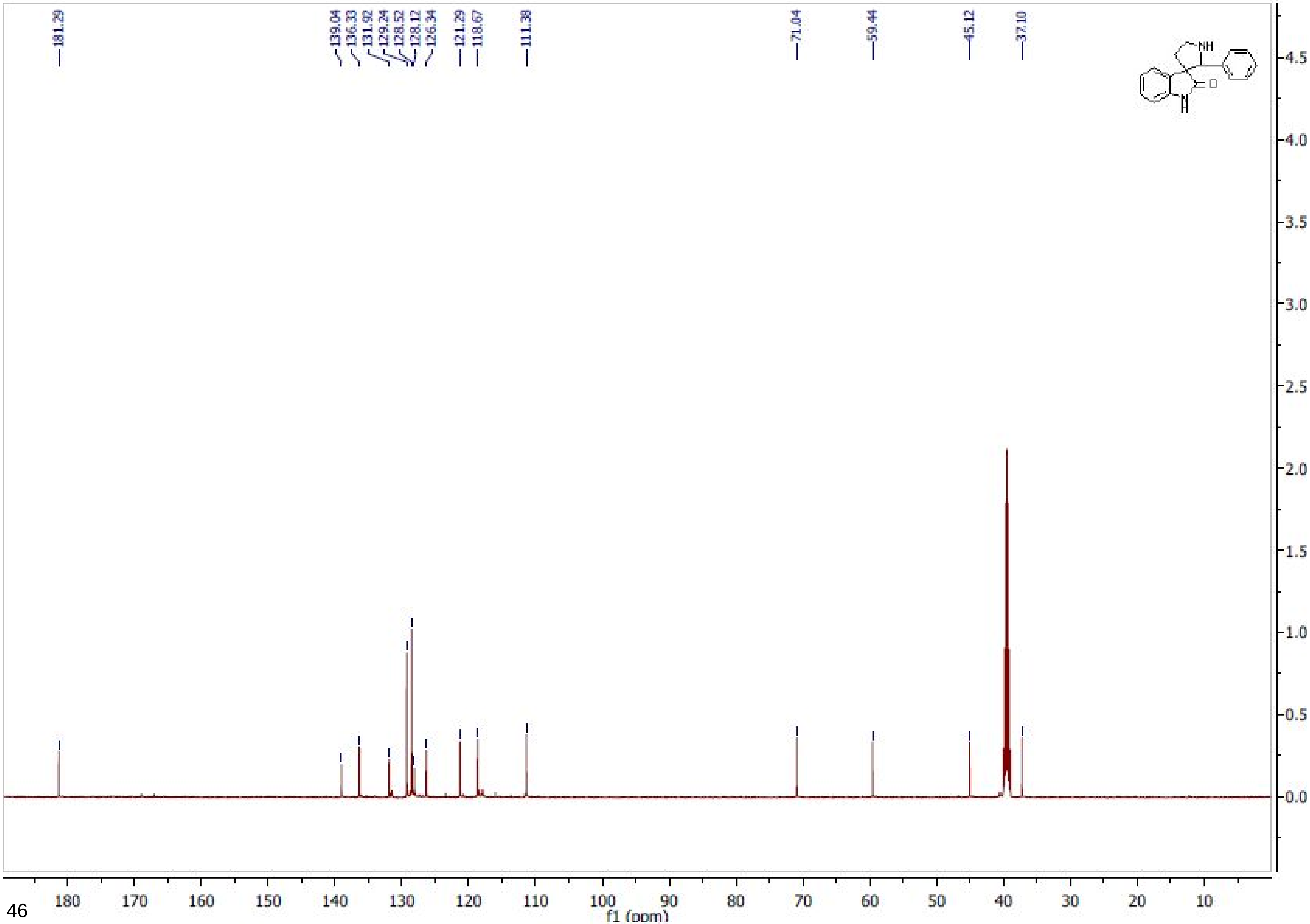
181.29
139.04
136.33
131.92
129.24
128.52
128.12
126.34
121.29
118.67
111.38
71.04
59.44
45.12
37.10
NH
O
H
f1 (ppm)

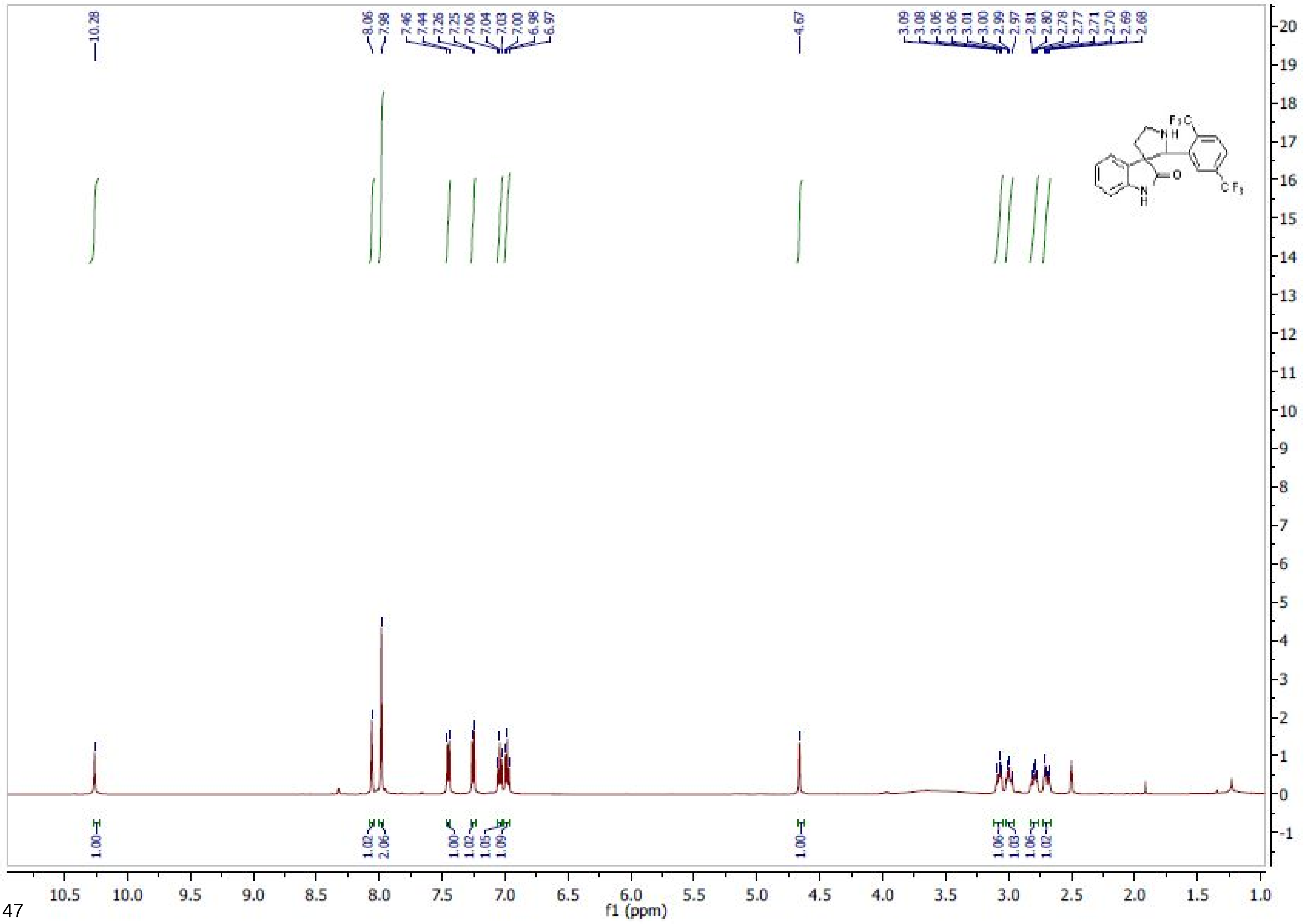
10.28
8.06
7.98
7.46
7.44
7.26
7.25
7.06
7.04
7.03
7.00
6.98
6.97
4.67
3.09
3.08
3.06
3.06
3.01
3.00
2.99
2.97
2.81
2.80
2.78
2.77
2.71
2.70
2.69
2.68
F3C
NH
O
N
H
CF3
1.00
1.02
2.06
1.00
1.02
1.05
1.09
1.00
1.06
1.03
1.06
1.02
f1 (ppm)

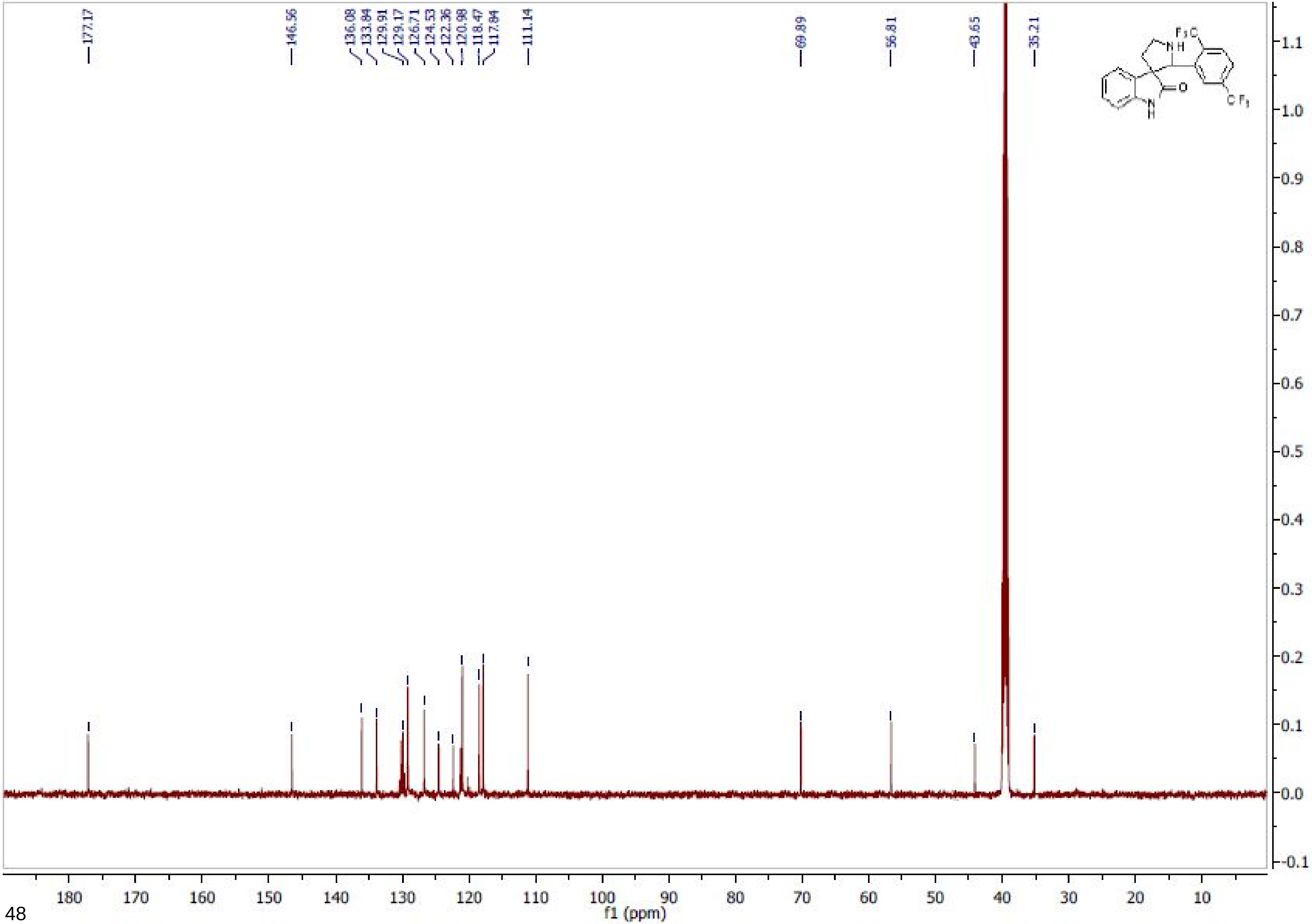
177.17
146.56
136.08
133.84
129.91
129.17
126.71
124.53
122.36
120.98
118.47
117.84
111.14
69.89
56.81
43.65
35.21
f1 (ppm)

Compound 4a

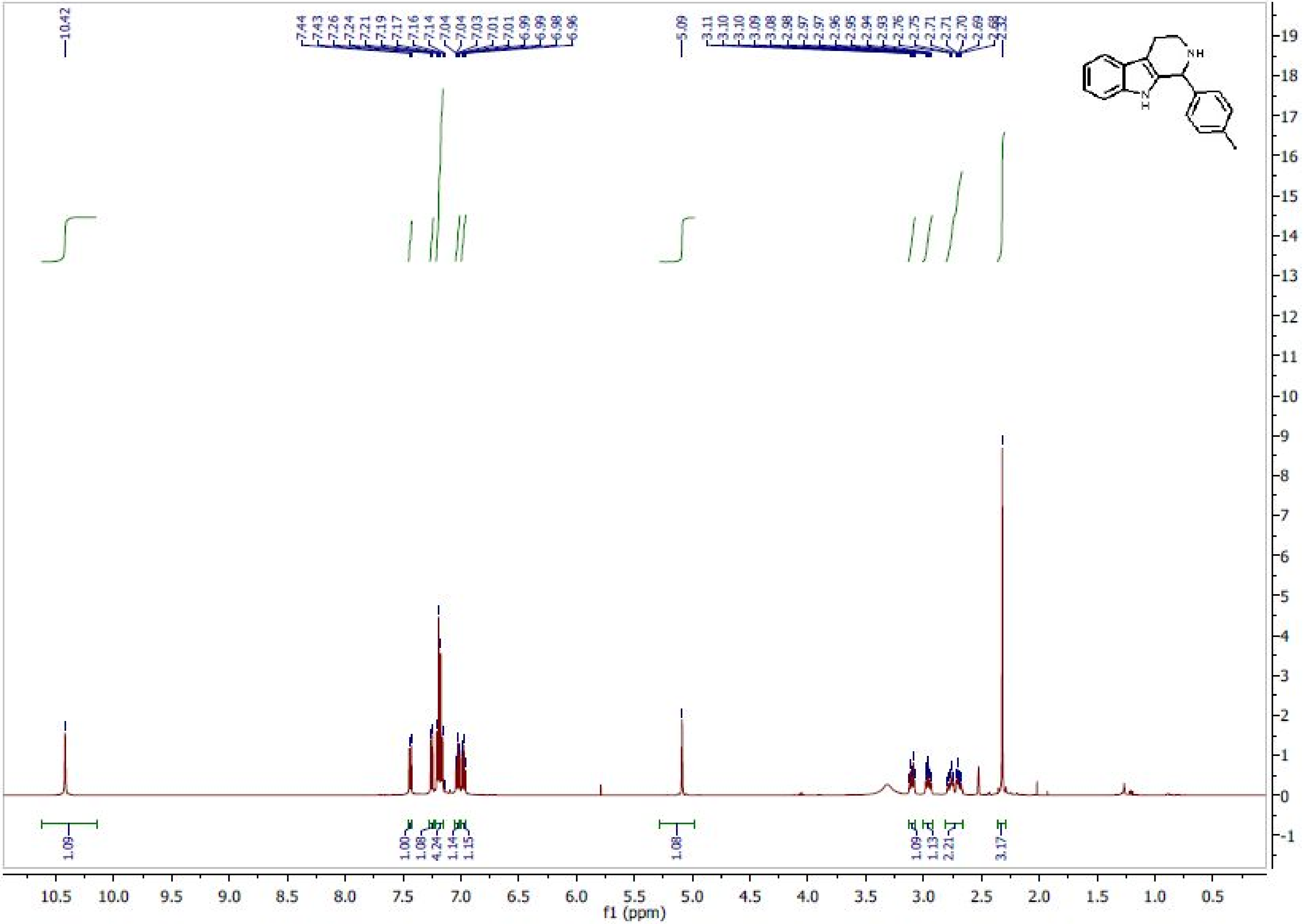

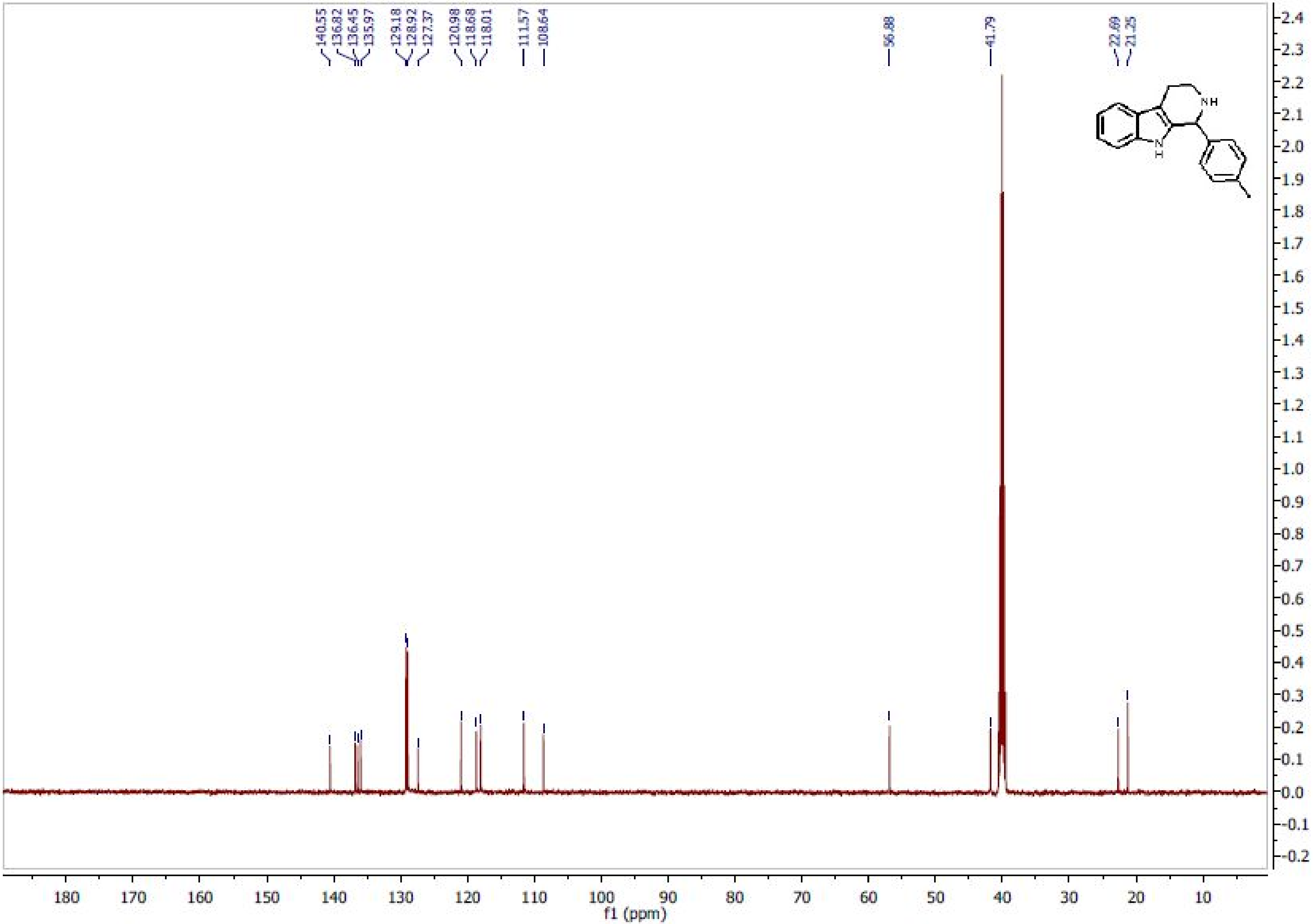
140.55
136.82
136.45
135.97
129.18
128.92
127.37
120.98
118.68
118.01
111.57
108.64
56.88
41.79
22.69
21.25
NH
H
f1 (ppm)

Compound 5a

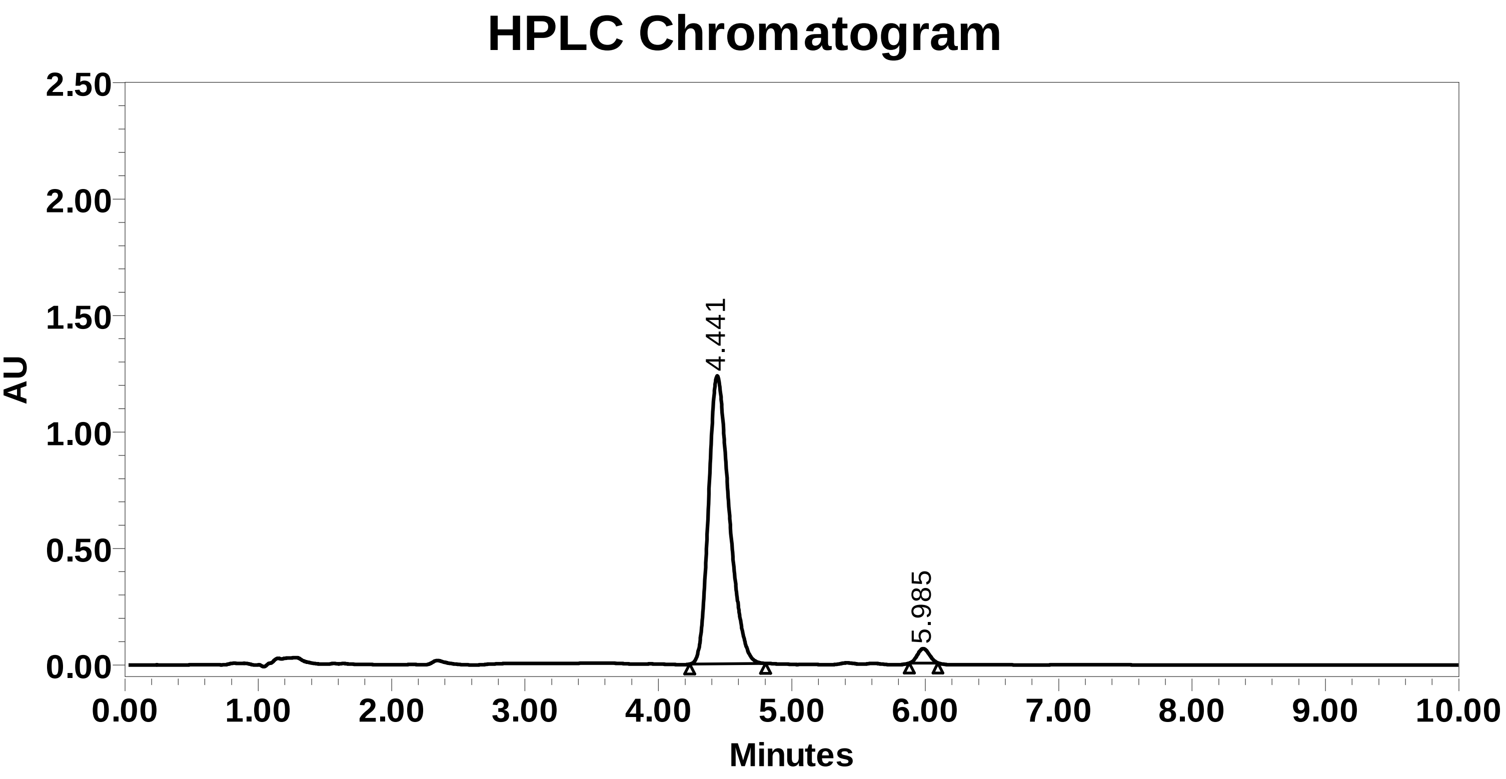

Sample Name: **6s**

Vial: 68

Inj. #: 1

Date Acquired: 3/27/2016 5:49:52 PM IST

Processed Channel: 2998 Ch1 254nm@1.2nm

	Processed Channel	Retention Time (min)	Area	% Area	Height
1	2998 Ch1 254nm@1.2nm	4.441	13336179	97.21	1236224
2	2998 Ch1 254nm@1.2nm	5.985	383189	2.79	62178

Compound 5b

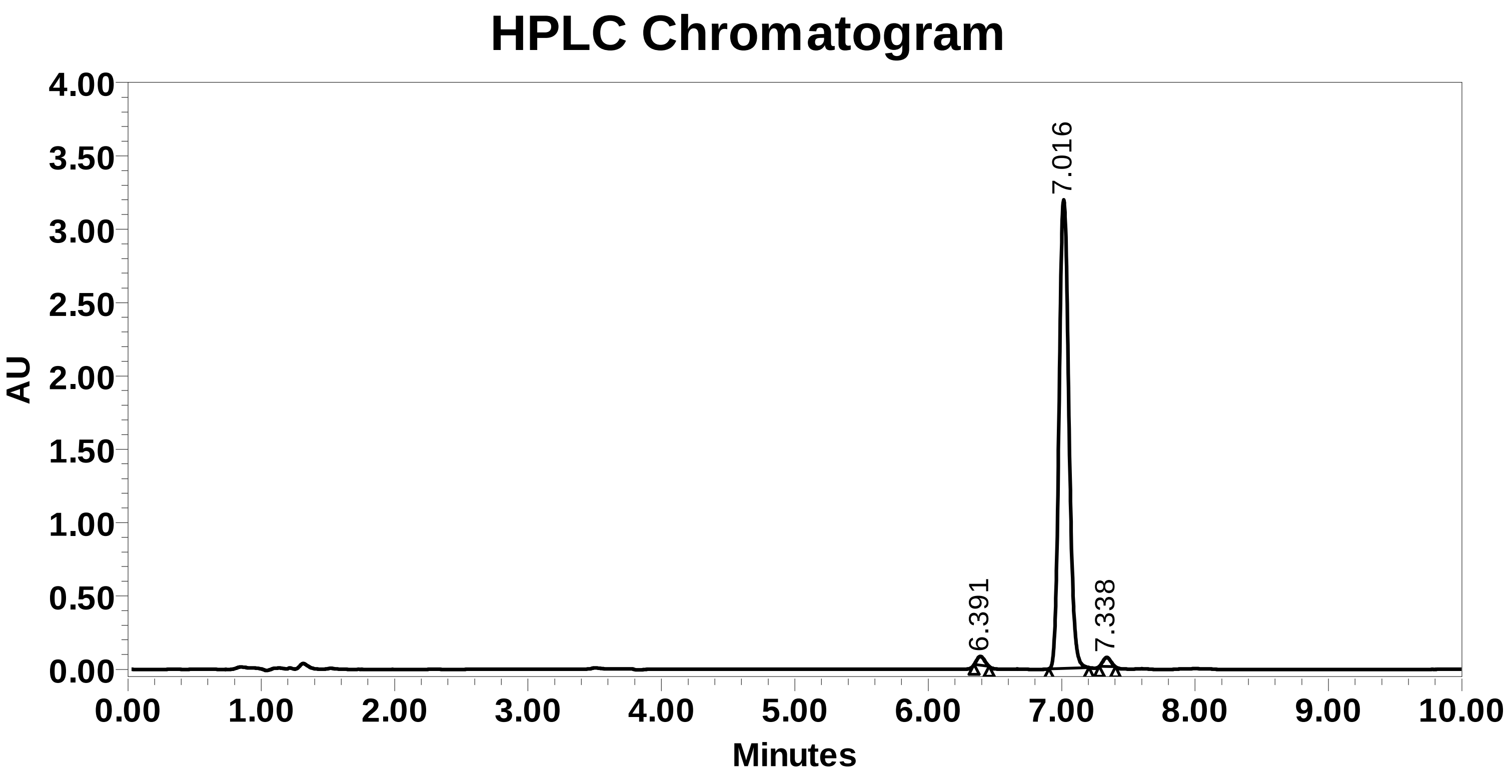

Sample Name: 4s

Vial: 66

Inj. #: 1

Date Acquired: 3/27/2016 5:26:12 PM IST

Processed Channel: 2998 Ch1 254nm@1.2nm

	Processed Channel	Retention Time (min)	Area	% Area	Height
1	2998 Ch1 254nm@1.2nm	6.391	231208	1.37	60465
2	2998 Ch1 254nm@1.2nm	7.016	16374665	97.17	3195681
3	2998 Ch1 254nm@1.2nm	7.338	246202	1.46	62713

Compound 5c

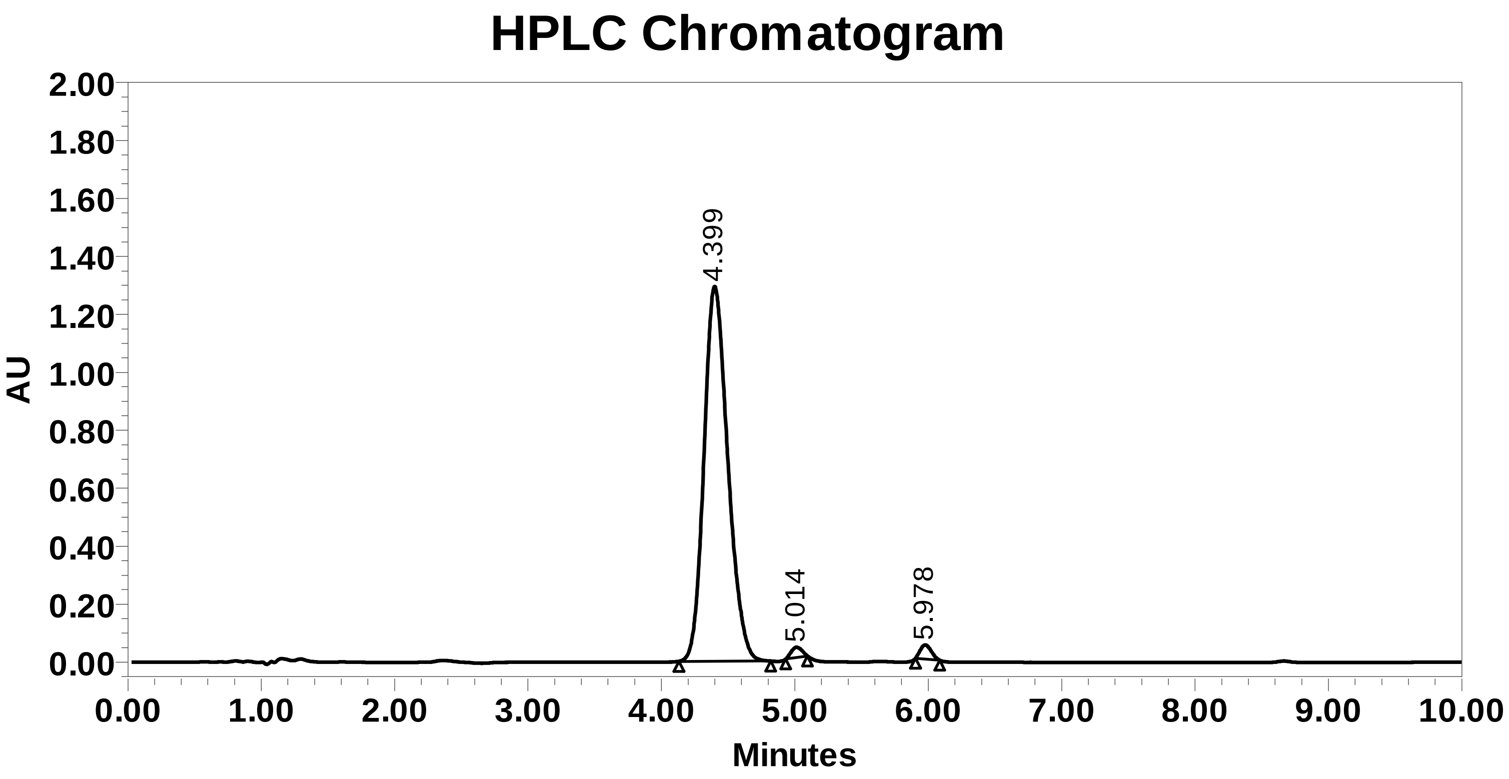

Sample Name: **5s**

Vial: 67

Inj. #: 1

Date Acquired: 3/27/2016 5:38:00 PM IST

Processed Channel: 2998 Ch1 254nm@1.2nm

	Processed Channel	Retention Time (min)	Area	% Area	Height
1	2998 Ch1 254nm@1.2nm	4.399	16089285	97.13	1293590
2	2998 Ch1 254nm@1.2nm	5.014	198077	1.20	35262
3	2998 Ch1 254nm@1.2nm	5.978	277549	1.68	48635

Compound 5d

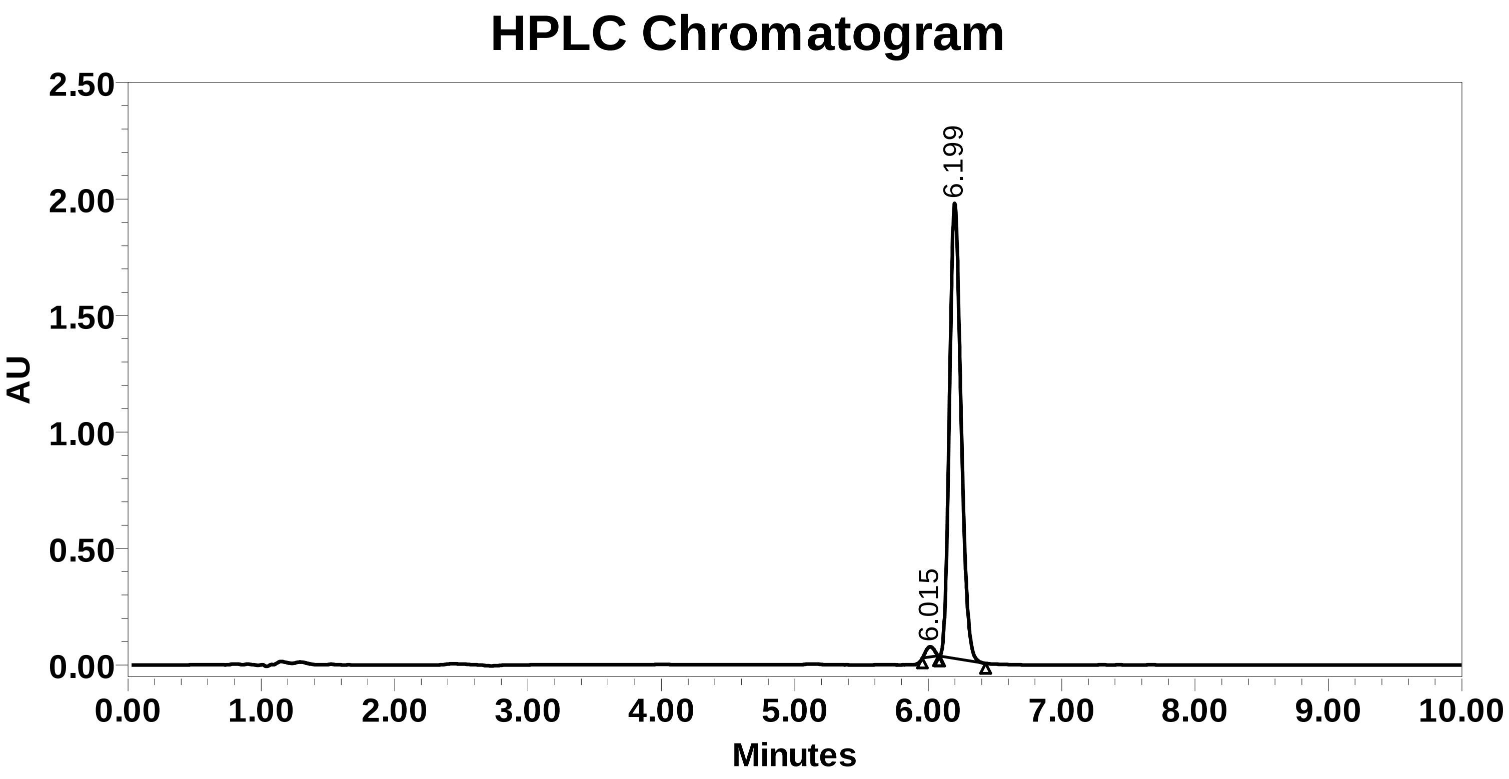

Sample Name: **8s**

Vial: 69

Inj. #: 1

Date Acquired: 3/27/2016 6:01:43 PM IST

Processed Channel: 2998 Ch1 254nm@1.2nm

	Processed Channel	Retention Time (min)	Area	% Area	Height
1	2998 Ch1 254nm@1.2nm	6.015	188891	1.58	44490
2	2998 Ch1 254nm@1.2nm	6.199	11802782	98.42	1953735

Compound 5e

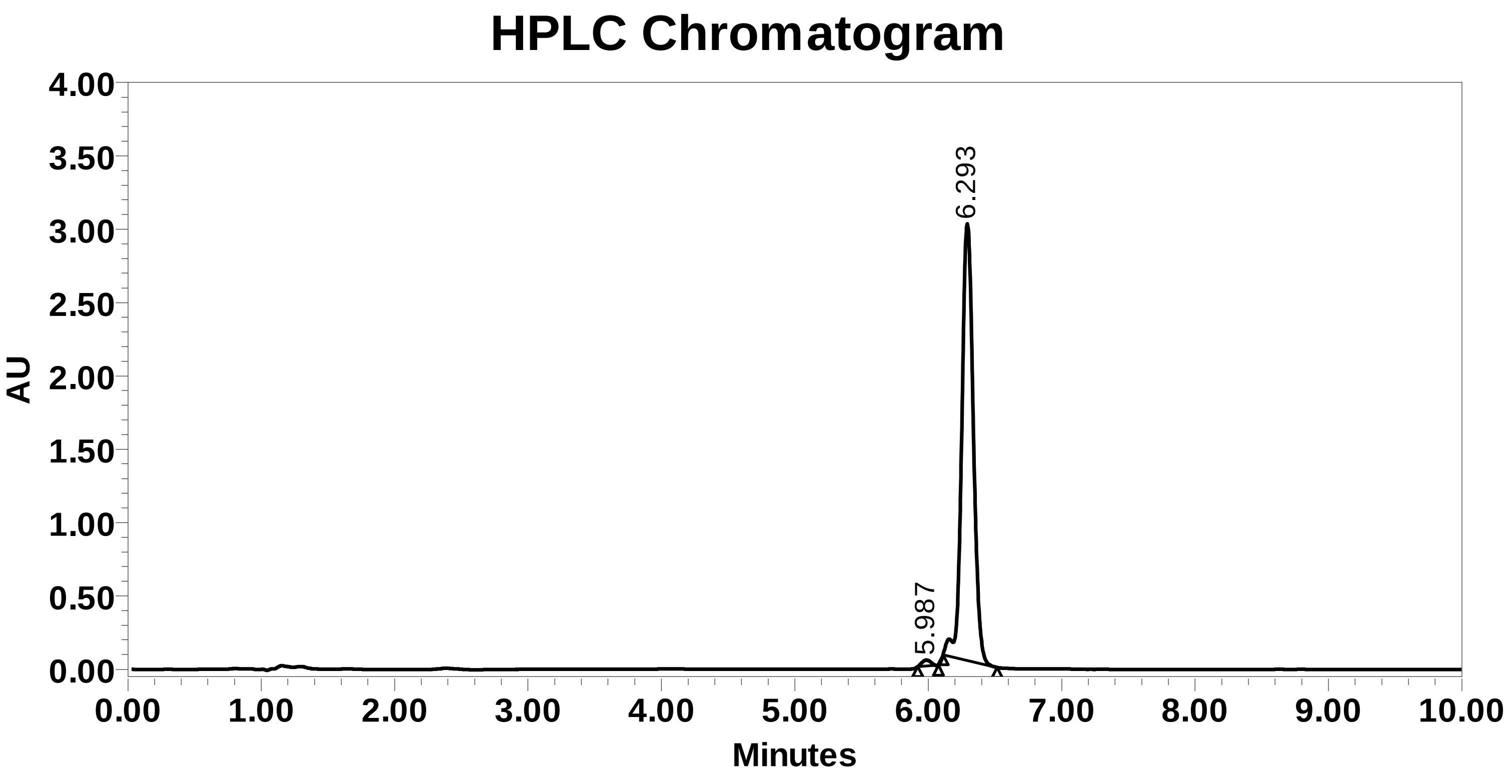

Sample Name: **9s**

Vial: 70

Inj. #: 1

Date Acquired: 3/27/2016 6:13:30 PM IST

Processed Channel: 2998 Ch1 254nm@1.2nm

	Processed Channel	Retention Time (min)	Area	% Area	Height
1	2998 Ch1 254nm@1.2nm	5.987	188546	1.03	40505
2	2998 Ch1 254nm@1.2nm	6.293	18035527	98.97	2977405

Compound 5f

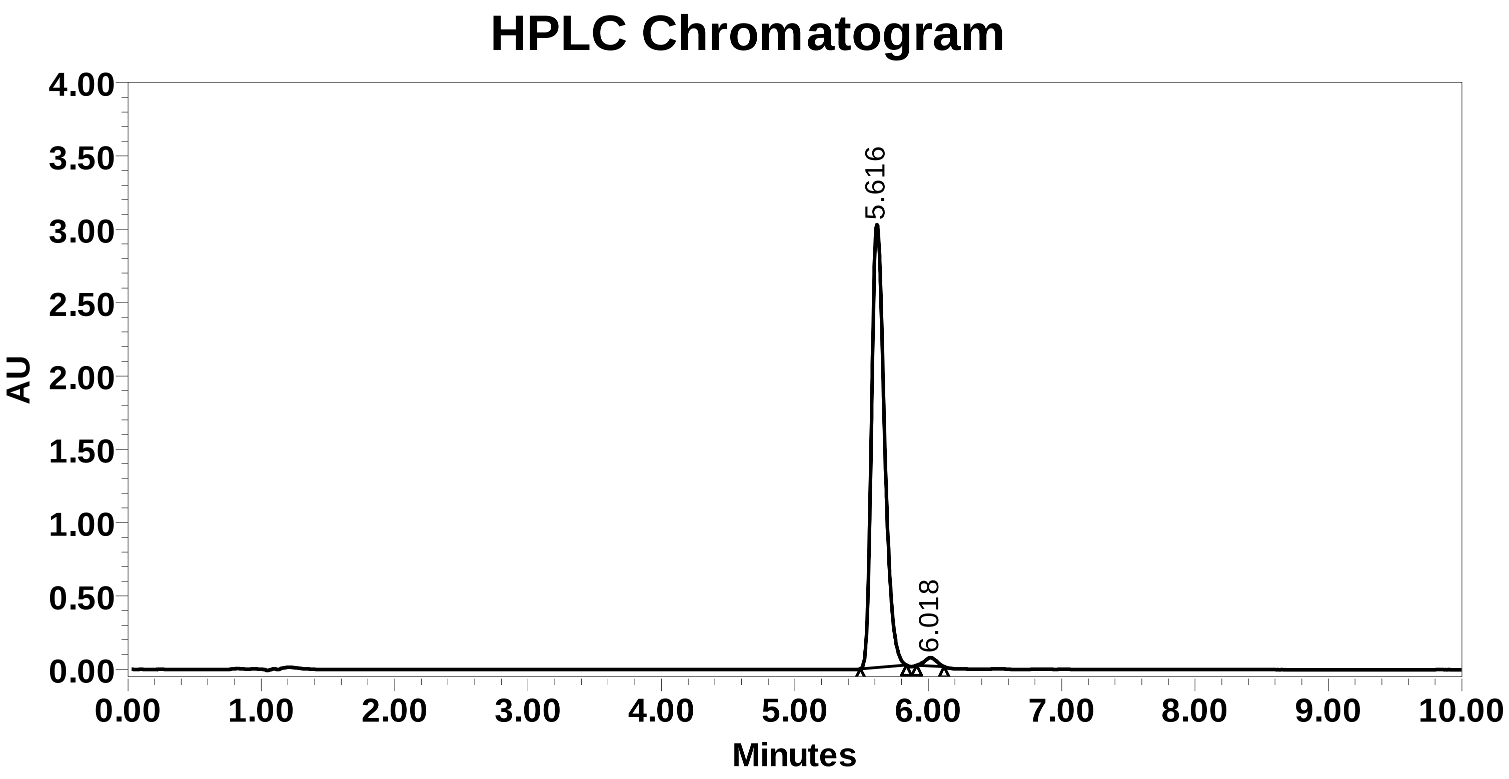

Sample Name: **11tfas**

Vial: 11

Inj. #: 1

Date Acquired: 3/28/2016 2:43:53 PM IST

Processed Channel: 2998 Ch1 254nm@1.2nm

	Processed Channel	Retention Time (min)	Area	% Area	Height
1	2998 Ch1 254nm@1.2nm	5.616	20490647	98.30	3019899
2	2998 Ch1 254nm@1.2nm	6.018	353810	1.70	56883

Compound 5g

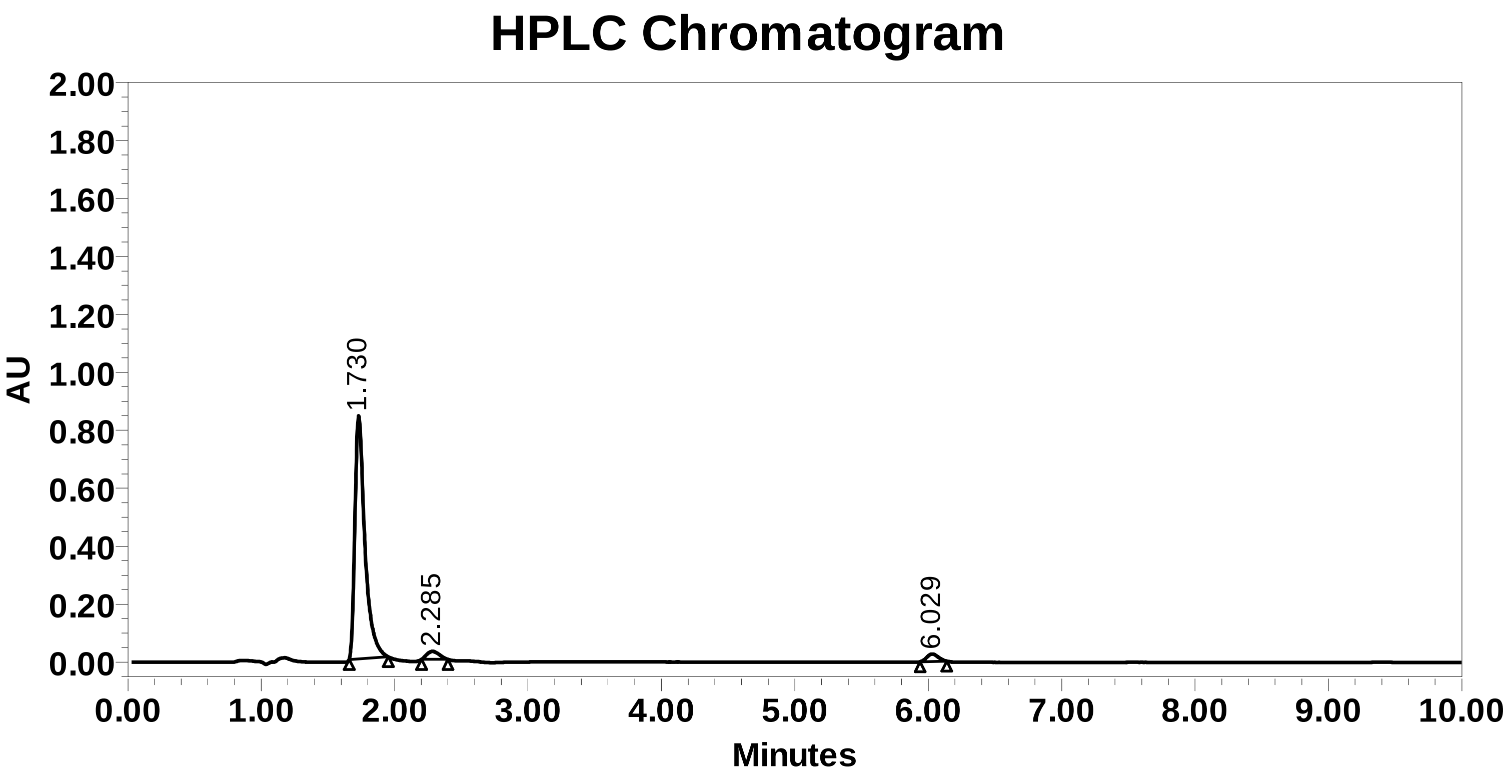

Sample Name: **11s**

Vial: 12

Inj. #: 1

Date Acquired: 3/28/2016 2:55:45 PM IST

Processed Channel: 2998 Ch1 254nm@1.2nm

	Processed Channel	Retention Time (min)	Area	% Area	Height
1	2998 Ch1 254nm@1.2nm	1.730	4395221	92.99	838467
2	2998 Ch1 254nm@1.2nm	2.285	180280	3.81	27567
3	2998 Ch1 254nm@1.2nm	6.029	15115[illegible]	3.20	26018

Compound 5h

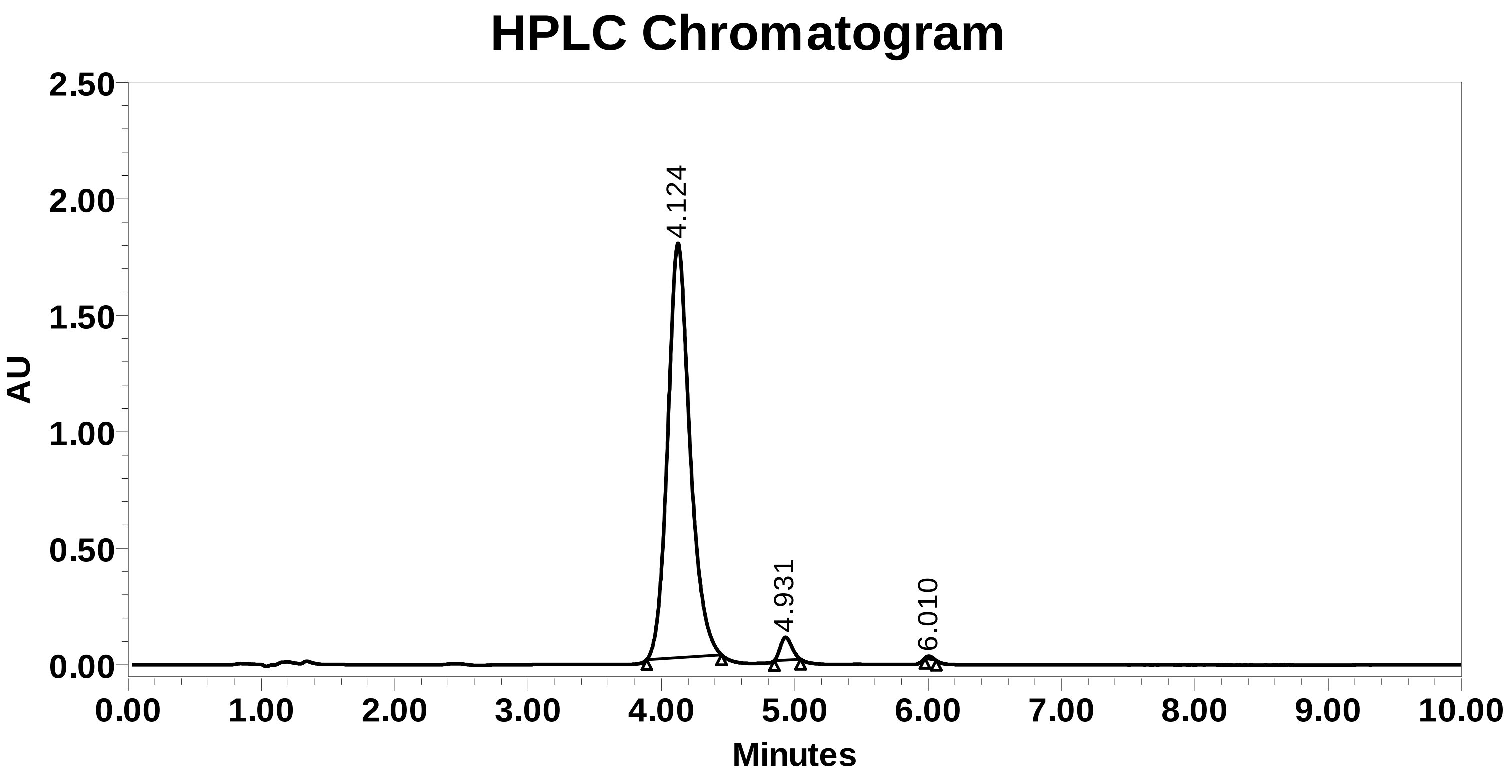

Sample Name: **12s**

Vial: 13

Inj. #: 1

Date Acquired: 3/28/2016 3:07:38 PM IST

Processed Channel: 2998 Ch1 254nm@1.2nm

	Processed Channel	Retention Time (min)	Area	% Area	Height
1	2998 Ch1 254nm@1.2nm	4.124	20675103	97.11	1778445
2	2998 Ch1 254nm@1.2nm	4.931	575948	2.71	97453
3	2998 Ch1 254nm@1.2nm	6.010	39461	0.19	12536

Compound 5i

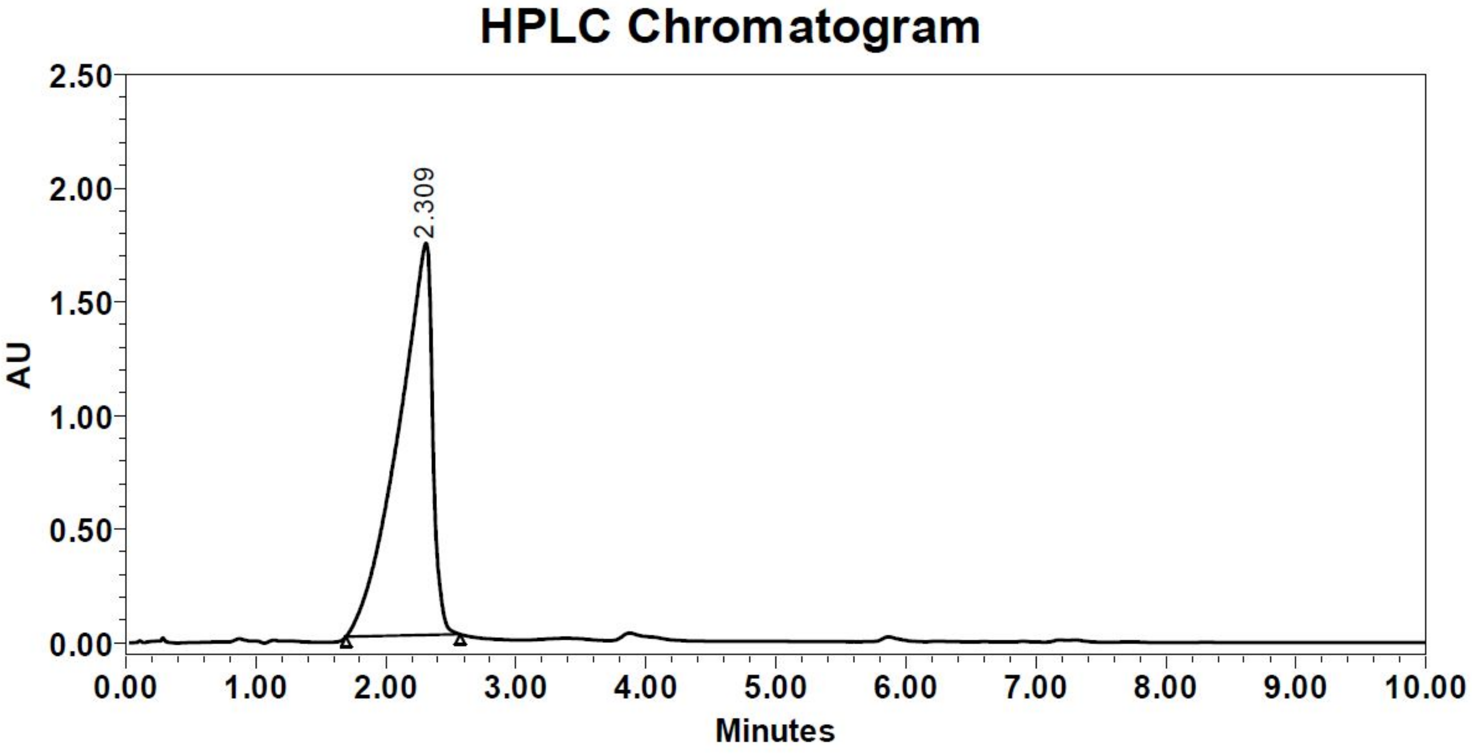

Sample Name: 13s

Vial: 10

Inj. #: 1

Date Acquired: 3/28/2016 2:32:02 PM IST

Processed Channel: 2998 Ch1 254nm@1.2nm

	Processed Channel	Retention Time (min)	Area	% Area	Height
1	2998 Ch1 254nm@1.2nm	2.309	31794415	100.00	1723526

Reported by User: System
Report Method: Sample Summary Table
Report Method IE 1479
Page: 1 of 1

Project Name: Sen Group
Date Printed:
5/28/2016
12:19:30 PM Asia/Calcutta

Compound 5j

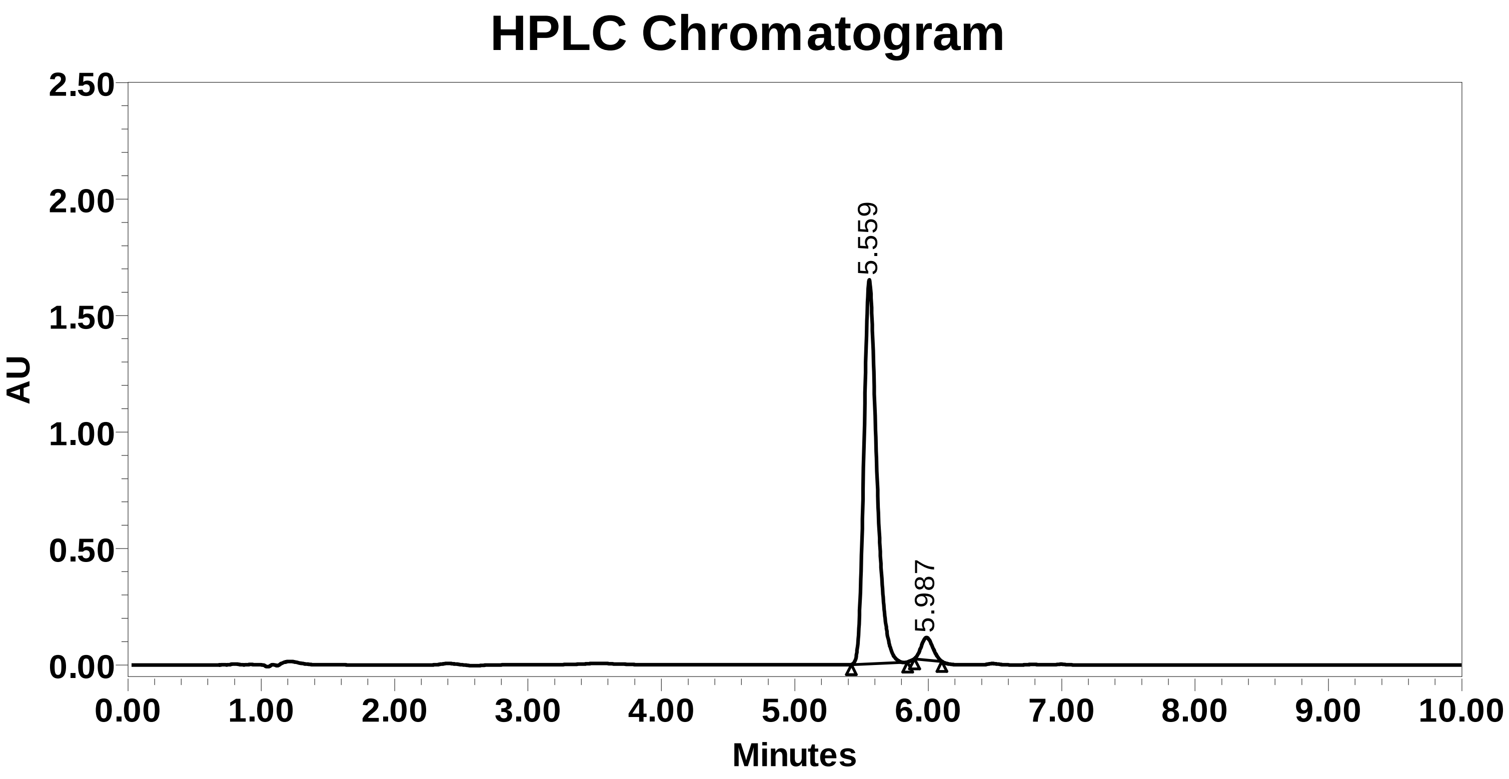

Sample Name: **14s**

Vial: 15

Inj. #: 1

Date Acquired: 3/28/2016 3:31:15 PM IST

Processed Channel: 2998 Ch1 254nm@1.2nm

	Processed Channel	Retention Time (min)	Area	% Area	Height
1	2998 Ch1 254nm@1.2nm	5.559	11064984	94.98	1647854
2	2998 Ch1 254nm@1.2nm	5.987	584412	5.02	96570

Compound 5k

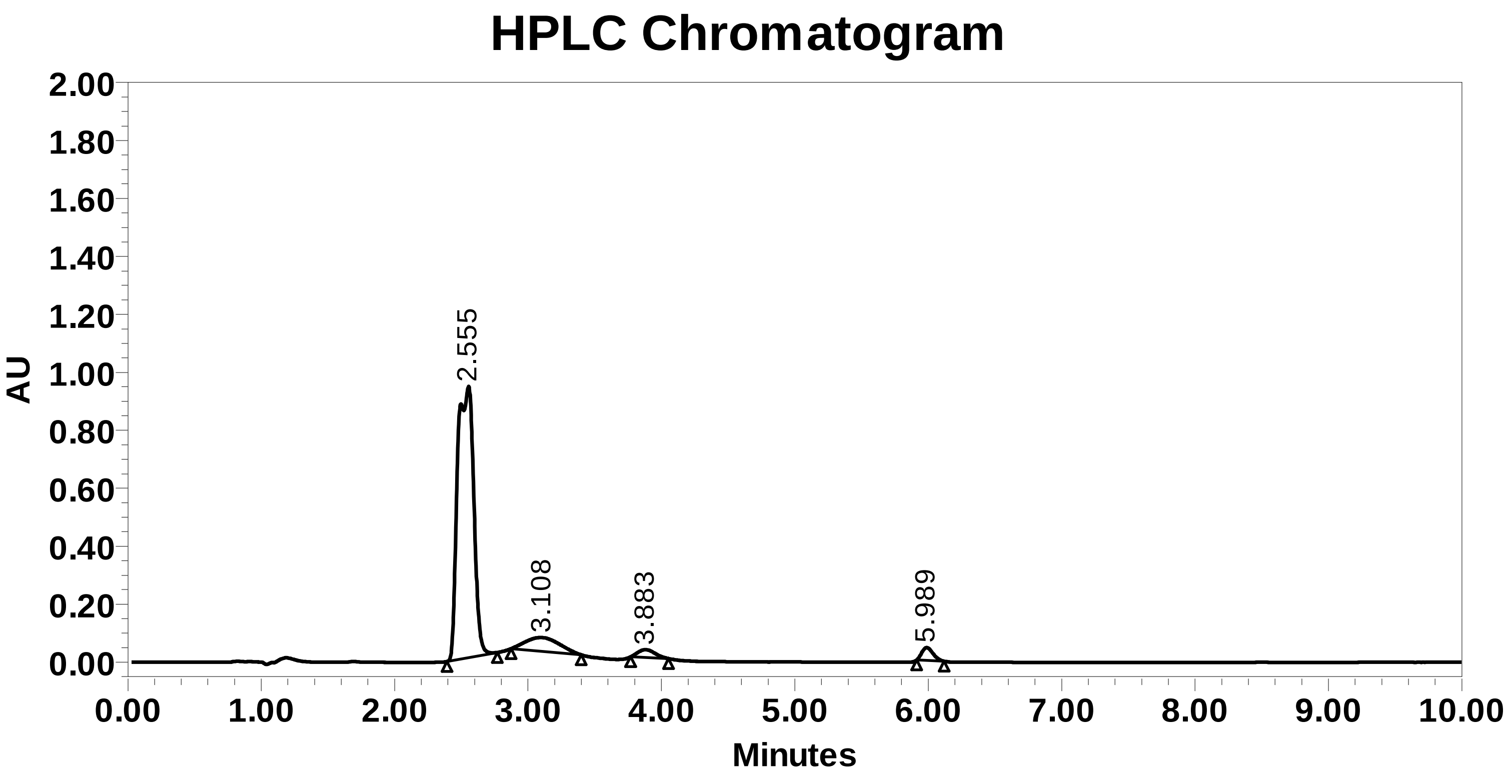

Sample Name: **16s**

Vial: 16

Inj. #: 1

Date Acquired: 3/28/2016 3:43:08 PM IST

Processed Channel: 2998 Ch1 254nm@1.2nm

	Processed Channel	Retention Time (min)	Area	% Area	Height
1	2998 Ch1 254nm@1.2nm	2.555	7904195	85.38	935925
2	2998 Ch1 254nm@1.2nm	3.108	847858	9.16	48034
3	2998 Ch1 254nm@1.2nm	3.883	245112	2.65	27597
4	2998 Ch1 254nm@1.2nm	5.989	259993	2.81	44729

Reported by User: System | Project Name: Sen Group

Report Method: Sample Summary Table | Date Printed:

Report Method I[illegible] 1479 | 5/28/2016

Page: 1 of 1 | 12:34:25 PM Asia/Calcutta

Compound 5l

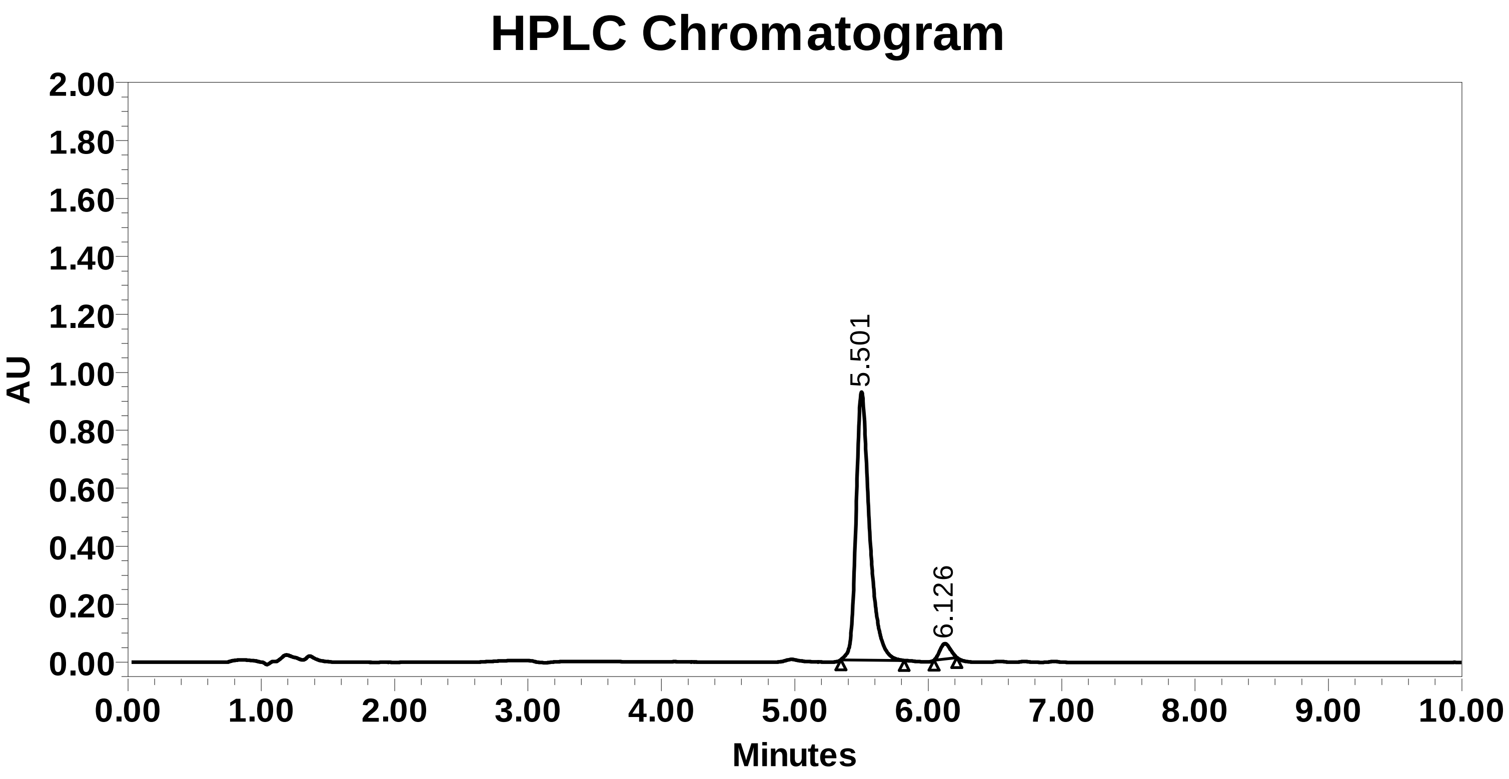

Sample Name: **18s**
Vial: 17
Inj. #: 1
Date Acquired: 3/28/2016 3:55:30 PM IST

Processed Channel: 2998 Ch1 254nm@1.2nm

	Processed Channel	Retention Time (min)	Area	% Area	Height
1	2998 Ch1 254nm@1.2nm	5.501	6542991	95.80	925003
2	2998 Ch1 254nm@1.2nm	6.126	287087	4.20	52529

Compound 5m

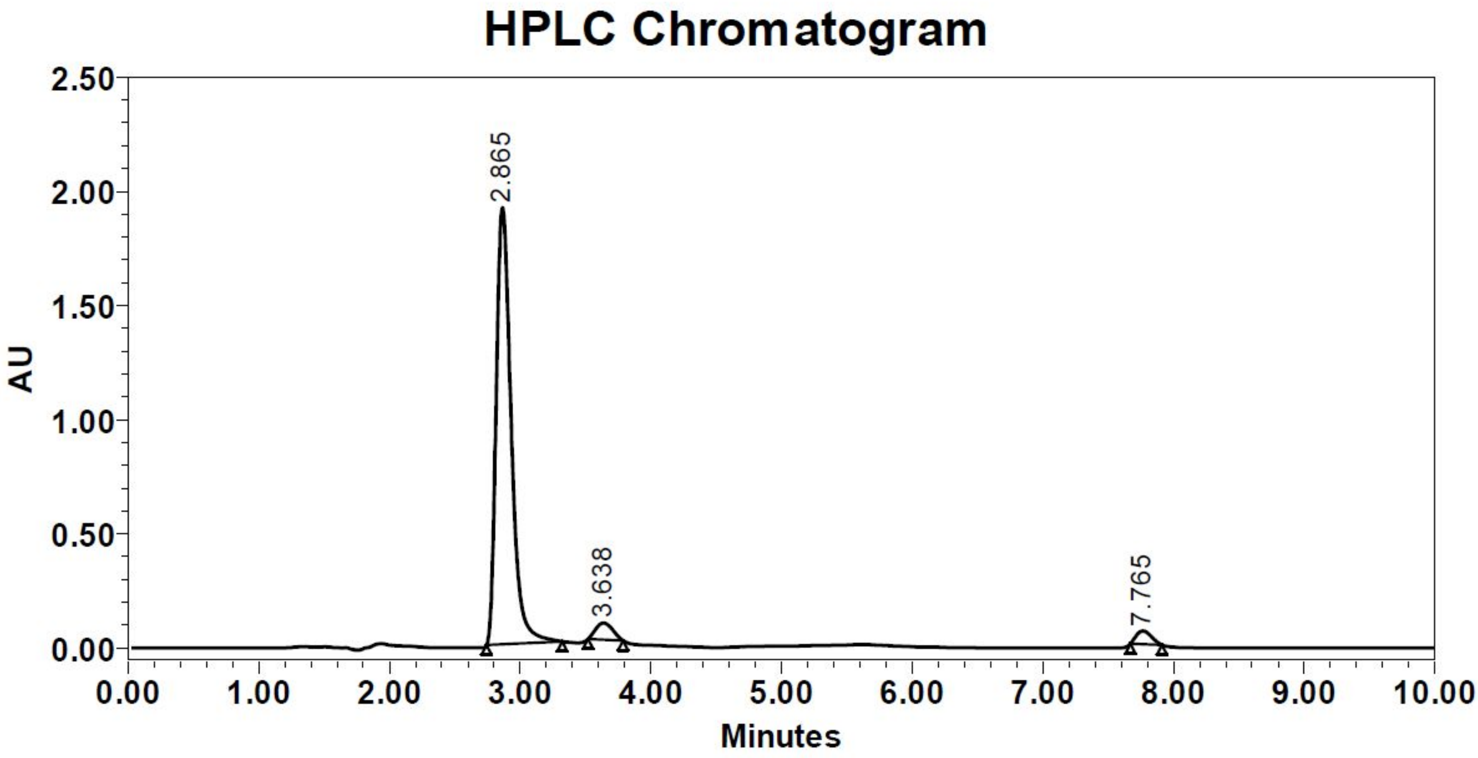

Sample Name: **10s**

Vial: 12

Inj. #: 1

Date Acquired: 3/28/2016 7:00:22 PM IST

Processed Channel: 2998 Ch1 254nm@1.2nm

	Processed Channel	Retention Time (min)	Area	% Area	Height
1	2998 Ch1 254nm@1.2nm	2.865	15207752	93.10	1912139
2	2998 Ch1 254nm@1.2nm	3.638	660592	4.04	73161
3	2998 Ch1 254nm@1.2nm	7.765	466828	2.86	58186

Reported by User: System
Report Method: Sample Summary Table
Report Method ID 1479
Page: 1 of 1

Project Name: Sen Group
Date Printed:
5/28/2016
12:31:38 PM Asia/Calcutta

HPLC-CHIP-MS.

Agilent 1260 infinity HPLC-Chip/MS system is a microfluidic chip-based technology incorporates peptide enrichment and separation and provides high-sensitive nano-spray. Charged peptides from HPLC-Chip system were directly infused into mass-spectrometer for detection. Following HPLC-Chip-MS conditions were used for acquiring the MS and MS/MS spectrum of the peptides.

Chip ID: G4240-62030

Chip Name: High Performace Chip, 360 nanoliter enrichment column, 150 mm X 75 µm separation column

Solvent A: 0.1% Formic Acid

Solvent B: 90% ACN / 10% (0.1% Formic Acid)

Flow Rate: 0.3 µl / min

Run Time: 65 minutes

Gradient: 0 min – 3%B

56 min – 40%B

60 min – 95%B

62 min – 95%B

62.1 min 3%B

65 min – 3%B

Sample Volume: 5 µl

MS Scan Range: 275 to 1700 m/z

MS Scan Rate: 8 spectra / sec

MS/MS Scan Rate: 3 spectra / sec

Ion Polarity: Positive Ions

Fragmentor Voltage: 170 V

Skimmer Voltage: 65 V

Octopole RF Voltage: 750V

Gas Temperature: 250°C

Drying Gas: 5 L / min

Cell migration assay

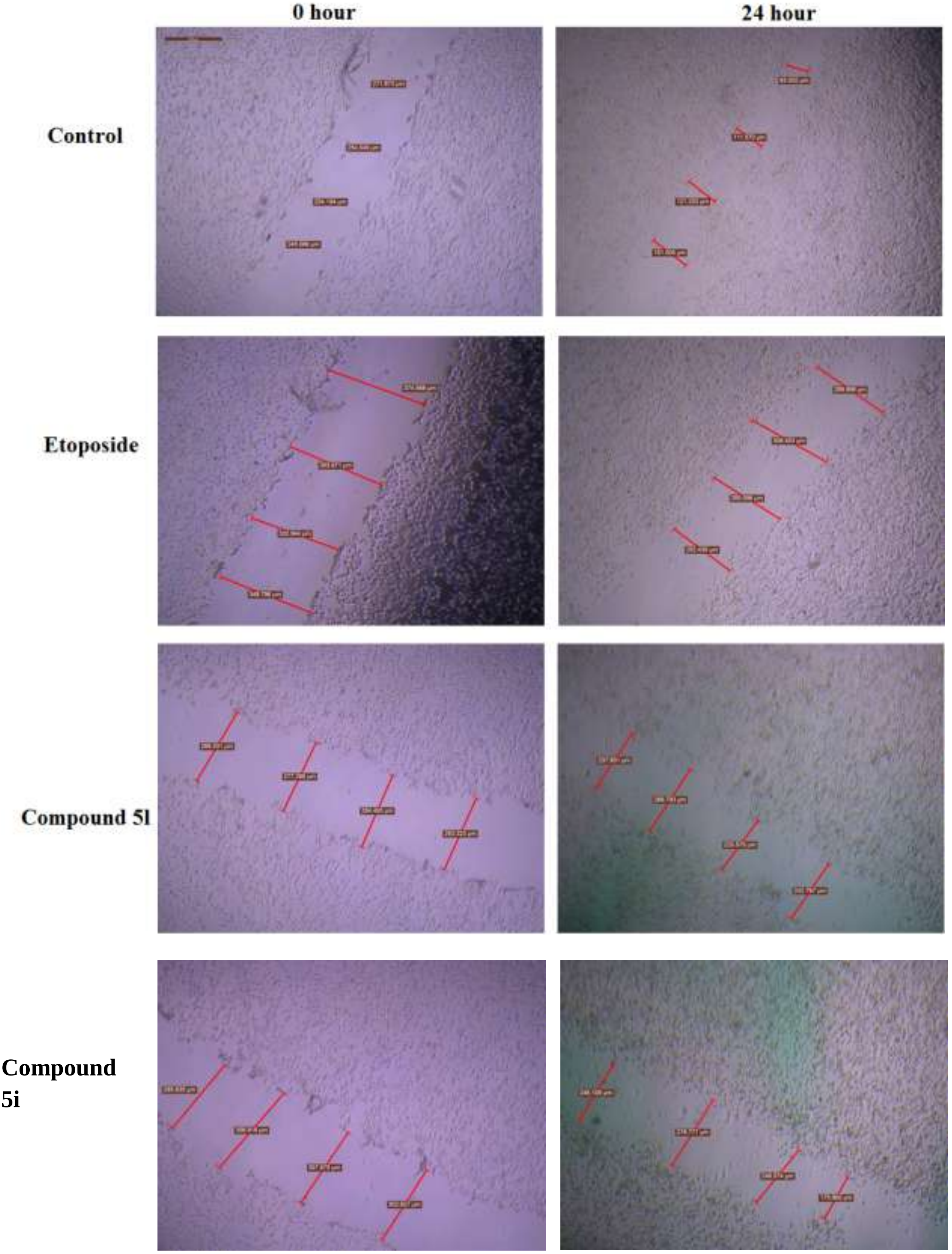

This note describes the application of the technology in identifying/de-convoluting true positive targets of a non-derivatized 'test' molecule BIS-III.

Background and Overall Goal

Bisindolylmaleimide-III (Bis-III) a known inhibitor of GSK3 protein and it induces apoptosis in the cancerous cell-lines. At 1μM, Bis-III inhibits 93% of PKCα kinase activity and also inhibits many other protein kinases including, S6K1, MAPKAP-K1, RSK2 and MSK1 with similar potency. Additionally, it inhibits PDK1, an important kinase in the insulin signaling pathway.

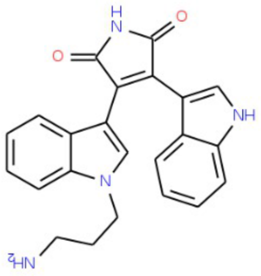

In following experiments Shantani's technology was utilized to identify primary and secondary targets of BIS-III.

Step-1) Immobilization of 'Bis-III' on Shantani's proprietary polymer matrix

1. Based on BIS-III compatibility with the polymer matrix; a 10 ml soluble stock solution of 0.3 mM BIS-III was prepared in HPLC water.

2. The molecule was quoted on the matrix in small amount (1 ml) and allowed to dry at RT. The coating and drying of membrane was carried out till the complete 10 ml solution of Bis-III was coated on the membrane.

Step-2) Capture and Identification of Targets

The molecule coated matrix was incubated with cell lysate for 2 minute. Excess lysate was removed and proteins were eluted with 2 ml elution buffer (1 mM Bis-III in TBST). Proteins were acetone precipitated, extracted and measured. Two similar experiments were performed - one for western blot analysis and another for Mass spec analysis.

Protein concentration from both control and test experiment was normalized and probed for GSK3-beta protein using western blot method.

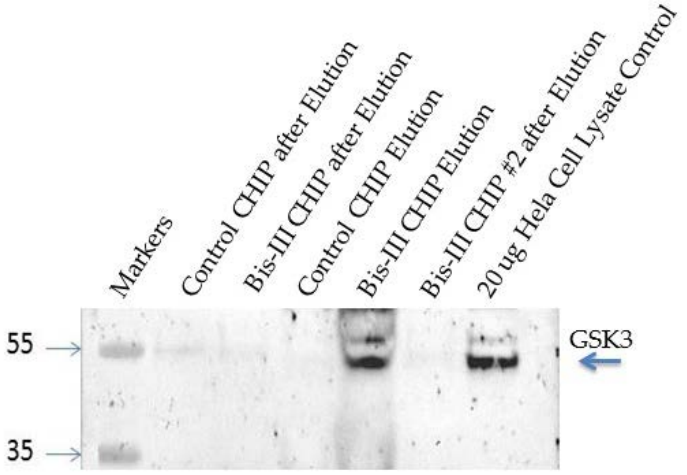

The target protein, GSK3-beta was specifically enriched on the Bis-III bound matrix.

Step-3) Deconvolution of Targets

Following the protocol (UPT - Technical Notes) proteins were identified using the mass-spectrometry based methods and specific targets were de-convoluted. Table 1 summarizes the outcome of target deconvolution experiments.

Uniprot ID	Protein Description	Maximum Number of Unique Peptides Identified	Protein Sequence Coverage (%)	Q-Value (%)
Q13418	Integrin-linked protein kinase	7	18.14	0
Q70UQ0	Inhibitor of nuclear factor kappa-B kinase	6	20.85	0
P28482	Mitogen-activated protein kinase 1	6	21.94	0
P60891	Ribose-phosphate pyrophosphokinase 1	5	20.44	0
E9PF82	Calcium/calmodulin-dependent protein kinase type II	4	10.46	0
P49841-2	Glycogen synthase kinase-3 beta	3	11.42	0
P63208	S-phase kinase-associated protein	3	19.63	0
P51570-2	Galactokinase	3	10.96	0

The primary target, GSK3-beta, of BIS-III was effectively captured using the described workflow. At the same time secondary targets of the molecule were also identified.

2'-(thiophen-2-yl)spiro[indoline-3,3'-pyrrolidin]-2-one (**5n**)

Following the general procedure tryptamine (200 mg, 1.25 mmol), thiophen-2-carbaldehyde (140.2 mg, 1.25 mmol), 1:1 THF/water (20 mL) with catalytic TFA and NBS (197.5 mg, 1.37 mmol) provided the desired compound **5n** in 200 mg (yield 59%) as yellow gummy solid. ^{1}H NMR (500 MHz; DMSO-d$_6$): 9.89 (s, 1H); 7.73-7.72 (d, *J* = 5 Hz, 1H); 7.54-7.52 (d, *J* = 10 Hz, 1H); 7.34-7.30 (m, 2H); 7.18-7.12 (m, 2H); 7.07-7.04 (t, *J* = 5 Hz, 1H); 4.11 (s, 1H); 3.49-3.46 (t, *J* = 10 Hz, 2H); 3.10-2.99 (m, 2H). ^{13}C NMR (125 MHz; DMSO-d$_6$): 178.2, 137.2, 136.4, 130.4, 128.8, 128.5, 127.4, 125.5, 122.2, 119.1, 111.6, 70.2, 59.7, 41.5. HRMS (ESI-TOF) m/z: $[M + H]^+$ Calcd for $C_{15}H_{15}N_2OS$ - 271.0900, Found - 271.072; IR 3151.43, 3016.39, 1663.65, 1240.36.

2'-(pyridin-4-yl)spiro[indoline-3,3'-pyrrolidin]-2-one (**5o**):

Following the general procedure tryptamine (200 mg, 1.25 mmol), pyridine-4-carbaldehyde (134 mg, 1.25 mmol), 1:1 THF/water (20 mL) with catalytic TFA and NBS (197.5 mg, 1.37 mmol) provided the desired compound **5o** in 212 mg (yield 64%) as light yellow solid. ^{1}H NMR (500 MHz; DMSO-d$_6$): 9.85 (s, 1H); 8.52-8.51 (d, *J* = 5 Hz, 2H); 7.43-7.41 (d, *J* = 10 Hz, 1H); 7.31-7.30 (d, *J* = 5 Hz , 2H); 7.25-7.23 (d, *J* = 10 Hz , 1H); 7.04-7.00 (t, *J* = 10 Hz, 1H); 6.98- 6.94 (t, *J* = 10 Hz, 1H); 4.09 (s, 1H); 2.99-2.95 (m, 2H); 2.71-2.67 (m, 2H). ^{13}C NMR (125 MHz; DMSO-d$_6$): 175.1, 151.6, 149.4, 136.00, 133.9, 126.7, 123.5, 120.7, 118.3, 111.0, 72.1, 59.8, 40.8. HRMS (ESI-TOF) m/z: $[M + H]^+$Calcd for $C_{16}H_{16}N_3O$ - 266.1288,Found - 266.1263; IR 3112.07, 1689.19, 1398.50.

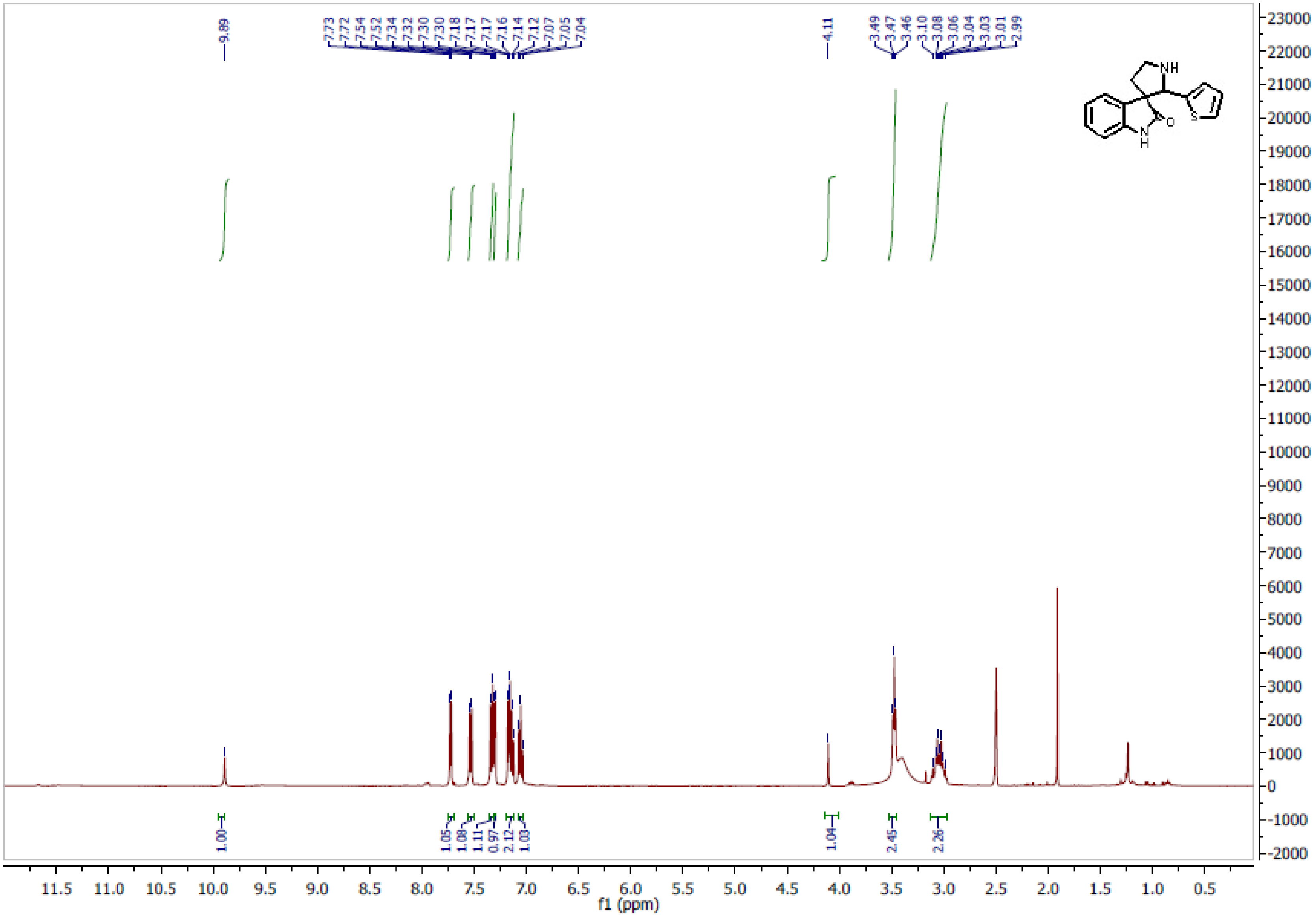

9.89
7.73
7.72
7.54
7.52
7.34
7.32
7.30
7.18
7.17
7.17
7.16
7.14
7.12
7.07
7.05
7.04
4.11
3.49
3.47
3.46
3.10
3.08
3.06
3.04
3.03
3.01
2.99
NH
O
N
H
S
1.00
1.05
1.08
1.11
0.97
2.12
1.03
1.04
2.45
2.26
f1 (ppm)

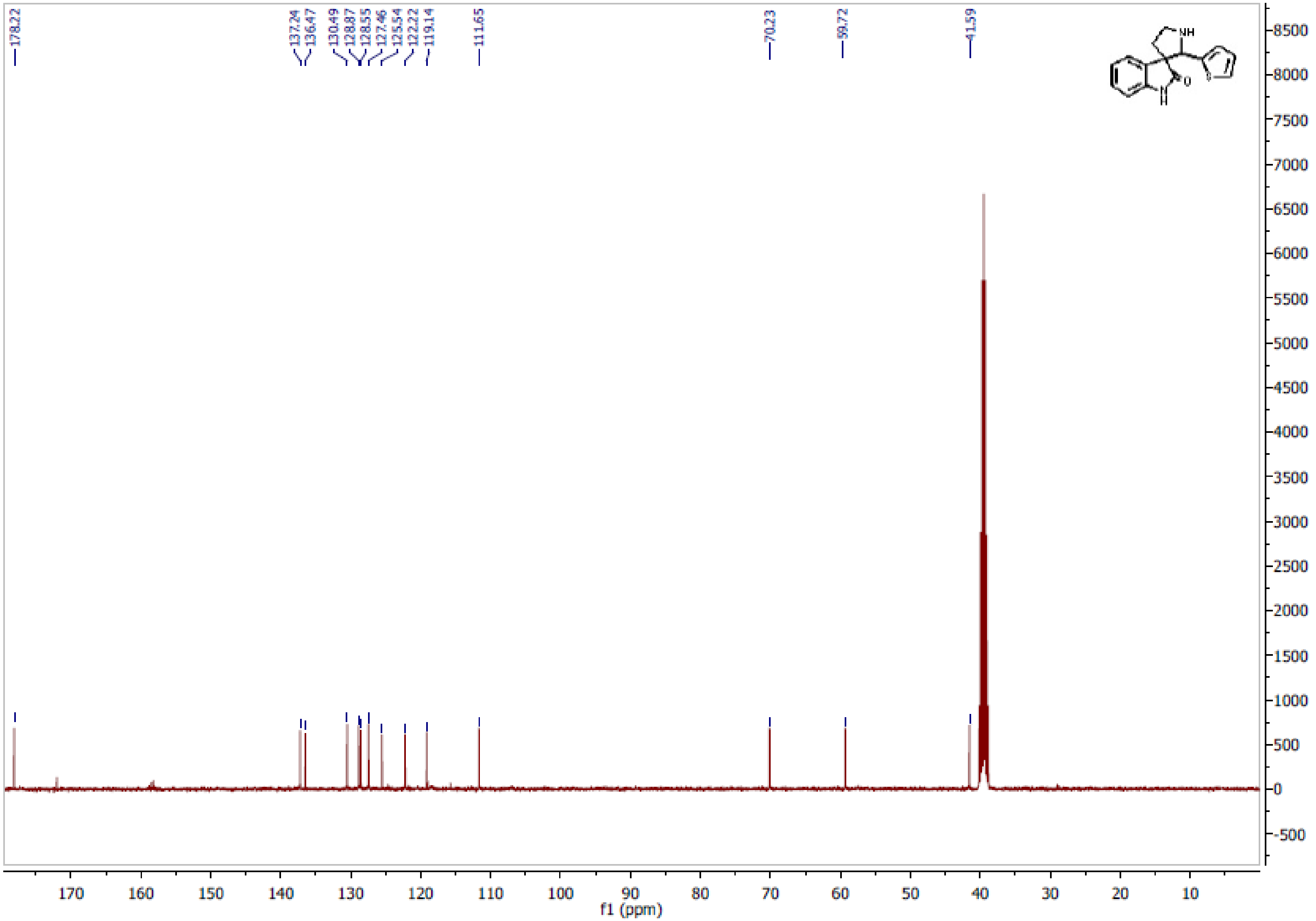
178.22
137.24
136.47
130.49
128.87
128.55
127.46
125.54
122.22
119.14
111.65
70.23
59.72
41.59
NH
O
N
H
S
f1 (ppm)

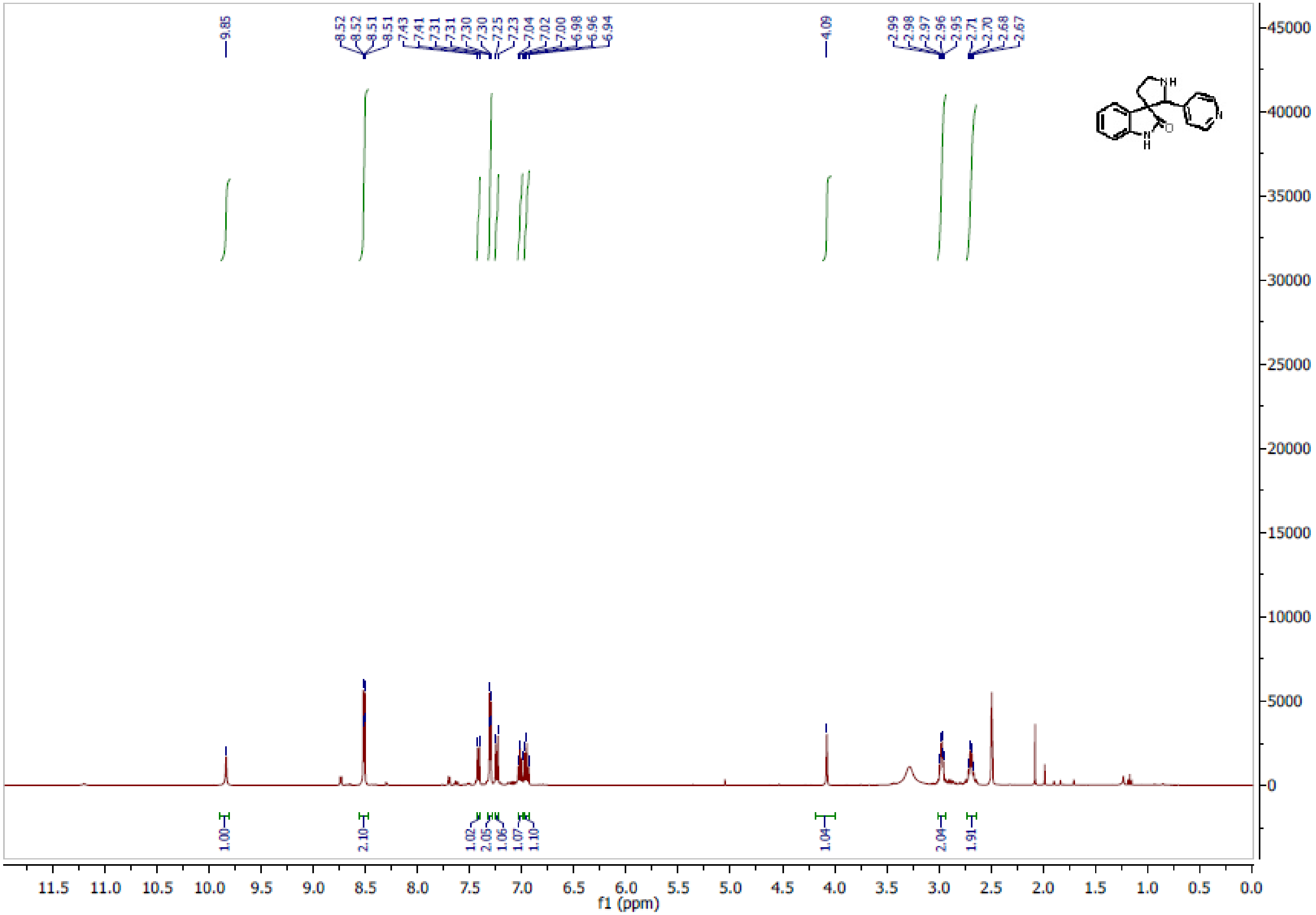
9.85
8.52
8.52
8.51
8.51
7.43
7.41
7.31
7.31
7.30
7.30
7.25
7.23
7.04
7.02
7.00
6.98
6.96
6.94
4.09
2.99
2.98
2.97
2.96
2.95
2.71
2.70
2.68
2.67
1.00
2.10
1.02
2.05
1.06
1.07
1.10
1.04
2.04
1.91
f1 (ppm)

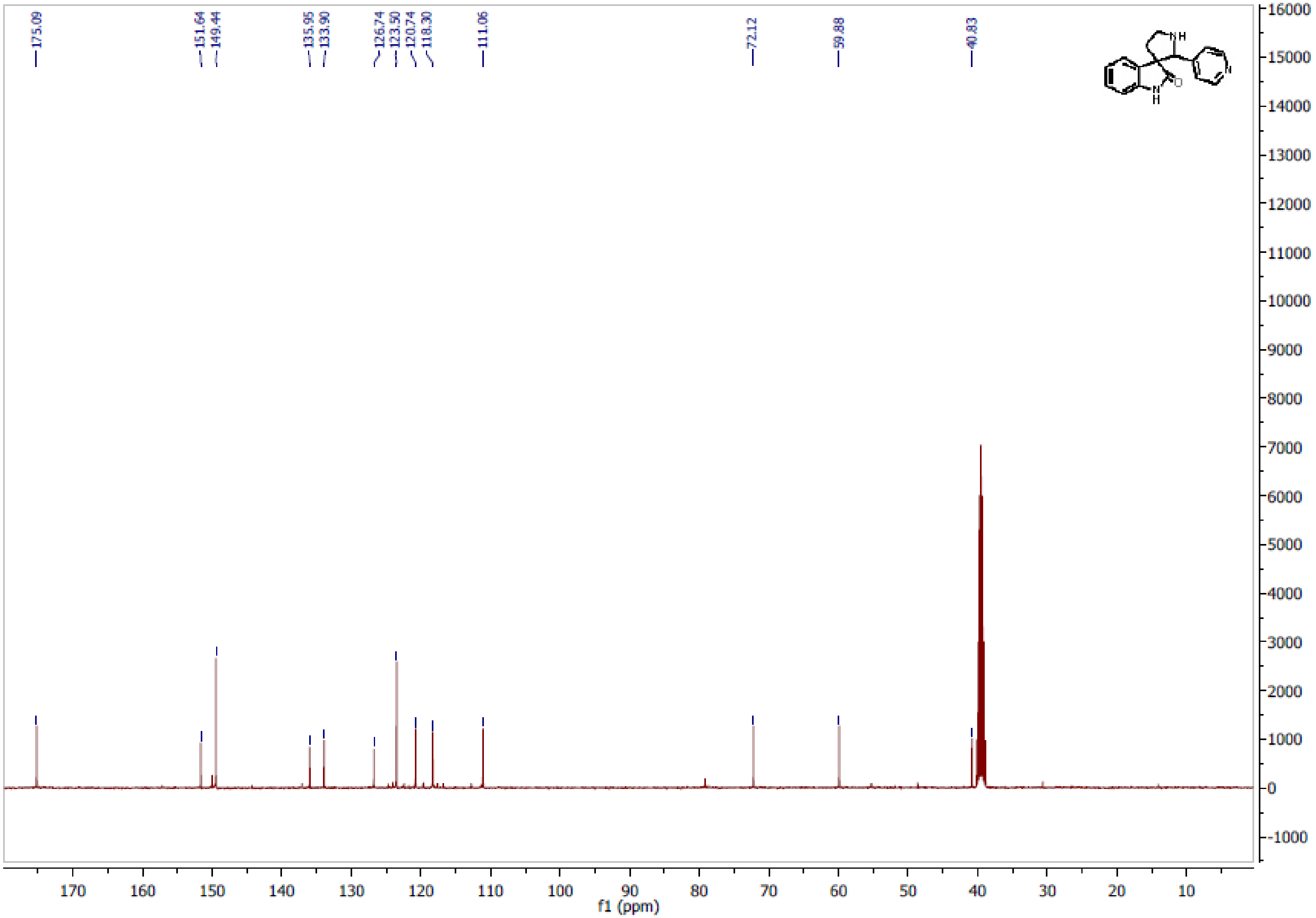
175.09
151.64
149.44
135.95
133.90
126.74
123.50
120.74
118.30
111.06
72.12
59.88
40.83
f1 (ppm)

Compound **5n**

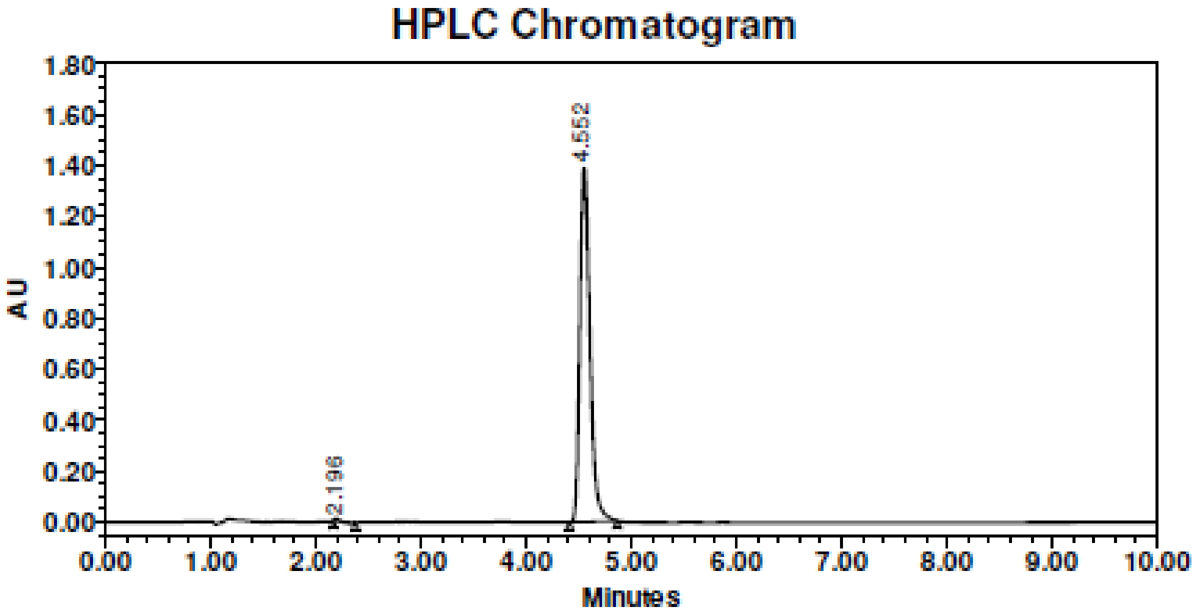

Sample Name: 19s

Vial: 13

Inj. #: 1

Date Acquired: 3/25/2016 4:06:51 PM IST

Processed Channel: 2998 Ch1 254nm@1.2nm

	Processed Channel	Retention Time (min)	Area	% Area	Height
1	2998 Ch1 254nm@1.2nm	2.196	47202	0.51	10685
2	2998 Ch1 254nm@1.2nm	4.552	9162457	99.49	1388411

Reported by User: System
Report Method: Sample Summary Table
Report Method ID: 1479
Page: 1 of 1

Project Name: Sen Group
Date Printed:
7/12/2016
11:00:05 PM Asia/Calcutta

Compound **5o**

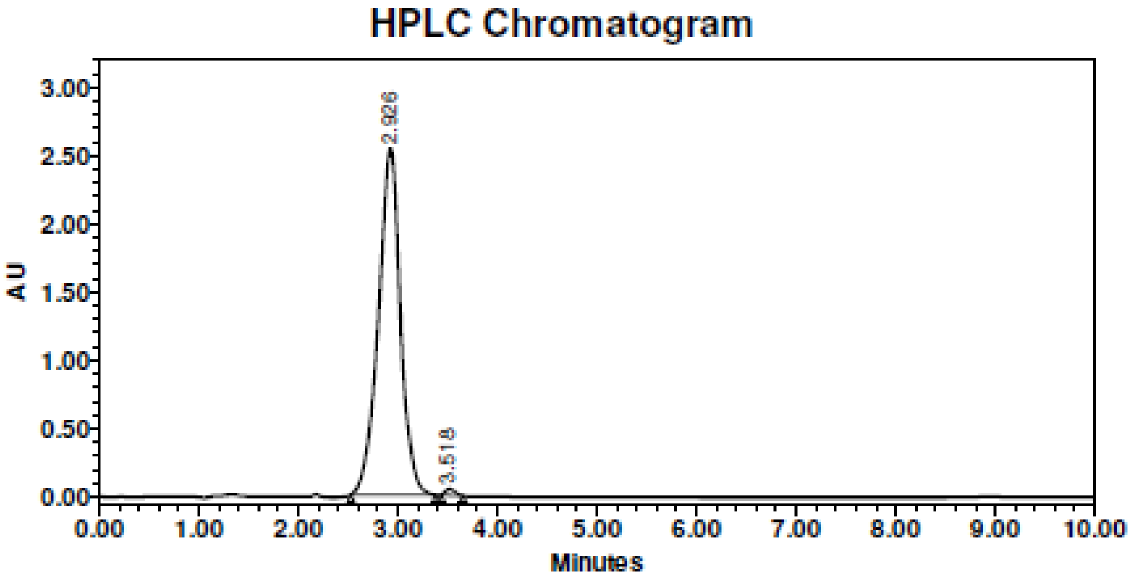

Sample Name: 20s

Vial: 15

Inj. #: 1

Date Acquired: 3/25/2016 4:28:35 PM IST

Processed Channel: 2998 Ch1 254nm@1.2nm

	Processed Channel	Retention Time (min)	Area	% Area	Height
1	2998 Ch1 254nm@1.2nm	2.926	39105930	99.24	2534579
2	2998 Ch1 254nm@1.2nm	3.518	298714	0.76	47413

Reported by User: System
Report Method: Sample Summary Table
Report Method ID: 1479
Page: 1 of 1

Project Name: Sen Group
Date Printed:
7/12/2016
10:54:48 PM Asia/Calcutta

Graphical depiction of the cell migration assay

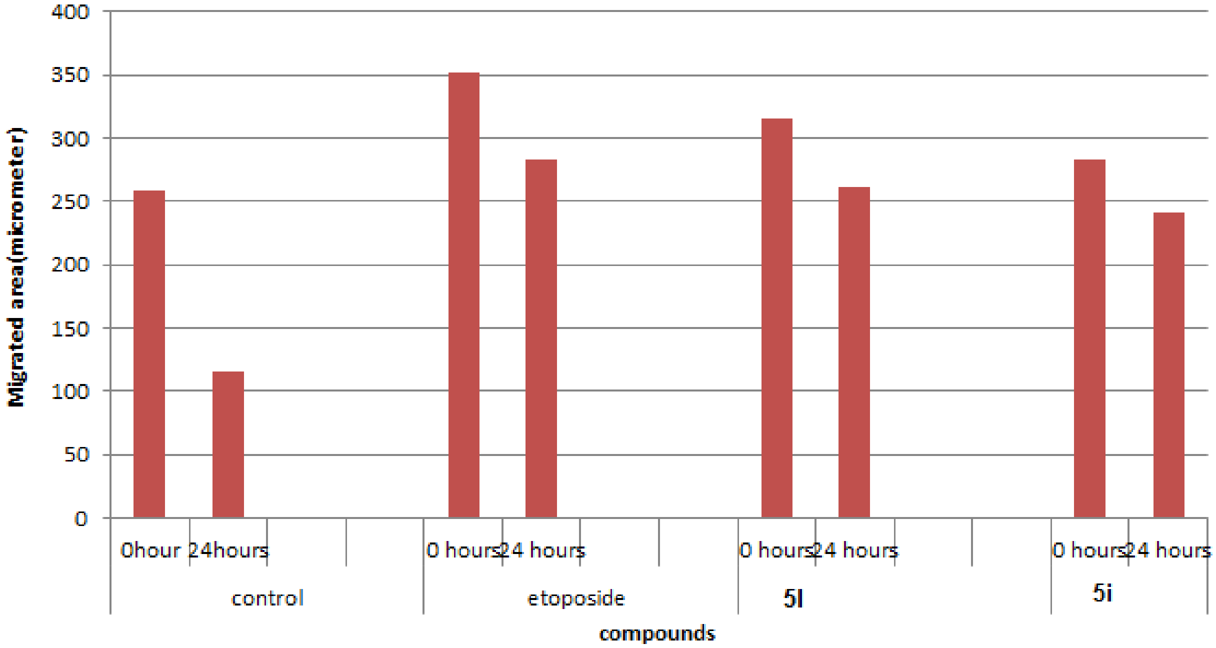

Cytotoxicity assay with MCF 10A

					Experiment :MCF10A cytotoxicity assay 48 hours							
Raw Data{Wavelength:595.0}												
	1	2	3	4	5	6	7	8	9	10	11	12
A	1.279	1.218	1.002	0.042	0.064	0.061	0.068	0.037	0.036	0.037	0.039	0.036
B	0.519	0.3	0.438	0.038	0.065	0.061	0.063	0.039	0.041	0.038	0.037	0.065
C	1.154	1.203	0.985	0.037	0.152	0.072	0.066	0.039	0.037	0.038	0.038	0.039
D	1.347	1.219	0.902	0.039	0.067	0.075	0.08	0.04	0.037	0.039	0.038	0.037
E	1.118	0.944	0.922	0.04	0.083	0.081	0.078	0.04	0.041	0.04	0.037	0.041
F	1.005	1.109	1.382	0.037	0.074	0.079	0.085	0.04	0.04	0.037	0.037	0.036
G	0.063	0.072	0.072	0.039	0.069	0.073	0.071	0.039	0.04	0.039	0.039	0.039
H	0.083	0.071	0.074	0.037	0.041	0.042	0.039	0.038	0.04	0.039	0.036	0.037
	O.D1	O.D2	O.D3	AVG	CTRL-TEST	CTRL-TST/(	*100					
CONTROL	1.279	1.218	1.002	1.166	0	0	0					
DMSO	0.519	0.3	0.438	0.419	0.747333333	0.640755	64.07					
**5e**	1.154	1.203	0.985	1.114	0.052333333	0.04487	4.48					
**5i**	1.347	1.219	0.902	1.156	0.010333333	0.00886	0.8					
**5l**	1.005	1.109	1.382	1.165	0.001	0.000857	0.08					
